# Altered TAOK2 activity causes autism-related neurodevelopmental and cognitive abnormalities through RhoA signaling

**DOI:** 10.1038/s41380-018-0025-5

**Published:** 2018-02-21

**Authors:** Melanie Richter, Nadeem Murtaza, Robin Scharrenberg, Sean H. White, Ole Johanns, Susan Walker, Ryan K. C. Yuen, Birgit Schwanke, Bianca Bedürftig, Melad Henis, Sarah Scharf, Vanessa Kraus, Ronja Dörk, Jakob Hellmann, Zsuzsa Lindenmaier, Jacob Ellegood, Henrike Hartung, Vickie Kwan, Jan Sedlacik, Jens Fiehler, Michaela Schweizer, Jason P. Lerch, Ileana L. Hanganu-Opatz, Fabio Morellini, Stephen W. Scherer, Karun K. Singh, Froylan Calderon de Anda

**Affiliations:** 1 Center for Molecular Neurobiology Hamburg (ZMNH), Research Group Neuronal Development, University Medical Center Hamburg-Eppendorf, Hamburg, Germany; 2 Stem Cell and Cancer Research Institute, McMaster University, Hamilton, Ontario Canada; 3 Department of Biochemistry and Biomedical Sciences, Michael G. DeGroote School of Medicine, Faculty of Health Sciences, McMaster University, Hamilton, Ontario Canada; 4 The Centre for Applied Genomics and Program in Genetics and Genome Biology, Peter Gilgan Centre for Research and Learning, The Hospital for Sick Children, Toronto, Ontario Canada; 5 Department of Molecular Genetics and McLaughlin Centre, University of Toronto, Toronto, Ontario Canada; 6 Department of Anatomy and Histology, Faculty of Veterinary Medicine, Assiut University, Assiut, Egypt; 7 Center for Molecular Neurobiology Hamburg (ZMNH), Research Group Behavioral Biology, University Medical Center Hamburg-Eppendorf, Hamburg, Germany; 8 Mouse Imaging Center, The Hospital for Sick Children, Toronto, Ontario Canada; 9 Department of Medical Biophysics, University of Toronto, Toronto, Ontario Canada; 10 Developmental Neurophysiology, Institute of Neuroanatomy, University Medical Center Hamburg-Eppendorf, Hamburg, Germany; 11 Department of Neuroradiology, University Medical Center Hamburg-Eppendorf, Hamburg, Germany; 12 Center for Molecular Neurobiology Hamburg (ZMNH), Core Facility Morphology and Electronmicroscopy, University Medical Center Hamburg-Eppendorf, Hamburg, Germany; 13 Present Address: Laboratory of Neurobiology, Department of Biosciences, University of Helsinki, Helsinki, Finland

**Keywords:** Neuroscience, Autism spectrum disorders

## Abstract

Atypical brain connectivity is a major contributor to the pathophysiology of neurodevelopmental disorders (NDDs) including autism spectrum disorders (ASDs). *TAOK2* is one of several genes in the 16p11.2 microdeletion region, but whether it contributes to NDDs is unknown. We performed behavioral analysis on *Taok2* heterozygous (Het) and knockout (KO) mice and found gene dosage-dependent impairments in cognition, anxiety, and social interaction. *Taok2* Het and KO mice also have dosage-dependent abnormalities in brain size and neural connectivity in multiple regions, deficits in cortical layering, dendrite and synapse formation, and reduced excitatory neurotransmission. Whole-genome and -exome sequencing of ASD families identified three de novo mutations in TAOK2 and functional analysis in mice and human cells revealed that all the mutations impair protein stability, but they differentially impact kinase activity, dendrite growth, and spine/synapse development. Mechanistically, loss of *Taok2* activity causes a reduction in RhoA activation, and pharmacological enhancement of RhoA activity rescues synaptic phenotypes. Together, these data provide evidence that TAOK2 is a neurodevelopmental disorder risk gene and identify RhoA signaling as a mediator of TAOK2-dependent synaptic development.

## Introduction

Thousand and one amino-acid kinase 2 (*TAOK2*) is a family member of the mammalian sterile 20 (STE20)-like kinases and is implicated in neurodevelopmental disorders (NDDs) [1–4]. *TAOK2* is located in the autism spectrum disorder (ASD) and schizophrenia-associated 16p11.2 chromosomal deletion region [5–8] and is associated with other neurodevelopmental phenotypes [9]. *TAOK2* is also present in the 16p11.2 duplication syndrome, which has distinct and reciprocal neurological phenotypes [9]. The 16p11.2 region also harbors *KCTD13*, *MAPK3*, and *SEZ6L2*, which may contribute to neurological phenotypes [7, 10–13]. Additional evidence comes from a genome-wide association study for psychosis that identified a significant single-nucleotide polymorphism in *TAOK2* [14]. Finally, *TAOK2* mRNA is a target of fragile X mental retardation protein (FMRP) [15]. Despite these suggestive studies, there is no direct evidence using mouse models or human cell models that genetic alterations in *TAOK2* cause NDDs.

Studies of ASD have uncovered many genes and signaling pathways, and one of the leading hypotheses implicates altered synapse formation and plasticity. Large genetic sequencing studies have revealed that mutations in different synaptic genes can cause ASD or specific NDDs [16–23]. Affected synaptic pathways include adhesion molecules [24, 25], regulatory translational proteins [26–30], ion channels [18, 23, 31], and cytoskeleton mediators [32–36]. Pharmacological treatment ameliorated some of these genetic forms of ASD providing the framework to identify novel therapies [37–41].

There are two isoforms of murine *Taok2*, α and β, but isoform-specific roles in the brain remain poorly studied. Most STE20s activate one or more of the mitogen-activated protein kinases (MAPKs), including c-Jun N-terminal kinase (JNK) and p38, although their biological responses do not always require MAPKs [42]. TAOK2 can regulate microtubule dynamics and organization [43] and auto-phosphorylation of TAOK2 is important to initiate kinase activity [44]. Reduced Taok2 expression revealed a decrease in axonal growth and basal dendrite formation of excitatory neurons in the mouse cortex [45]. TAOK2 is also necessary during activity-dependent synapse growth [46]. Furthermore, phosphorylation of TAOK2 by Mammalian  STE20-like kinase 3 (MST3)  regulates synapse development through an association with Myosin Va [47] or its activation of Septin7 kinase, which stabilizes PSD95 [48]. These studies indicate that TAOK2 plays an emerging role in dendritic arborization and synapse maturation, suggesting that changes in TAOK2 activity may lead to abnormal synaptic connectivity and behavioral phenotypes. However, previous studies utilized incomplete knockdown methods to study TAOK2, leaving in question the importance of TAOK2 in vivo during neural circuit function and behavior.

In this study, we combined a heterozygous (Het) and knockout (KO) mouse model with genetic sequencing of ASD subjects to dissect the role of TAOK2 in neural circuit development (summarized in Supplementary Fig. 18b). Analysis of *Taok2* Het and KO mice revealed several gene dosage-dependent impairments in behavior, whole-brain connectivity, cortical layering, neuronal morphology, and synaptic function in cortical excitatory neurons similar to other models [49–53]. We also report novel missense and truncating loss-of-function (LOF) mutations in *TAOK2* in ASD subjects that differentially impair TAOK2 function and disrupt dendrite formation and synapse structure. Further examination showed a *Taok2*-dependent reduction in RhoA activity mediating synaptic defects, which can be rescued pharmacologically by enhancing RhoA activity. Taken together, our study reveals that Taok2 is critical for neural circuit formation and function in an animal model, and the analysis of TAOK2 human mutations provide insight into the etiology of NDDs.

## Materials and methods

### Animals

*C57BL6/J Taok2* KO (*Taok2* -/-) mice were generated and described by Kapfhamer et al. [54]. Animals were housed at the Central Animal Facilities at McMaster University and University Medical Center Hamburg-Eppendorf, Hamburg. All procedures received the approval of the Animal Research Ethics Board (AREB) and the Institutional Animal Care and Use committee of the City of Hamburg, Germany (G48/13 and G43/16 acc. to the Animal Care Act, §8 from 18 May 2006). See supplementary methods for further detail.

### Magnetic resonance imaging (MRI) of live mice

Live MRI was performed using a dedicated 7 Tesla small animal MRI (ClinScan, Bruker, Ettlingen, Germany) with a mouse head four element phased array receiver surface coil and a linear polarized rat body transmit coil. Mouse imaging was done at the Neuroradiology/University Medical Center Hamburg-Eppendorf based on previous publications [55–57]. See supplementary methods for further detail.

### In vivo electrophysiology

Mouse pups were initially anesthetized with isoflurane, fixed into the stereotaxic apparatus, and local anesthetic was administered. Multi-site electrodes (NeuroNexus, Ann Arbor, MI) were inserted to the skull surface into the prefrontal cortex (PFC) and hippocampus (HC). Two silver wires were inserted into cerebellum and served as ground and reference electrodes. Simultaneous recordings of local field potential (LFP) and multi-unit activity were performed from the prelimbic subdivision of the PFC and the CA1 area of the intermediate HC as described [58]. See supplementary methods for further detail.

### In situ electrophysiology

Coronal brain slices (400 µm) were prepared and miniature excitatory postsynaptic currents were recorded as previously described [35]. For miniature inhibitory postsynaptic currents recordings, 1 µM tetrodotoxin and 1 mM kynurenic acid was used in bathing medium. See supplementary methods for further detail.

### Behavior analysis

Behavioral experiments were performed with 10- to 18-week-old mice during the dark cycle in a room illuminated with dim red light. Tests started and ended at least 2 h after light offset and 3 h before light onset, respectively. Tracks representing the position of the mice were created and analyzed with EthoVision (Noldus, Wageningen, The Netherlands) [59]. Manual scoring of behavior was performed by a trained experimenter blinded to the genotype of the mice using *The Observer* software (Noldus). See supplementary methods for further detail on the behavioral tests we used that are described previously [60–62].

### Electron microscopy

Coronal vibratome sections of the cingulate cortex (cg1 and cg2) and the prelimbic cortex (PL) of the PFC, the primary somatosensory regions S1HL, S1Fl, S1BF, and the intermediate HC were collected and prepared for electron microscopy as described [63]. Semithin sections (0.5 µm) were prepared for light microscopy mounted on glass slides and stained for 1 min with 1% Toluidine blue. Ultrathin sections (60 nm) were examined in an EM902 (Zeiss, Munich, Germany). Pictures were taken with a MegaViewIII digital camera (A. Tröndle, Moorenweis, Germany).

### Analysis of dendritic and spine morphology and spine motility

Sholl analysis was conducted using the semi-automatized Simple Neurite Tracer plug-in on Fiji (ImageJ) and analyzed using the Sholl analysis plug-in. To quantify dendrite spine morphology, image stacks were uploaded to Fiji and a semi-automatized protocol [64] was used for categorization based on spine-head width and spine length. For spine motility assays, cultures were imaged at ×63 magnification and recorded at a 2-s frame rate for 5 min. Raw images were uploaded to Fiji and analysis was done by the Dendritic Filipodia Motility Analyzer [65]. For further detailed description of analyses, please refer to supplementary methods.

### Sample collection and whole-genome sequencing of ASD families

We obtained informed consents, or waivers of consent, which were approved by the Western Institutional Review Board, Montreal Children’s Hospital—McGill University Health Centre Research Ethics Board, McMaster University—Hamilton Integrated Research Ethics Board, Eastern Health Research Ethics Board, Holland Bloorview Research Ethics Board, and the Hospital for Sick Children Research Ethics Board. Whole-genome sequencing was performed as previously described in [66].

### Biochemical assays for protein expression, activity, and interaction

For detailed description of the analyses, please refer to supplementary methods.

### Statistical analysis

Data are expressed as mean ± s.e.m. Minimums of three mice per condition, or three mouse litters for in vitro culture experiments, were used for statistical analysis. We used the Student’s unpaired *t-*test, Wilcoxon signed-rank pair test, one-sample* t*-test, one-way analysis of variance (ANOVA), two-way ANOVA, mixed three-way ANOVA and post hoc Tukey, Dunnett, and Bonferroni tests in GraphPad Prism 7 statistical software for statistical analyses. Dunnett’s test was utilized in all cases to compare all conditions with the control conditions, except when it was necessary to compare multiple conditions, in which case the Tukey test was utilized. Bonferroni’s test was used for all behavior tests due to sample size and differences in variance. The *p*-values in the figure legends are from the specified tests, and *p* < 0.05 was considered statistically significant.

### Data availability

Whole-genome data sets were generated during and analyzed during the current study are available at the MSSNG repository, http://research.mss.ng.

## Results

### Taok2 KO mice display brain morphological and behavioral abnormalities

**Fig. 1:**
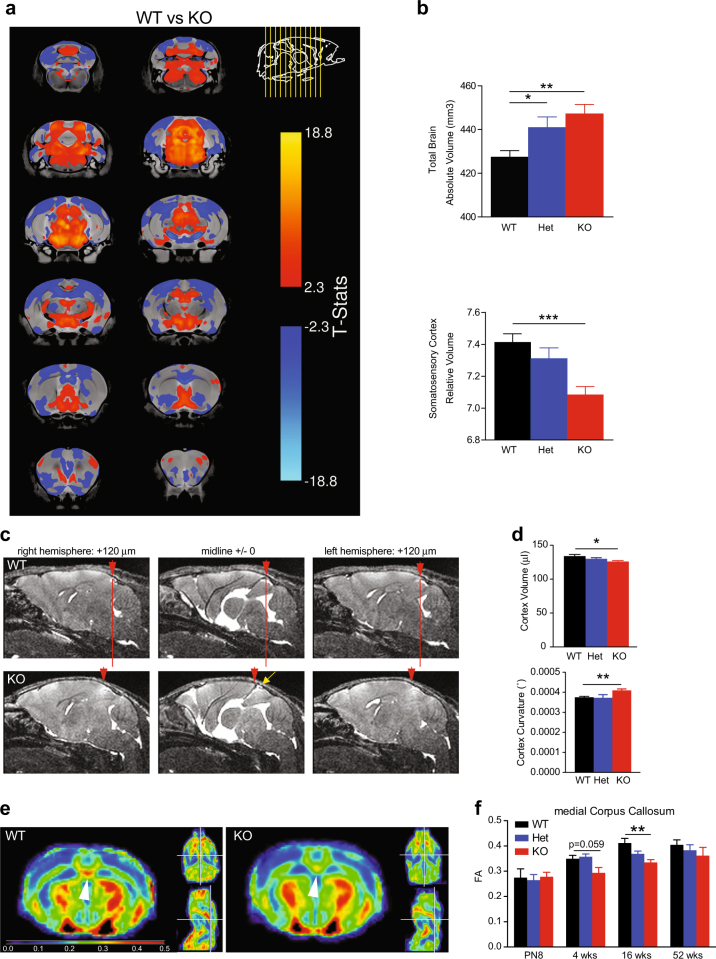
*Taok2* KO mice have altered brain morphology and brain activity. **a** Voxel-wise analysis highlighting significant differences in relative volume (images show the lowest threshold of 5% false discovery rate (FDR) for *Taok2* KO mice) throughout the brain between the WT and *Taok2* KO mice. T-statistic of 2.3–18.8 indicates decreasing false discovery rate, where 2.3 = 5% FDR and positive or negative T-stat indicates positive or negative change compared with WT brain. **b** Top: *Taok2* KO mice have increased absolute brain volume compared with WT mice (WT = 16, Het = 13, KO = 23 mice from three different cohorts, statistics by linear model; WT vs. KO *p* = 0.0015, WT vs. Het *p* = 0.0223). Bottom: the relative volume of the somatosensory cortex is reduced in *Taok2* KO mice (WT = 16, Het = 13, KO = 23 mice from three different cohorts, statistics by linear model corrected for multiple comparisons using FDR; WT vs. KO *p *= 0.0002, WT vs. Het *p* = 0.5569). **c** *Taok2* KO mice show shortening and increased curvature of the cortex at 4 weeks of age in vivo. Red arrowheads indicate dorsal end of the cortex, red line indicates shortening of the *Taok2* KO cortex, and yellow arrow indicates gap between cortex and colliculi. **d** Top: decreased cortex volume (µl) in *Taok2* KO mice (WT = 7, Het = 12, KO = 11 mice from three different cohorts; one-way ANOVA, post hoc Dunnett’s test; *F*_2, 27 _= 4.369, *p* = 0.0027; WT vs. KO *p *= 0.0129). Bottom: increased curvature of the cortex (° degree × e^−006^) in *Taok2* KO mice brains (WT = 7, Het = 12, KO = 11 from three different cohorts; one-way ANOVA, post hoc Dunnett’s test; *F*_2, 27 _= 3.142, *p *= 0.0593; unpaired *t*-test; WT vs. KO *p *= 0.0025). **e** Representative diffusion tensor images of WT and *Taok2* KO mouse brains. White arrowhead indicates reduced fiber density in medial corpus callosum region in *Taok2* KO mice. Blue to red indicates increased fractional anisotropy **f** Reduced fiber track density measured by fractional anisotropy (FA) in the medial corpus callosum of *Taok2* KO mice brains. (PN8: WT = 6, Het = 10, KO = 7; 4 weeks: WT = 7, Het = 12, KO = 10; 16 weeks: WT = 7, Het = 15, KO = 11; 52 weeks: WT = 5, Het = 6, KO = 6 mice from three cohorts; one-way ANOVA, post hoc Dunnett’s test; PN8: *F*_2, 20_ = 0.08947, *p* = 0.9148; 4 weeks: *F*_2, 26_ = 4.832,* p *= 0.0164, WT vs. KO *p* = 0.0598; 16 weeks: *F*_2, 30 _= 7.241,* p* = 0.0027, WT vs. KO *p *= 0.0013; 52 weeks: *F*_2, 14 _= 0.6358,* p* = 0.5441). *p<0.05, **p<0.01, and ***p<0.001. Values are mean +/- s.e.m.

We analyzed the anatomy of *Taok2* KO brains using MRI on fixed 8- to 10-week-old mouse brains and examined 182 independent regions. The absolute brain volume of *Taok2* KO mice was significantly enlarged compared with WT mice (Figs. 1a, b) derived from absolute and relative volumetric increases in the hindbrain, midbrain, hypothalamus, thalamus, cerebellum, and HC (Supplementary Fig. 1i and Supplementary Table 1) but a relative decrease in the somatosensory cortex (Figs. 1a, b and Supplementary Table 1) suggesting that the increase in brain volume is caused primarily by these regions. We also found significant decreases in the relative brain volumes of the corpus callosum, many cortical regions, the anterior commissure, and the olfactory bulbs (Supplementary Fig. 1j and Supplementary Table 1). *Taok2* Het mice also showed significant increases in brain volume, but not as dramatic as KO mice, consistent with a gene dosage effect (Fig. 1b). Furthermore, *Taok2* Het mice show trends (*p-*value < 0.05, but false discovery rate (FDR)-adjusted *p*-value > 0.05) similar to *Taok2* KO mice in absolute or relative volumes of regions such as the midbrain, thalamus, hypothalamus, and hindbrain regions, suggesting these regions are strongly affected by loss of Taok2 (Supplementary Fig. 1f, i and Supplementary Table 1). The same differences and trends were seen when comparing genotypes within each sex (Supplementary Figure 1g-j, and Supplementary Table 1). Additional live MRI on P28 mice (Figs. 1c, d) and volumetric analysis of whole non-fixed brain tissue (Supplementary Fig. 1a-e) confirmed that cortices of *Taok2* KO mice are smaller than WT and *Taok2* Het mice. Finally, a longitudinal study on the same mice using diffusion tensor imaging (DTI) to map fiber tracks uncovered a regional delay of the development of neuronal tracks such as the corpus callosum in *Taok2* KO mice across multiple time points (Figs. 1e, f and Supplementary Fig. 1k-o).

Given the changes of cortical brain size, we asked whether alterations in cortical layering may contribute to these effects. Frontal, medial, and dorsal coronal brain sections were collected for the analysis. We immunostained for the upper cortex marker Cux-1 and the lower cortex marker Ctip2 to analyze laminar organization including thickness and density of cortical layers. Our results show that the thickness of the Ctip2 layer is not changed in *Taok2* KO cortices (Supplementary Fig. 2b). However, the medial and dorsal region of the cortex showed decreases in the thickness of the Cux-1-positive layer (Supplementary Fig. 2c, d), together with an overall reduced cortex thickness in the dorsal-caudal region of the *Taok2* KO cortices (Supplementary Fig. 2a). Importantly, density of Cux-1-positive cells is not altered in the *Taok2* KO cortex compared with WT littermates (Supplementary Fig. 2e). Detailed examination of the cellular distribution of Cux-1+ cells uncovered a redistribution of cells in KO cortices. Specifically, we found that Cux-1+ cells clustered more in the superficial portion of the upper cortical plate, especially in the medial-caudal and dorsal cortex (Supplementary Fig. 2f, g). Given that Cux-1+ cell density is not affected in KO cortices, these results suggest that neuronal migration defects might be the cellular substrate of these cytoarchitectural abnormalities. We directly examined neuronal migration in *Taok2* Het and KO mice and found defects in the migration of neurons born at embryonic day 15/16 that produce cortical layers 2/3 (data not shown).

To test if the anatomical changes relate to the neural communication defects, we examined the synchrony between oscillatory patterns of electrical activity in the prelimbic subdivision (PL) of the PFC and HC. Previous studies have shown that hippocampal–PFC connectivity is altered during anxiety, spatial learning, and memory-related tasks in rodents [67–70]. In vivo extracellular recordings of the LFP and multiple unit activity were conducted in postnatal day P8-10 mice because this is the period of maximal drive from HC to PL and is critical for prelimbic-hippocampal network maturation [58, 71] (Supplementary Fig. 3a-f). The oscillatory events were similar in the PFC and slightly decreased in the HC of *Taok2* KO mice; however, the duration, amplitude, and power in theta (4–12 Hz), beta (12–30 Hz), and gamma (30–100 Hz) frequency ranges were significantly augmented (Supplementary Fig. 3g-n and Supplementary Table 2) [72, 73]. The coherence within the beta band was also significantly increased in *Taok2* KO mice suggesting alterations in HC and PFC connectivity, and alterations in long-range functional connectivity (Supplementary Fig. 3o, p).

**Fig. 2:**
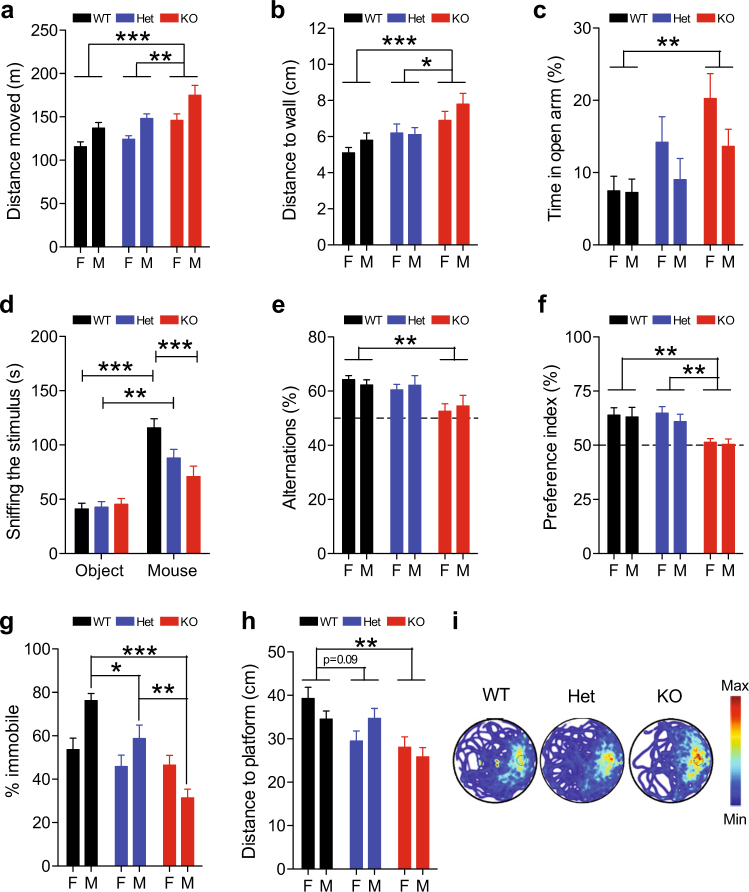
*Taok2* KO mice show alterations in neurodevelopmental disorder-related mouse behavior. **a** *Taok2* KO mice travel longer distances in the open field. (WT(M) = 12, WT(F) = 16, Het(M) = 12, Het(F) = 17, KO(M) = 9, KO(F) = 17 mice from three different cohorts; two-way ANOVA, post hoc Bonferroni’s test, effect of genotype* F*_2, 77_ = 12.89, *p* < 0.001; WT vs. KO* p* < 0.001, Het vs. KO *p* < 0.01). (**b**) *Taok2* KO mice moved at longer distances from the wall in the open field test. (WT(M) = 12, WT(F) = 16, Het(M) = 12, Het(F) = 17, KO(M) = 9, KO(F) = 16 mice from three different cohorts; two-way ANOVA, post hoc Bonferroni’s test, effect of genotype *F*_2, 76 _= 8.38, *p *< 0.001¸ WT vs. KO *p* < 0.001, Het vs. KO *p* < 0.05). **c** *Taok2* KO mice spent more time in the open arm of an elevated plus maze compared with WT. (WT(M) = 10, WT(F) = 10, Het(M) = 12, Het(F) = 12, KO(M) = 9, KO(F) = 9 mice from three different cohorts; two-way ANOVA, post hoc Bonferroni’s test; effect of genotype *F*_2, 56 _= 5.16, *p* = 0.009; WT vs. KO *p* < 0.01). **d** *Taok2* KO mice spent less time investigating and sniffing an unfamiliar sex-matched mouse instead of an unfamiliar object compared with WT littermates. (WT(F) = 11, WT(M) = 11, Het(F) = 8, Het(M) = 15, KO(F) = 12, KO(M) = 12 mice from three different cohorts; three-way mixed ANOVA, post hoc Bonferroni; effect of the interaction “genotype × stimulus” *F*_2, 63_ = 4.96, *p *= 0.001; Mouse: WT vs. KO *p *< 0.001; Object vs. Mouse: WT *p* < 0.001, Het *p* < 0.01). **e** *Taok2* KO did not perform more alternations than chance and performed fewer alternations than WT littermates. (WT(M) = 11, WT(F) = 14, Het(M) = 13, Het(F) = 15, KO(M) = 8, KO(F) = 15 mice from three different cohorts; two-way ANOVA, post hoc Bonferroni’s test, effect of genotype *F*_2, 70 _= 7.26, *p* = 0.001; WT vs. KO *p* < 0.01). **f** *Taok2* KO mice have a lower preference index than WT mice for a displaced object. (WT(M) = 11, WT(F) = 11, Het(M) = 15, Het(F) = 11, KO(M) = 10, KO(F) = 12 from three different cohorts; two-way ANOVA, post hoc Bonferroni’s test, effect of genotype *F*_2, 64_ = 9.05, *p* < 0.001; WT vs. KO *p* < 0.01, Het vs. KO *p* < 0.01). **g** *Taok2* Het and KO mice spent less time immobile during recall trial for contextual fear conditioning. (WT(M) = 8, WT(F) = 9, Het(M) = 9, Het(F) = 9, KO(M) = 7, KO(F) = 9 from three different cohorts; three-way mixed ANOVA, post hoc Bonferroni; effect of the interaction “genotype × sex” *F*_2, 45_ = 7.21,* p* = 0.002; WT vs. KO *p* < 0.001, Het vs. KO* p* < 0.01, WT vs. Het *p *< 0.05). **h** *Taok2* KO showed reduced mean minimal distance to the platform during the recall trial of the water maze test. (WT(M) = 10, WT(F) = 12, Het(M) = 11, Het(F) = 12, KO(M) = 8, KO(F) = 12 mice from three different cohorts; two-way ANOVA, post hoc Bonferroni’s test, effect of genotype *F*_2, 59_ = 8.52,* p* < 0.001; WT vs. Het *p *= 0.09, WT vs. KO < 0.01). **i** Heat maps showing *Taok2* KO male mice search for platform more at former position compared with WT and Het male mice. Blue to red indicates increased probability of a mouse being present. ns > 0.05, *p < 0.05, **p < 0.01, and ***p < 0.001. Values are mean ± s.e.m.

Next, we performed behavioral testing on *Taok2* Het and KO mice. We assessed novelty-induced exploration and anxiety in the open field test, where *Taok2* KO mice traveled longer distances (effect of genotype: *F*_2, 77_ = 12.89; *p* < 0.001) and further away from the walls (effect of genotype: *F*_2, 77_ = 8.38; *p *< 0.001, Figs 2a, b) during the 30-min trial, with increased time spent in the center and decreased time spent in the border (Supplementary Fig. 4c, d). The analyses of 5-min time bins revealed that locomotion of *Taok2* KO was enhanced only at time points 15–30 min compared with WT mice (effect of the interaction “genotype × time bin”: *F*_10, 385_ = 2.91; p = 0.002, Supplementary Fig. 4a,b), suggesting that the enhanced locomotion of *Taok2* KO mice was due to impaired short-term habituation. We further analyzed anxiety-related behavior in the elevated plus maze test. *Taok2* KO mice showed a significant increase in the time spent in the open arms of the elevated plus maze (effect of genotype* F*_2, 56_ = 5.16; *p* = 0.009, Fig. 2c), with no differences in the number of entries or time spent in the closed arm or time spent in the center (Supplementary Fig. 4e-g). Social behavior was assessed in a social preference paradigm: We found *Taok2* KO mice spent less time sniffing an unfamiliar sex-matched mouse instead of an unfamiliar object and compared with the WT littermates indicating reduced social drive (effect of the interaction genotype × stimulus: *F*_2, 63_ = 4.96; *p* = 0.001, Fig. 2d and Supplementary Fig. 5a, b). No difference in distance moved was detected in the social preference test (Supplementary Fig. 5c). Working memory was assessed in the Y-maze for spontaneous alternation. *Taok2* KO mice performed less alternations than WT mice (effect of genotype: *F*_2, 70_ = 7.26;* p *= 0.001; Fig. 2e). The impaired performance of the *Taok2* KO mice does not seem to be caused by their tendency to move faster than WT mice as the average transition time between arms did not differ from WT mice (Supplementary Fig. 5e) and percentage of alternations did not correlate with the average transition time at the individual level (Supplementary Fig. 5f). We also analyzed short-term spatial memory in the object recognition paradigm and found that *Taok2* KO mice showed a reduced preference for the displaced object when compared with WT mice (effect of genotype: *F*_2, 64_ = 9.05; *p* < 0.001; Fig. 2f), with no difference in the total distance moved (Supplementary Fig. 5d). Long-term storage and retrieval of emotionally relevant information was also examined using the contextual fear-conditioning paradigm. *Taok2* Het and KO male mice spent less time immobile compared with WT male littermates (effect of the interaction “genotype × sex”: *F*_2, 45_ = 7.21; *p* = 0.002; Fig. 2g), with no difference in the time spent immobile during the baseline and conditioning trial (Supplementary Fig. 5g, h) indicating that loss of Taok2 impairs consolidation or retention of emotional memories specifically in male mice. Finally, the water maze test was used to assess long-term spatial memory during a transfer trial performed 24 h after the last training session. *Taok2* Het and KO mice showed no difference in the learning curves, with no difference in total distance swum, mean velocity, and time spent at the border (Supplementary Fig. 6). Whereas all genotypes showed a preference for the target quadrant (Fig. 2i), *Taok2* KO mice searched more consistently for the platform at its former position than WT mice as indicated by the reduced mean minimal distance to platform (effect of genotype: *F*_2, 59_ = 8.52; *p* < 0.001; Fig. 2h) and enhanced time spent at the platform position and platform crossings (Supplementary Fig. 7a-c). Although this seems paradoxical, other ASD mouse models have displayed reduced times to find the hidden platform indicating enhanced spatial learning phenotypes [74, 75]. These findings suggest that loss of Taok2 results in behavior alterations related to cognition, anxiety, and social interaction.

### Taok2 KO mice display dendritic morphology and synaptic functional deficits

**Fig. 3:**
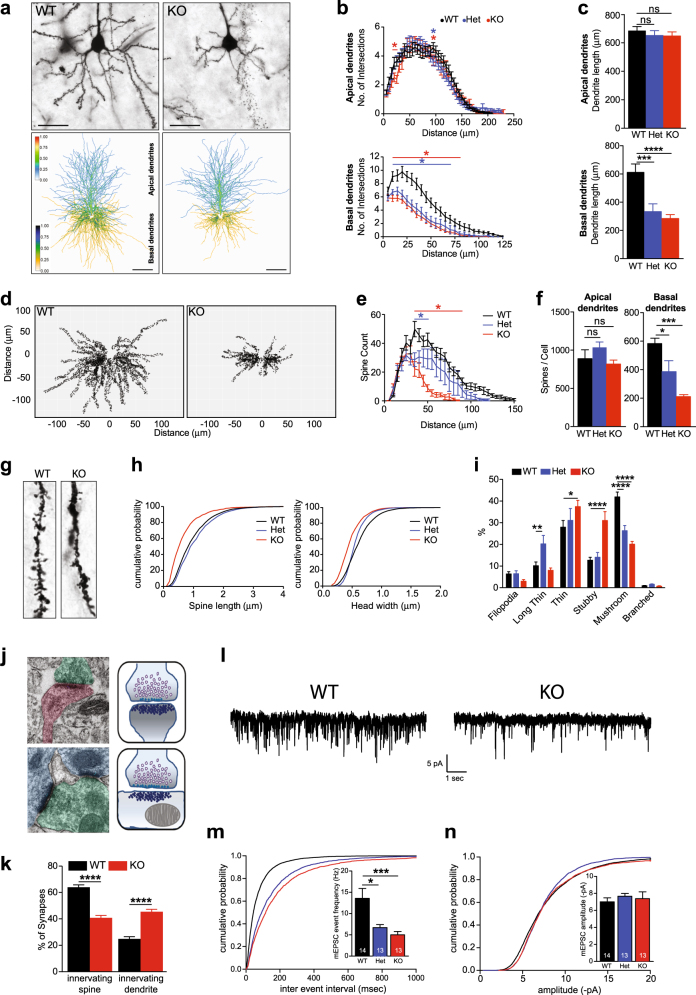
*Taok2* KO mice have reduced dendrite growth and synaptic connectivity in the prefrontal cortex. **a** Top: Golgi-stained PFC neurons from P21 WT, *Taok2* Het and KO mice. Scale bars represent 20 μm. Bottom: dendritic heat maps of superimposed neuron tracings for each condition. Blue to red (apical) and yellow to blue (basal) indicates increased probability of dendrite presence. Scale bars represent 30 μm. **b** Top: no major difference in apical dendritic complexity in layer 2 PFC neurons in *Taok2* Het and KO mice (WT = 26, Het = 32, KO = 21 neurons from three different brains; two-way ANOVA, post hoc Dunnett’s test; *F*_2, 3496_ = 3.055, *p* = 0.0472 between genotypes; *represents ranges of significance; WT vs. Het (blue), WT vs. KO (red); see supplemental statistics). Bottom: significantly reduced basal dendritic complexity in layer 2 PFC neurons in *Taok2* Het and KO mice (WT = 19, Het = 18 and KO = 18 neurons from three different brains; two-way ANOVA, post hoc Dunnett’s test; *F*_2, 1716_ = 128.7, *p* < 0.0001 between genotypes; *represents ranges of significance; WT vs. Het (blue), WT vs. KO (red); see supplemental statistics). **c** Top: no change in apical dendrite length (µm) in *Taok2* KO PFC neurons (WT = 27, Het = 34, and KO = 22 neurons from three different mice brains; one-way ANOVA, post hoc Dunnet’s test; *F*_2, 80_ = 0.3346, *p* = 0.7167). Bottom: reduced basal dendrite length (µm) in *Taok2* KO PFC neurons (WT = 22, Het = 17, and KO = 18 neurons from three different mice brains; one-way ANOVA, post hoc Dunnet’s test; *F*_2, 54_ = 12.47, *p* < 0.0001; WT vs. Het *p* = 0.0007, WT vs. KO *p* = 0.0001). **d** Dendritic spine distribution maps of Golgi-stained WT and *Taok2* KO PFC neurons. **e** *Taok2* KO PFC neurons show reduced number of distal dendritic spines (WT = 6, Het = 6, and KO = 6 neurons from three different brains; two-way ANOVA, post hoc Dunnett’s test; *F*_2, 465_ = 89.35, *p *< 0.0001 between genotypes; *represents ranges of significance; WT vs. Het (blue), WT vs. KO (red); see supplemental statistics). **f** Left: *Taok2* KO PFC neurons show no difference in total apical dendritic spines per cell (WT = 6, Het = 6, KO = 6 neurons from three different mice brains; One-way ANOVA, post hoc Dunnett’s test; *F*_2, 15_ = 1.766, *p* = 0.2048). Right: *Taok2* KO PFC neurons show decreased number of total basal dendritic spines per cell (WT = 6, Het = 6, KO = 6 neurons from three different mice brains; One-way ANOVA, post hoc Dunnett’s test; *F*_2, 15_ = 13.76, *p* = 0.0004; WT vs. Het *p* = 0.0265, WT vs. KO *p* = 0.0002. **g** Images of dendritic spines on P21 WT and *Taok2* KO PFC neuron dendrites. **h** Cumulative probability histograms show shift toward reduced dendritic spine lengths (left) *Taok2* KO PFC neurons and reduced head widths (right) in *Taok2* Het and KO PFC neurons. **i** *Taok2* KO PFC neurons have a significant increase in thin and stubby shaped spines and reduction in mushroom-like spines compared with WT PFC neurons, whereas *Taok2* Het PFC neurons have an increase in long thin shaped spines and reduction in mushroom-like spines (WT = 3504, Het = 2317, KO = 1262 spines from six cells per condition from three different brains; two-way ANOVA, post hoc Dunnett’s test; *F*_2, 90_ = 0.0008294,* p* = 0.9992 between genotypes; WT vs. Het filopodia:* p* = 0.9997, long thin: *p* = 0.0088, thin:* p* = 0.5599, stubby: *p* = 0.8934, mushroom: *p* < 0.0001, and branched: *p* = 0.9765; WT vs. KO filopodia: *p* = 0.5111, long thin: *p* = 0.7565, thin:* p* = 0.0141, stubby:* p* < 0.0001, mushroom:* p* < 0.0001, and branched: *p* = 0.9943). **j** Representative images of synapses innervating spines (top) and synapses innervating dendrites (bottom) and illustrations of each (right). **k** *Taok2* KO neurons imaged by electron microscopy show decreased percentage of synapses on postsynaptic spines (left) and increased synapses on dendrites (right) (WT = 159 and KO = 120 neurons; innervating spine: unpaired *t-*test; t(277) = 8.814, *p *< 0.0001; innervating dendrite: t(277) = 8.262, *p *< 0.0001). **l** Representative traces of mEPSC spikes from WT and *Taok2* KO PFC neurons. Scale: 5pA vs. 1 s. **m** Longer inter-event intervals in *Taok2* Het and KO PFC neurons shown on a cumulative probability histogram. Inside: reduced mean mEPSC event frequency in *Taok2* Het and KO PFC neurons (WT = 14, Het = 13 and KO = 13 neurons from three different mice brains; Kruskal-Walis ANOVA, post hoc Dunn’s test; H-value = 14.47,* p* = 0.0007; WT vs. Het *p* = 0.0212, WT vs. KO *p* = 0.0008). **n** No change in mEPSC amplitude in *Taok2* WT, Het and KO PFC neurons shown on a cumulative probability histogram. Inside: mean mEPSC amplitude of *Taok2* WT, Het and KO neurons (WT = 14, Het = 13 and KO = 13 neurons from three different mice brains; kruskal-walis ANOVA, post hoc Dunn’s test; H-value = 3.871, *p *= 0.1444; WT vs. Het *p* = 0.1857, WT vs. KO* p* > 0.9999). *p < 0.05, **p < 0.01, ***p < 0.001 and ****p < 0.0001. Values are mean ± s.e.m.

To complement the behavioral and in vivo electrophysiology experiments, we assessed neural morphology in vivo using Golgi-Cox staining on 3-week-old WT and *Taok2* Het and KO brains. Analysis of prefrontal *Taok2* Het and KO neurons revealed a significantly reduced basal dendrite complexity and length compared with WT neurons, with only minor reductions in the apical dendrites (Figs. 3a-c). Examination of somatosensory neurons showed a small and partially significant difference in dendritic arborization in *Taok2* KO and WT cells (Supplementary Fig. 8a-c), but not in hippocampal neurons (Supplementary Fig. 9a-c).

Next, we analyzed whether Taok2 regulates synapse development in vivo and found that *Taok2* KO neurons had significant changes in spine distribution (Figs. 3d, e) and a reduction in the total number of basal dendrite spines per PFC neuron (Fig. 3f) but not in the somatosensory cortex or the HC (Supplementary Fig. 8d-f and Supplementary Fig. 9d-f). Furthermore, all three regions showed no difference in the number of apical dendrite spines (Fig. 3f, Supplementary Fig. 8f and Supplementary Fig. 9f). *Taok2* Het neurons showed a gene dosage-dependent difference in spine distribution and significant reduction in total basal dendrite spine numbers in PFC neurons only (Figs. 3e-f). In addition, our semi-automatized analysis of spine morphology [64] revealed shorter spine length and head width in *Taok2* KO neurons and shorter head width in *Taok2* Het neurons (Fig. 3h). Concurrently, the number of thin and stubby (immature) spines significantly increased concomitantly with a reduction in mushroom-shaped (mature) spines in *Taok2* KO neurons (Figs. 3g, i). Interestingly, *Taok2* Het neurons showed a significant increase in long thin (immature spines) and decrease in mushroom-shaped spines, but no changes in stubby spines (Fig. 3i). This phenotype was also detected in the somatosensory cortex (Supplementary Fig. 8g, h) showing an increase in thin spines, but not in the HC (Supplementary Fig. 9g, h). Electron microscopy analysis of *Taok2* KO brains revealed that the number of synapses formed onto the dendrite shaft instead of the postsynaptic spine heads was increased in prefrontal and somatosensory cortical neurons (Figs. 3j, k and Supplementary Fig. 8i), but not in the HC (Supplementary Fig. 9i). Supporting these findings, we found that the level of phosphorylated Taok2 is higher in the cortex compared with the HC (Supplementary Fig. 9j, k). Together our findings indicate that Taok2 is a key regulator of synapse formation predominantly in the PFC, and also the somatosensory cortex in vivo.

Next, we tested synaptic transmission using in situ electrophysiology. We performed whole-cell patch-clamp recordings of acute brain slices (P21–28) and found a significant reduction in the mean frequency of miniature excitatory postsynaptic currents (mEPSCs), but not the mean amplitude, in the PFC (Fig. 3l-n) and the somatosensory cortex (Supplementary Fig. 8j-k). *Taok2* Het brain slices also showed a strong reduction in the frequency of mEPSCs (Fig. 3m and Supplementary Fig. 8k). We also measured miniature inhibitory postsynaptic currents but found no differences (Supplementary Fig. 10). These data indicate that *Taok2 Het* and KO mice display multiple abnormalities in neuronal morphology and synaptic function in cortical excitatory neurons.

### Taok2 is present in the postsynaptic density and regulates synapse formation in vitro

We also assessed whether Taok2 is expressed at the postsynaptic density (PSD). We observed that Taok2 colocalizes with the postsynaptic protein SynGAP (Supplementary Fig. 11a) and, when isolated in synaptosomes from P28 mouse brains, we found both, phosphorylated and non-phosphorylated Taok2 in the PSD95-positive fraction (Supplementary Fig. 11b). We also examined synapse formation in vitro using WT and *Taok2* KO cortical neuron cultures and determined that *Taok2* KO neurons display a significant reduction in spines and a reduction in synaptic SynGAP-positive puncta that colocalize with phalloidin-stained (F-actin enriched) dendritic spines (Supplementary Fig. 11c-e). Acute downregulation of *Taok2* using short hairpin RNA (shRNA) in WT cultured cortical neurons further showed a dosage-dependent decrease in dendritic spine density (Supplementary Fig. 11f, g) and an increase in mobility of dendritic spines by tracking farnesylated (membrane-bound)-green fluorescent protein (GFP)-stained spines and Lifeact-GFP stained actin-enriched protrusions (Supplementary Fig. 11h, i). These in vitro data indicate that TAOK2 regulates spine and synaptic deficits in a cell-autonomous manner by affecting actin dynamics and stability.

### Identification of de novo and LOF mutations in TAOK2 from ASD cohorts via whole-genome sequencing

**Fig. 4:**
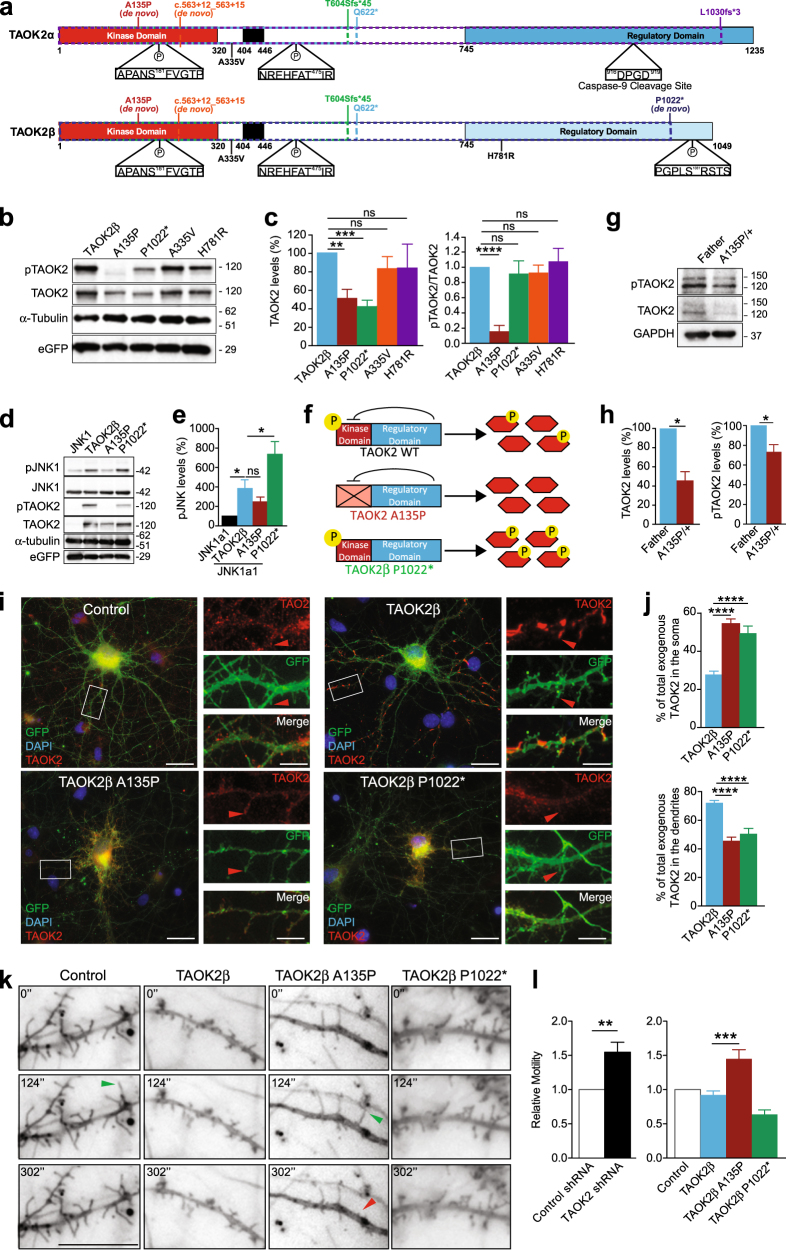
De novo mutations in TAOK2 impair phosphorylation at Ser181, localization in cortical neurons, and dendritic spine motility. **a** Diagram of TAOK2α and TAOK2β isoforms and location of *de novo* (A135P, P1022* and 563 + 12_563 + 15), truncating mutations (T604Sfs*45, Q622*, L1030fs*3), and rare-inherited variants (A335V and H781R). Different protein domains are represented by colored boxes (kinase domain: red, MEK binding domain: black, regulatory domains: blue (α) and light blue (β)). TAOK2α has two phosphorylation sites (ser181 and thr475) and caspase-9 cleavage site (^916^DPGD^919^). TAOK2β has three known phosphorylation sites (ser181, thr475, and ser1031). **b** Western blot of HEK293 cell lysates 48 h post transfection with TAOK2β and β variants (A135P, P1022*, A335V, and H781R). **c** TAOK2β A135P shows reduced protein expression and ser181 phosphorylation, TAOK2β P1022* shows only reduced expression, and TAOK2β A335V and H781R have no effect compared with TAOK2β (*n* = 6–8 western blots; one-sample *t*-test; TAOK2 levels: A135P t(5) = 5.303, *p *= 0.0032; P1022* t(5) = 8.903, *p* = 0.0003; A335V t(5) = 1.342,* p* = 0.2373, H781R* t(5) = 0.6381, *p* = 5515; pTAOK2 levels: A135P t(7) = 10.93,* p* < 0.0001; P1022* t(7) = 0.4992,* p* = 0.6330; A335V t(7) = 0.7243, *p *= 0.4924; H781R t(7) = 0.3526, *p* = 0.7348). **d** Western blot of HEK293 cell lysates 48 h post transfection with JNK1a1 only or with TAOK2β, TAOK2β A135P and TAOK2β P1022*. **e** TAOK2β P1022* significantly increases phosphorylation of JNK1a1 in HEK293 cells compared with TAOK2β (*n* = 7 western blots; one-way ANOVA, post hoc Dunnett’s test; *F*_2, 18_ = 6.88, *p* = 0.0060; TAOK2β vs. P1022* *p* = 0.0342, TAOK2β vs. A135P *p *= 0.5087; JNK1a1 only (set to 100%) vs. TAOK2β: one-sample *t-*test, t(6) = 3.167, *p* = 0.0194). **f** Schematic showing impairment of ser181 auto-phosphorylation by the A135P mutation resulting in reduced kinase activity on downstream targets, whereas the P1022* mutation causes increased kinase activity of TAOK2. **g** Western blot of LCLs from the A135P proband and the unaffected father. **h** The A135P proband has reduced TAOK2 and pTAOK2 levels compared with the unaffected father (*n* = 5 western blots; one-sample *t*-test; TAOK2: t(4) = 4.557,* p* = 0.0104; pTAOK2: t(4) = 2.74, *p* = 0.0519). **i** Images of DIV14 cortical neuron cultures transfected with only GFP (control) or TAOK2β, TAOK2β A135P, and TAOK2β P1022* with GFP and immunostained against GFP (green), TAOK2 (red) and stained with DAPI (blue). Scale bars represent 10 μm. Boxes are shown magnified (right), with scale bars representing 3 μm. Arrowheads represent dendritic spines filled with exogenous TAOK2β, but not TAOK2β A135P and P1022*. **j** Percentage of total exogenous TAOK2β A135P and P1022* is increased in the soma (top) and decreased in the dendrite and spines (bottom) compared with TAOK2β (TAOK2β = 47, TAOK2β A135P = 35 and TAOK2β P1022* = 40 neurons from three separate cultures; one-way ANOVA post hoc Dunnett’s test; Soma *F*_2, 119_ = 28.01, *p* < 0.0001: TAOK2β vs. A135P *p* < 0.0001, TAOK2β vs. P1022* p < 0.0001; Dendrite: *F*_2, 119 _= 28.01: TAOK2β vs. A135P *p* < 0.0001, TAOK2β vs. P1022* *p* < 0.0001). **k** Snapshots of DIV14 cortical neurons transfected with TAOK2β and β variants (A135P and P1022*) at 0 s, 124 s and 302 s. Green arrowheads indicate extending filopodia spines and red arrowhead indicates retraction of a filopodia spine. **l** Left: increased spine motility in neurons transfected with *Taok2* shRNA compared with neurons transfected with control shRNA (Control shRNA = 12 and *Taok2* shRNA = 18 neurons from three different cultures; one-sample *t*-test; t(17) = 3.799, *p* = 0.0014). Right: TAOK2β A135P transfected neurons have increased spine motility compared TAOK2β transfected neurons (Control = 10, TAOK2β = 22, A135P = 12 and P1022* = 14 neurons from three different cultures; one-way ANOVA, post hoc Dunnett’s test; *F*_3, 42 _= 17.2,* p* < 0.0001; TAOK2β vs. A135P* p* = 0.0002).*p < 0.05, **p < 0.01, ***p < 0.001 and ****p < 0.0001. Values are mean ±  s.e.m.

Analysis of *TAOK2* from the Exome Aggregation Consortium (ExAC) reveals it is highly constrained for LOF mutations (pLi = 1; the highest possible score) and missense mutations (z = 3.54) [76], suggesting that mutations in *TAOK2* could have deleterious effects. We used whole-genome and -exome sequencing of over 2600 families with ASD to detect de novo or inherited genetic variants [77]. We detected 24 different variants in *TAOK2* (Supplementary Table 3 and Supplementary Fig. 12a), which were confirmed by Sanger sequencing (data not shown). We focused on six genetic alterations from unrelated families (Table 1, Fig. 4a and Supplementary Fig. 12b), of which three are known to be de novo; a missense mutation in the kinase domain (A135P), a C-terminal frameshift deletion resulting in truncation (P1022*; this proband also has a frameshift mutation in *CHD8*) and a de novo splice site variant (c.563 + 12_563 + 15 del) predicted to cause intron 7 retention (Supplementary Fig. 13c). These de novo mutations in *TAOK2* are not present in the ExAC database [20]. We also identified three additional protein-truncating variants in *TAOK2* (Table 1 and Supplementary Fig. 12a), where one is a rare-inherited variant and two have unknown genetic inheritance. All six probands have been diagnosed clinically with autism with their diagnostic test scores summarized in Table 1. Given the importance of *de novo* mutations, they were functionally assessed. Droplet digital PCR confirmed that the mutations are germline (data not shown). We then tested the gene expression of *TAOK2* in lymphoblastoid cell lines (LCLs) of patients and found no significant changes for all isoforms (Supplementary Fig. 13a, b).

**Table. 1:** Diagnostic score summary of individuals diagnosed with ASD that have de novo or truncating variants in *TAOK2* ▓

Proband	Variant	Type	RefSeq effect	Affected isoforms	Inheritance	Polyphen score	Diagnosis	IQ	Language	Adaptive behavior	Other medical info
1-0337-003	*TAO2 A135P*	HET	MISSENSE	Alpha Beta Gamma	De novo	0.97	ADI dx: autism ADOS dx: not autism spectrum	Leiter-R: 123	OWLS: listening comprehension SS = 81, oral expression SS = 77, oral composite SS = 77	VABS-II: communication SS = 79, daily living SS = 75 ,socialization SS = 72 ,adaptive behavior SS = 74	
1-0559-003	TAO2 P1022*	HET	FRAMESHIFT DELETION	Beta	De novo	N/A	ADI and ADOS dx: autism; SCQ dx: autism	WASI-II: verbal comprehension composite = 55, perceptual reasoning composite = 71	OWLS: listening comprehension SS = 40, oral expression SS = 37, oral composite SS = 40	VABS-II: communication SS = 70, daily living SS = 66, socialization SS = 61, adaptive behavior SS = 64; ABAS-II: GAC composite = 40, practical composite = 40, social composite = 54, conceptual composite = 49	CHD8 mutation (T2050Nfs*17)
7-0179-003	*TAO2 c.563 + 12_563 + 15*	HET	SPLICE SITE DELETION	Alpha Beta Gamma	De novo	N/A	SCQ dx: autism			ABAS-II: GAC composite = 69, practical composite = 68, social composite = 72, conceptual composite = 84	ADHD-Inattentive (SWAN)
1-0446-003	TAO2 L1030Wfs*3	HET	FRAMESHIFT DELETION	Alpha Beta Gamma	N/A	N/A	ADOS dx: autism	WASI-II: verbal IQ = 117, performance IQ = 84, full scale IQ = 100	OWLS: listening comprehension SS = 96, oral expression SS = 91, oral composite SS = 92; PPVT-4: SS = 99	VABS-II: communication SS = 74, daily living SS = 66, socialization SS = 61, adaptive behavior SS = 65	
AU4112303	TAO2 T604Sfs*45	HET	NONSENSE	Alpha Beta Gamma	N/A	N/A	Clinical dx: autism; ADI dx: autism		PPVT-4: SS = 56	SRS-2 T-score (latest): social communication = 56, social motivation = 56, social awareness = 50, social cognition = 61, autistic RRb/mannerisms = 58 total score = 57	
AU4261301	TAO2 Q622*	HET	FRAMESHIFT DELETION	Alpha Gamma	Paternal; Present in unaffected and affected sibling	N/A		N/A	N/A	N/A	N/A

Next, we tested whether intron 7 was retained on the de novo splice site mutation (c.563 + 12_563 + 15 del). We found a low-level retention (approximately 1%) in all family members and unrelated wild-type *TAOK2* individuals. Interestingly, this proband showed a significantly higher level of intron retention (approximately 13%), which introduced a premature stop codon (Supplementary Fig. 13c). Although the impact of this remains unknown and requires further testing, it indicates the splice site mutation is detrimental to normal TAOK2 splicing.

### De novo mutations in TAOK2 alter kinase activity, protein stability, neuronal localization, and dendritic spine motility

We functionally assessed the de novo mutations and two rare-inherited variants and tested whether they impaired auto-phosphorylation at serine 181 in both α and β isoforms [44]. We studied the A135P mutation (present in both isoforms) and the P1022* mutation (present only in the β isoform) along with the A335V and H781R rare-inherited mutations (present in both or in the β isoform only, respectively) (Fig. 4a). We expressed WT TAOK2α, TAOK2β and the respective mutants in HEK293 cells and analyzed TAOK2 phosphorylation and expression. We found a striking reduction in TAOK2 auto-phosphorylation caused by the A135P mutation in both isoforms, identifying this as a kinase dead mutation (Figs. 4b, c and Supplementary Fig. 14a, b), whereas the P1022* mutation did not alter auto-phosphorylation (Figs. 4b,c). Interestingly, both mutations, A135P and P1022*, reduce protein expression of TAOK2 suggesting impaired protein stability (Figs. 4b, c). The A335V and H781R rare-inherited variants did not alter protein expression or auto-phosphorylation, suggesting that they have minimal impact on protein function and expression (Figs. 4b, c and Supplementary Fig. 14a, b). Therefore, further experiments were done using only the de novo mutations. Next, we analyzed the effect of these mutations on one of the known downstream targets of TAOK2, JNK1 [45, 78]. TAOK2α phosphorylates JNK1 more than the β isoform, and accordingly we found that TAOK2α bearing the A135P mutation significantly decreased JNK1 phosphorylation, whereas the A135P mutation in the β isoform showed no significant effect (Figs. 4d, e and Supplementary Fig. 14c, d). The P1022* mutation (C-terminal deletion), however, significantly increased phosphorylation of JNK1 compared with WT TAOK2 (Figs. 4d, e). This is consistent with previous reports identifying the C-terminus of TAOK2 as a negative regulator of TAOK2 kinase activity, predicting dysregulated (overactive) kinase activity [78, 79]. In summary, the A135P mutation impairs auto-phosphorylation and TAOK2 kinase function (LOF, analogous to the TAOK2 KO), whereas the P1022 mutation enhances TAOK2 kinase function (gain-of-function (GOF), analogous to an overexpression of TAOK2 (Fig. 4f).

Finally, we assessed the impact of the mutations in patient-derived LCLs, which endogenously express the mutation. We found that the proband harboring the A135P mutation had significantly reduced pTAOK2 and TAOK2 protein levels compared with LCLs from their unaffected father, which is consistent with our HEK293 cell data (Figs. 4g, h). Consistently, the P1022* mutation caused no change on pTAOK2, but no difference in TAOK2 expression was observed, possibly due to the heterozygosity of the patient (Supplementary Fig. 15a, b). Our data indicate that patient-derived de novo mutations in TAOK2 significantly impact TAOK2 function by decreasing (LOF) or increasing (GOF) intrinsic kinase activity toward downstream targets (Fig. 4f).

We next analyzed whether the TAOK2 mutations impact cellular distribution in primary neurons. We expressed WT and mutated α and β isoforms of TAOK2 in cortical neurons and analysis of GFP co-transfected DIV14 cortical neurons showed that WT TAOK2α is primarily localized to the dendritic shaft (co-stained with tubulin), but not present in actin-rich dendritic protrusions (co-stained with rhodamine-labeled phalloidin) (Supplementary Fig. 14e). Additionally, the A135P mutation does not affect TAOK2α localization (Supplementary Fig. 14f). On the other hand, we observed that WT TAOK2β is expressed in dendrites with strong accumulation in filopodia/spine protrusions (Fig. 4i). We found that both mutations, A135P and P1022*, significantly increased the proportion of exogenous TAOK2 in the soma while concurrently reducing exogenous TAOK2β in dendrites, specifically in filopodia/spines (Figs. 4i, j). These results suggest that the mutations impact protein trafficking of TAOK2β in dendrites and spines.

Next, we measured dendritic spine motility at DIV14 in vitro. As a measure of spine dynamics, we analyzed the distance moved by the center of mass (CoM) of individual spines over 5 min [65]. We first tested this system by analyzing neurons transfected with either control or TAOK2 shRNA and found that acute silencing of TAOK2 caused a significant increase in filopodia/spine motility [48] (Fig. 4l, Supplementary Fig. 11h, i and Supplementary Video 1). Interestingly, we observed a comparable increase in filopodia/spine motility when the TAOK2β A135P mutation was expressed in neurons, whereas the TAOK2β P1022* mutation shows no significant effect or a trend toward decreased motility (Figs. 4k, l and Supplementary Video 2). The lack of change in spine motility when overexpressing TAOK2β or an overactive TAOK2β (P1022* mutant) indicates that A135P is the dominant LOF mutation that alters filopodia motility resembling the *Taok2* KO condition.

### De novo mutations in TAOK2 impair dendrite and synaptic development

**Fig. 5:**
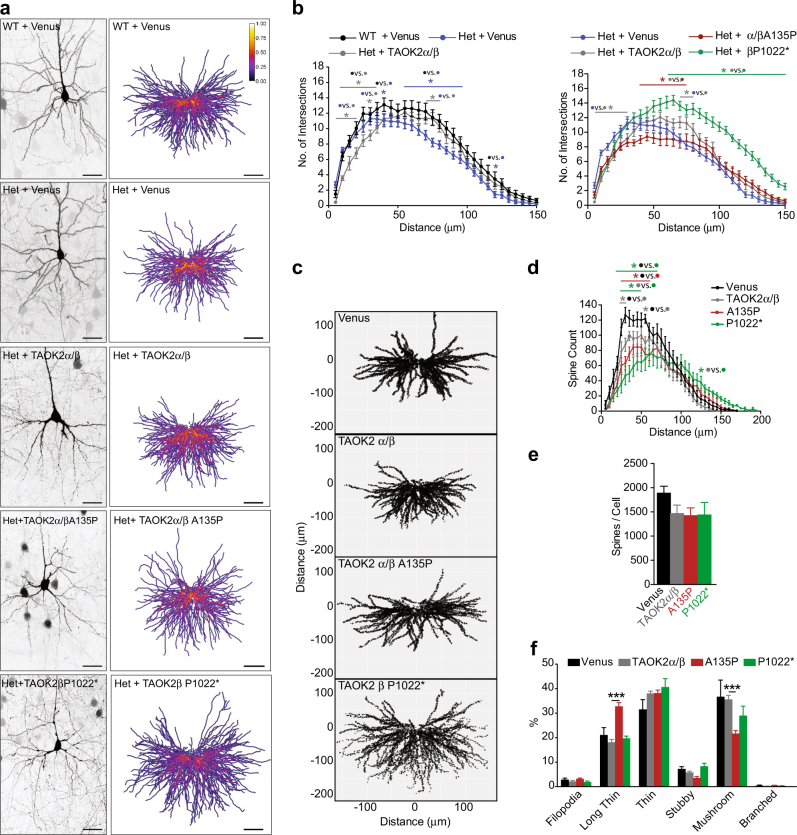
De novo mutations in TAOK2 impair dendrite growth and synaptic connectivity in the mouse cortex. **a** Left: representative images of WT or Het *Taok2* cortical neurons from P21 mice in utero electroporated at E15 with GFP only (Venus, control) or Het *Taok2* cortical neurons with TAOK2α/β, TAOK2α/β A135P, and TAOK2β P1022*. Scale bars represent 20 μm. Right: dendritic heat maps of superimposed neuron tracings for each condition. Blue to yellow indicates increased probability of dendrite presence. Scale bars represent 40 μm. (**b**) Left: Het *Taok2* neurons show reduced dendritic complexity that can be partially rescued with overexpression of TAOK2α/β in the Het background (WT + Venus = 20 cells, Het + Venus = 18 cells, Het + TAOK2α/β = 16 cells from three different brains; two-way ANOVA, post hoc Tukey’s test; *F*_2, 1989_ = 42.48, *p* < 0.0001 between genotypes; *represents ranges of significance; WT + Venus vs. Het + Venus (blue), WT + Venus vs. Het + TAOK2α/β (gray), Het + Venus vs. Het + TAOK2α/β (gray); see supplemental statistics). Right: TAOK2α/β A135P reduces proximal dendritic complexity and TAOK2β P1022* enhances distal dendritic complexity compared with TAOK2α/β (Het + Venus = 20 cells, Het + TAOK2α/β = 16 cells, Het + TAOK2α/β A135P = 20 cells and Het + TAOK2β P1022 = 16 cells from three different brains; two-way ANOVA, post hoc Dunnett’s test; *F*_3, 2720 _= 184.1, *p* < 0.0001 between genotypes; *represents ranges of significance; TAOK2α/β vs. Venus (gray), TAOK2α/β vs. A135P (red), TAOK2α/β vs. P1022* (green); see supplemental statistics). **c** Dendritic spine distribution map of cortical neurons from WT *Taok2* cortical neurons from P21 mice in utero electroporated at E15 **d** TAOK2α/β A135P and TAOK2β P1022* reduce the number of spines on proximal dendrites, whereas TAOK2β P1022* also shifts spines to distal dendrites (Venus = 11414, TAOK2α/β = 8859, α/β A135P = 9071 and β P1022* = 8686 spines from six neurons from three different brains per condition; two-way ANOVA, post hoc Tukey’s test; *F*_3, 780_ = 19.58, *p* < 0.0001 between genotypes; *represents ranges of significance; Venus vs. TAOK2α/β (gray), T AOK2α/β vs. β P1022*(green), Venus vs. α/β A135P (red), Venus vs. β P1022*(green); see supplemental statistics). (**e**) TAOK2α/β A135P and TAOK2β P1022* show no significant change in dendritic spines per neuron (Venus = 11,414, TAOK2α/β = 8859, α/β A135P = 9071 and β P1022* = 8686 spines from six neurons from three different brains per condition; spine number: one-way ANOVA, post hoc Dunnett’s; *F*_3, 20 _= 1.539, *p* = 0.2354). **f** TAOK2α/β A135P significantly increases long thin spines and reduces mushroom-like spines in *Taok2* Het neurons (Venus = 11,414, TAOK2α/β = 8859, α/β A135P = 9071 and β P1022* = 8686 spines from six neurons from three different brains per condition; two-way ANOVA, post hoc Dunnett’s test; *F*_3,__120_ = 4.36e-13, *p *> 0.9999; Long Thin: TAOK2α/β vs. TAOK2 α/β A135P *p *= 0.0001; Mushroom: TAOK2α/β vs. TAOK2 α/β A135P *p *= 0.0003). *p < 0.05, ***p < 0.001. Values are mean ± s.e.m.

We next examined the effect of the de novo mutations on neurons in vivo, using in utero electroporation at E15 to transfect neural progenitor cells that produce layer 2/3 somatosensory cortical excitatory neurons. Our analysis of P21 WT and *Taok2* Het mouse cortices revealed a significant reduction in dendritic complexity with the loss of one *Taok2* allele (Figs. 5a, b). Introduction of WT TAOK2α/β in the *Taok2* Het background using in utero electroporation significantly rescued the reduction in dendritic arborization; however, it did not completely match WT mice (Figs. 5a, b). To examine the de novo mutations, we expressed either Venus-GFP alone or together with WT TAOK2α/β, TAOK2α/β A135P (the patient possesses the mutation in both isoforms), or TAOK2β P1022* (the patient only expresses the mutation in the β-isoform) in *Taok2* Het mice. We found that expression of the TAOK2α/β A135P mutation did not rescue the dendrite phenotype of *Taok2* Het mice and revealed reduced branching (Figs. 5a, b) indicating potential dominant-negative effects. Interestingly, the TAOK2β P1022* mutation significantly enhanced distal dendrite branching (Figs. 5a, b) with elongated dendrites (Supplementary Fig. 16a) compared with WT TAOK2α/β. Although the P1022* mutation reduces the level of TAOK2 expression, it also makes it highly active toward JNK1. This is consistent with our observation that the P1022* mutations elevates phosphorylation of pJNK1 (Figs. 4d, e) and previous work has shown that active JNK1 (pJNK1) increases cortical neuron dendrite branching [45]. We further examined dendrite integrity by measuring the thickness of the dendrite after a branching point. We found that while WT TAOK2α/β increases branch thickness, the TAOK2α/β A135P and TAOK2β P1022* mutations had significantly reduced thickness (Supplementary Fig. 16b, c). These data demonstrate the complex effects of the de novo mutations on dendrite formation.

Finally, we analyzed the effects of the TAOK2 mutations on dendritic spine density and maturation. We found that TAOK2α/β A135P and TAOK2β P1022* both decreased proximal dendritic spines (Figs. 5c, d) without changing the total number of spines per neurons (Fig. 5e). Unexpectedly, TAOK2α/β also decreased the number of proximal dendritic spines (Fig. 5d). However, as the 16p11.2 copy number variation (CNV) duplication is associated with schizophrenia, this suggests that elevated expression of TAOK2 may also be detrimental to neuron development. Regarding spine morphology, only overexpression of TAOK2α/β A135P significantly increased the number of immature long thin spines while decreasing the number of mature, mushroom-shaped spines (Fig. 5f). This again indicates dominant negative effect (LOF) of the kinase dead TAOK2α/β A135P mutation, whereas TAOK2α/β and TAOK2β P1022* mutations had no effect on dendritic spine morphology (Fig. 5f). Taken together, our results show that the human-derived mutations in TAOK2 differentially impair normal dendrite and spine development.

### Taok2 regulates spine function through RhoA signaling

**Fig. 6:**
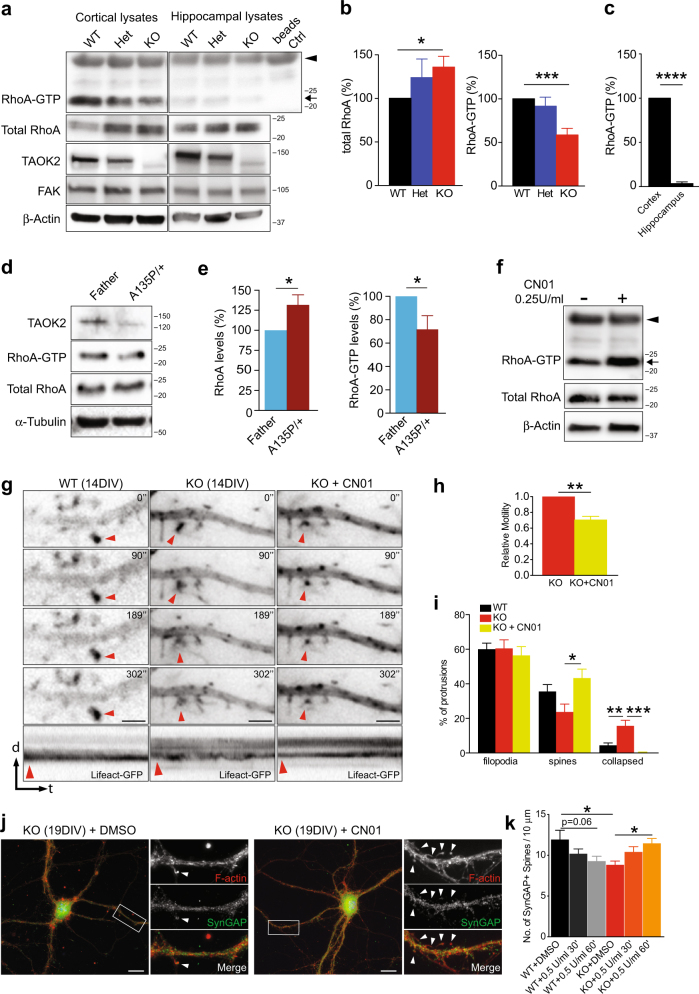
TAOK2 regulates spine function through RhoA signaling. **a** Western blot of RhoA-GTP (arrow) and total RhoA in cortical and hippocampal lysates from P21 WT, Het, and KO *Taok2* mice. Arrowhead indicates background from Rhotekin-GST. **b** Increased levels of total RhoA and reduced levels of Rho-GTP (normalized to β-actin) in the *Taok2* KO mice cortex. **c** Reduced levels of RhoA-GTP (normalized to β-actin) in the hippocampus compared with the cortex in WT mice. (*n* = 4 western blots; one-sample* t*-test; Hippocampus: t(3) = 46.03, *p* < 0.0001). (Eight separate mouse cortices per condition; total RhoA: one-sample *t-*test; WT vs. KO: t(7) = 2.905, *p* = 0.0228; RhoA-GTP: one-sample *t*-test; WT vs. KO: t(7) = 5.55,* p* = 0.0009). **d** Western blot of RhoA-GTP and total RhoA in LCLs of the A135P proband and the unaffected father. **e** Increased levels of total RhoA and reduced levels of Rho-GTP in LCLs of the A135P proband compared with the unaffected father (nine separate lysates per LCL; RhoA levels (normalized to tubulin): one-sample *t-*test; t(80) = 2.499, *p *= 0.0370; RhoA-GTP: one-sample *t-*test; t(8) = 2.399, *p* = 0.0433). **f** Western blots of the SHSY-5Y neuroblastoma cell lines showing increased RhoA-GTP levels after addition of the RhoA activator (CN01) (arrow). Arrowhead indicates background from Rhotekin-GST. **g** Snapshots from time-lapse analysis (0, 90, 189, and 302 s) of dendritic filopodia/spines from DIV14 cortical neurons labeled with Lifeact-GFP from WT and KO *Taok2* mice and *Taok2* KO treated with CN01 (1 U/ml). Red arrowheads indicate actin-rich protrusions selected for kymograph analysis. Bottom: kymographs reveal diffuse and unstable spine movement in *Taok2* KO neurons that is rescued by CN01. Scale bars represent 3 µm. **h** CN01 reduces spine motility of *Taok2* KO neurons during a 5-min period (KO = 105, KO + CN01 = 109 spines from 9 cells per condition; Wilcoxon-matched paired *t*-test, p = 0.0039). **i** CN01 increased percentage of stable spines and reduced the percentage of collapsed spines in *Taok2* KO neurons during a 5-min period (WT = 418 spines from 8 cells, KO = 603 spines from 9 cells and KO + CN01 = 433 spines from 7 cells; Filopodia: one-way ANOVA, post hoc Tukey’s test; *F*_2, 21 _= 0.2249, *p* = 0.8005; Spines: one-way ANOVA, post hoc Tukey’s test; *F*_2, 21_= 4.66, *p* = 0.0211; WT vs. KO + CN01 *p* = 0.4905, KO vs. KO + CN01 *p *= 0.0180; Collapsed: one-way ANOVA, post hoc Tukey’s test; *F*_2, 21 _= 12.5, *p *= 0.0003; WT vs. KO *p *= 0.0047, WT vs. KO + CN01 *p *= 0.4473, KO vs. KO + CN01 *p *= 0.0003). **j** Images of DIV18-19 *Taok2* KO neurons with or without addition of CN01 for 30–60 min, stained with phalloidin and SynGAP. Scale bars represent 10 µm. **k** CN01 increases the number of SynGAP + spines in *Taok2* KO neurons compared with DMSO control KO neurons (WT + DMSO = 6 cells, WT + CN01 (0.5U/ml/30′) = 8 cells, WT + CN01(0.5U/ml/60′) = 8 cells, KO + DMSO = 9 cells, KO + CN01 (0.5U/ml/30’) = 9 cells; KO + CN01 (0.5U/ml/60’) = 9 cells; one-way ANOVA, post hoc Bonferroni’s test; *F*_5, 43 _= 3.192, *p* = 0.0154; WT + DMSO vs. KO + DMSO *p* = 0.0156, WT + DMSO vs. WT + CN01(0.5U/ml/60’) *p* = 0.0611, KO + DMSO vs. KO + CN01(0.5U/ml/60′) *p* = 0.0235). *p < 0.05, **p < 0.01, and ***p < 0.001. Values are mean ± s.e.m.

To understand how TAOK2 regulates spine formation beyond previously identified pathways, we asked if regulation of the actin cytoskeleton was involved given that it associates with actin-regulating proteins [47, 48]. Furthermore, the 16p11.2 CNV, which harbors *TAOK2*, was recently associated with abnormal RhoA signaling, a regulator of F-actin stability [11, 13, 80]. Therefore, we asked whether RhoA levels and activity are changed in *Taok2* KO brains. We isolated active (GTP-bound) RhoA from cortical and hippocampal KO brain lysates and observed a decrease in RhoA activity in cortical lysates compared with WT. Concurrently, we found an increase in total RhoA protein in the lysates of *Taok2* KO cortices (Figs. 6a, b). In the HC, however, the levels of activated RhoA were only marginally affected by the lack of Taok2. However, when we compared the overall levels of RhoA activity in cortices and hippocampi in *Taok2* WT mice, we observed a striking decline in the amount of active RhoA GTPase in the HC (Figs. 6a, c), matching the low expression levels of phosphorylated Taok2 in the HC (Supplementary Fig. 9j, k). This suggests that *Taok2* may regulate the RhoA pathway predominantly in the cortex, but not in the HC.

To corroborate this data, we examined human LCLs from the A135P proband and also found significantly less activated RhoA and a significant increase in RhoA levels (Figs. 6d, e). Analysis of the LCLs from the P1022* family revealed no changes in RhoA activity (Supplementary Fig. 15c, d), indicating again that the A135P and P1022* mutations have different effects, likely because they are localized in different functional domains within the TAOK2 protein.

Our data suggest that reduced RhoA activation may contribute to the dendritic spine phenotypes in the *Taok2* KO mice by reducing F-actin stability. We directly tested this by monitoring F-actin using Lifeact-GFP, which is a 17-amino-acid peptide that labels F-actin with GFP without affecting its function [81, 82]. We measured the distance moved by the CoM of the Lifeact-GFP signal in individual spines [65]. We found that the F-actin signal was diffuse and unstable in filopodia/spine heads in KO neurons compared with LifeAct-GFP in WT neurons (Fig. 6g). Given this, we asked whether increasing RhoA activity in *Taok2* KO neurons could rescue the reduction in F-actin stability. We tested a commercial chemical activator (CN01) and found elevated levels of RhoA-GTP in the neuroblastoma cell line SHSY5Y (arrow, Fig. 6f). We used this compound on DIV14 *Taok2* KO neurons and measured F-actin dynamics before and after addition. The RhoA activator significantly increased F-actin distribution toward accumulation in dendritic spine heads in *Taok2* KO cortical neuron cultures, concurrently decreasing the relative spine motility (Figs. 6g, h; relative motility normalized to Lifeact-GFP-transfected KO cells without treatment; Supplementary Video 3). The reduction in spine motility was accompanied by the elimination of spine collapse, concurrent with the formation of stable spines bearing a spine-head with actin accumulation in *Taok2* KO neurons (Fig. 6i). We then analyzed whether activating RhoA in *Taok2* KO cortical neurons could rescue the deficiency in dendritic spine density. Short-term incubation with the RhoA activator (30–60 min) increased the number of dendritic synapses (spines with SynGAP co-staining) in *Taok2* KO cells compared with dimethyl sulfoxide (DMSO) treated *Taok2* KO cells (Figs. 6j, k). Importantly, incubation of WT neurons with the RhoA activator did not show any significant increase, but did show a nonsignificant decrease in SynGAP-positive dendritic spines, highlighting an optimal range of RhoA activity is necessary for proper spine formation (Fig. 6k, WT + DMSO vs. WT + CN01(60’) *p* = 0.0611). Given that increasing RhoA activity rescued the Taok2-dependent synaptic defects, we asked whether Taok2 bound in a functional complex with RhoA. We immunoprecipitated Taok2 from both a crude homogenate and a crude membrane fraction of WT mouse cortices to determine whether Taok2 interacts with RhoA. Our results show that RhoA is in the same protein complex as Taok2 (Supplementary Fig 17a). Interestingly, immunoprecipitation of RhoA after overexpression of TAOK2α and β isoforms and the de novo mutants in HEK293 cells, reveal that TAOK2β preferentially binds RhoA compared with TAOK2α (Supplementary Fig. 17b, c). Furthermore, the binding of TAOK2β to RhoA is affected by the P1022* mutation, suggesting that the C-terminal domain, which differs between TAOK2α and β, is important for RhoA binding (Supplementary Fig. 17b, c). Our results reveal that Taok2 regulates the filopodia–spine transition through RhoA activity and the activation of RhoA is sufficient to overcome spine deficits in *Taok2* KO cultures.

## Discussion

Previous studies have implicated *TAOK2* in NDDs but there has been no comprehensive study on *TAOK2* to support this. Our data provide novel evidence that *TAOK2* is directly associated with ASD pathologies, such as deficits in social interaction, enlarged brain volume, and reduced dendritic growth and spine formation, and identifies a mechanistic pathway regulating synapse function. Our results show that Taok2 regulates RhoA activation, and loss of Taok2 leads to abnormal actin dynamics, stability and organization that may contribute to the synaptic defects observed in *Taok2* KO mice. Our data also provide new insight into the spatial role of Taok2 in the mouse brain. Loss of Taok2 has the strongest effect on excitatory neurons in the PFC and, by a smaller degree, in the somatosensory cortex, but shows no effect in the HC. This is consistent with a recent publication showing that knockdown of *Taok2* in hippocampal neurons reveals no electrophysiological phenotype [48].

One of our main findings is that alterations in TAOK2 activity contribute to NDDs. First, *Taok2* KO mice have several behavioral, anatomical, and synaptic deficits consistent with other ASD mouse models [49–53]. Second, we identified and characterized novel de novo mutations in *TAOK2* in human ASD cohorts revealing that the mutations impact different signaling pathways (Supplementary Fig. 18a). Our results show that the A135P mutation reduces TAOK2 activity indicating a LOF mutation, and causes a reduction in JNK1 activation, decreased RhoA activity and reduced dendrite growth and spine maturation. Whereas the P1022* mutation is a GOF mutation, which increases TAOK2-dependent activation of JNK1, thus enhancing dendritic growth and branching. Functional differences between the mutations are expected given that auto-phosphorylation of the kinase domain is important for TAOK2 activation, whereas the C-terminus negatively regulates kinase activity [78, 79]. Therefore, either a reduction or elevation in *TAOK2* gene dosage could be pathogenic, consistent with deletions or duplications of the 16p11.2 locus, which harbors *TAOK2* and confers a risk for ASD and schizophrenia, respectively [8, 14]. This is also consistent with analysis of cortical neurons from a 16p11.2 duplication mouse that displayed excessive dendrite outgrowth, similar to the *TAOK2* P1022* mutation [83]. Although the individual with the *TAOK2* P1022* mutation also has a *CHD8* mutation, they have a more complex phenotype when comparing scores for adaptive behavior and IQ compared with the other subjects with TAOK2 mutations, suggesting that both *TAOK2* P1022* and *CHD8* may contribute to this subject’s phenotype.

Additional support for the contribution of *TAOK2* to the development of neuropathologies comes from a recent report identifying two de novo mutations in *TAOK2* in individuals with a complex developmental (and neurological) phenotype [84]. This may be relevant for 16p11.2 deletion/duplication carriers who do not develop ASD/schizophrenia, but the Het deletion/duplication of *TAOK2* may be sufficient for a NDD in these individuals. Importantly, the *TAOK2* de novo mutations we characterized are not found in the ExAC database, indicating these mutations are more likely pathogenic. TAOK2 is also highly intolerant to LOF and missense mutations and it is known that genes within CNVs have a different mechanism of mutation compared with genes outside of CNVs [85]. Therefore, the identification of three de novo mutations and three other truncating mutations in the *TAOK2* gene is highly suggestive of its significance to NDDs including ASD.

Given the results of our study and due to *TAOK2* being localized in the 16p11.2 CNV, it is conceivable that it may contribute to disease pathophysiology. For example, we found an increase in midbrain volume in *Taok2* KO mice, similar to the 16p11.2/+ mice, however, the opposite effect is observed for total brain volume [86–88]. Interestingly, patients with the 16p11.2del CNV show measurable increases in head circumference, gray matter, and white matter in the cortex and thalamus [89–92], which coincides with the increase in total brain volume and in the thalamus and midbrain volumes in *Taok2* Het and KO mice, but not with the 16p11.2del mice. The somatosensory cortex region has not been analyzed in 16p11.2del mice, however, a volumetric decrease in the general frontal lobe has been observed [88]. Furthermore, studies have shown 16p11.2del CNV patients have increases in the corpus callosum volume, which is dissimilar to the changes seen in the *Taok2* KO mice, indicating TAOK2 may only affect specific cortical structures in individuals with the 16p11.2 CNV [93, 94]. Interestingly, although KCTD13 is proposed to be a driver gene of head size in the 16p11.2 CNV [10], a recent study using *Kcdt13* KO mice revealed no brain size changes [13], suggesting instead that TAOK2 along with other genes may regulate this phenotype. KCTD13 also regulates RhoA signaling in the brain, suggesting that elevated RhoA levels in the 16p11.2 deletion are pathogenic and caused by KCTD13 [11]. In comparison with TAOK2, cortical lysates from *Taok2* KO mouse brains and patient-derived LCLs exhibit decreased RhoA activity, indicating that TAOK2 and KCTD13 may regulate RhoA in different brain regions or at different developmental time points. Interestingly, as we found that TAOK2 binds in a functional complex with RhoA, and RhoA is a known substrate of KCTD13, this raises the possibility that TAOK2 and KCTD13 may regulate one another. Importantly, additional genes in the 16p11.2 CNV are also thought to contribute to the disease phenotype, including *MAPK3* and *SEZ6L2* [6, 7, 10, 11, 83]. Given the large 16p11.2 CNV region and the complexity of neurological phenotypes associated with it, it is likely that multiple genes play a role, similar to other CNVs [95].

In summary, we have characterized *Taok2* Het and KO mice using behavioral assays for ASD-associated phenotypes, MRI to identify gross brain morphological changes and functional studies that identified cellular deficits causing altered neural morphology, connectivity, and activity through a RhoA-dependent pathway (Supplementary Fig. 18b). In addition, we identified human-derived de novo LOF/GOF mutations and studied their impact using human and mouse in vitro and in vivo systems (Supplementary Fig. 18b). Our study defines *TAOK2* as a novel NDD risk gene and provides novel data that demonstrate how patient-derived mutations impact brain function and development. Given this, it is important to determine if other ASD or NDD cohorts possess pathogenic variants or mutations in *TAOK2* to better understand its contribution to disease.

## Electronic supplementary material

Supplemental Material

Video 1

Video 2

Video 3

Supplementary Table 1
