# Supplementary material for: Altered TAOK2 activity causes autism-related neurodevelopmental and cognitive abnormalities through RhoA signaling
Source: Mol Psychiatry. 2018 Feb 21;24(9):1329–50. doi: 10.1038/s41380-018-0025-5 (PMC6756231; doi:10.1038/s41380-018-0025-5)
Supplement: Supplementary file 1 — Supplemental Material [file 41380_2018_25_MOESM1_ESM.pdf]

Material & Methods

Animals

C57BL6/J Taok2 KO (*Taok2* -/-) mice were generated and described by Kapfhammer et al (1). Briefly, to generate a conditionally disrupted *Taok2* allele, lox P sites were introduced flanking exons 2 through 7 of the *Taok2* gene. Within intron 7, a neomycin resistance gene was inserted, flanked by lox P and frt sites. Upon crossing this line to mice expressing Flpe recombinase, the neomycin selectable marker was removed along with one of the lox P sites to generate the *Taok2tm1fl* allele. In the presence of Cre recombinase, the remaining lox P sites were recombined into a single lox P site, removing exons 2 through 7 and the translational start site to generate the *Taok2tm1Δ* allele. Animals were bred, genotyped and housed at the Central Animal Facility at McMaster University and University Medical Center Hamburg-Eppendorf, Hamburg. All procedures received the approval of the Animal Research Ethics Board (AREB) and the Institutional Animal Care and Use committee of the City of Hamburg, Germany (G48/13 and G43/16 acc. to the Animal Care Act, §8 from May, 18th 2006). Genotypes were identified during breeding by PCR of ear notches or tail biopsies. Two *Taok2* females were bred with one *Taok2* male per breeding cage. Both female and male *Taok2* Het and KO mice were used for experiments, only males were used for behavior analysis and dendritic and spine analysis after Golgi-Cox staining. 3-weeks old mice (P21-P24) were used after IUE and for western blot and RhoA GTPase experiments, 1- to 52-weeks old mice were used for live MRI imaging, and 8- to 10-weeks old mice were used for fixed brain MRI imaging. To obtain cortical cultures, *Taok2* Het females were timed bred with males and males were removed when a plug was observed, indicating copulation. At E16, mothers were sacrificed and brains from embryos were collected. Animals of appropriate genotype were included. No randomization was used and blinding was only used during animal behavioral analysis, EM analysis and spine motility analysis.

DNA constructs

The following constructs were used in these studies for transient expression in cell lines after liposomal transfection or for expression in neurons after Amaxa electroporation or *in utero* electroporation, respectively: Wild-type TAOK2α (RC214297, NM_016151) and β isoforms (SC117141, NM_004783.2) were purchased (Origene, Rockville, MD). Human-derived

mutations in TAOK2 were generated by site-directed mutagenesis (Agilent, Santa Clara, CA). cDNA sequences of WT or mutated TAOK2 were re-cloned into a recombinant adeno-associated virus (rAAV)-vector under control of a chicken β-actin promoter (pCAGIG) (2). Silencing RNA shRNA ctrl and TAOK2 shRNA were used as described before (3). Palmitoylated EGFP plasmid originates from Clontech, (Mountain View, CA, 6085-1). Flag-Jnk1a1 (pCDNA3) was a gift from Roger Davis (Addgene, Cambridge, MA; #13789); LifeAct-GFP from Dr. Frank Bradke (4, 5) and GST-Rhotekin-RBD (pGEX) from Dr. Elena Pasquale (6).

Antibodies

The following antibodies were used in these studies for immunocytochemical or western blotting analysis: mouse anti α-tubulin (Abcam, Cambridge, UK; ab7291, ICC 1/500, WB 1/5.000); goat anti-TAOK2 (Santa Cruz Biotechnology, Dallas, TX; sc-47447, ICC 1/200, WB 1/1.000); rabbit anti-pTAOK2 (ser181) (Santa Cruz Biotechnology, sc-135712, WB 1/1.000; R&D Systems, Minneapolis, MN; PPS037, WB 1/1.000); mouse anti-RhoA (Santa Cruz Biotechnology, sc-418, WB 1/100); mouse anti-pJNK1/2 (Cell Signaling Technology/NEB, Danvers, MA; 9255L, WB 1/250); mouse anti pan-JNK (BD Transduction Laboratories, San Jose, CA; 610627, WB 1/500); rabbit anti-SynGAP (Synaptic Systems, Göttingen, Germany; ABR-01255, ICC 1/200, WB 1/1.000); rabbit anti-Synaptophysin-1 (Synaptic Systems, 101002, WB 1/1.000); mouse anti PSD-95 (Millipore, Billerica; MA; MABN68, WB 1/1.000); rabbit anti-CUX-1 (Santa Cruz Biotechnology, sc-13014, IHC 1/200); rat anti-Ctip2 (Abcam, ab18465, IHC 1/50); mouse anti-β-actin (Sigma, St. Louis, MI; A5316, WB 1/5.000); rabbit anti-FAK (Santa Cruz Biotechnology, sc-271195, WB, 1/5.000); mouse anti-GAPDH (Santa Cruz Biotechnology, sc-32233, WB 1/5.000) and chicken anti-GFP (2B Scientific, Upper Heyford, UK; GFP-1020, WB 1/5.000). Nuclei were visualized with Hoechst (Invitrogen, Carlsbad, CA; 33258, ICC/IHC 1/10.000) and F-actin with rhodamine-conjugated Phalloidin (Cytoskeleton, Denver, CO; PHDR1, ICC 1/300). HRP- (Dianova, Hamburg, Germany; WB 1/1.000-1/5.000) and Alexa-conjugated secondary antibodies (Life Technology, Carlsbad, CA; ICC 1/1.000).

Magnetic Resonance Imaging of fixed brains

8- to 10-weeks old mice were perfused with 4% PFA (with 2mM Prohance) in PBS. Mice were genotyped after perfusions and only mice with clear genotypes were used. Brains were left intact

in the skull, and the zygomatic bone and muscles were removed. Brains were then left in 4% PFA (with 2mM Prohance) in PBS overnight and transferred to PBS (with 0.02% Sodium Azide and 2mM Prohance) and kept at 4°C. All mouse imaging was done at the Mouse Imaging Center (The Hospital for Sick Kids). A multi-channel 7.0 MRI scanner (Varian Inc., Palo Alto, CA) was used to image the brains within skulls. Sixteen custom-built solenoid coils were used to image the brains in parallel (7). Parameters for the anatomical MRI scans are as follows: T2-weighted, 3D fast spin-echo sequence, with a cylindrical acquisition of k-space, and with a TR of 350ms, and TEs of 12ms per echo for 6 echoes, field-of-view of 20x20x25 mm^3 and matrix size = 504x504x630 giving an image with 0.040mm isotropic voxels. Total imaging time is currently 14h. To visualize and compare any changes in the mouse brains the images are linearly (6 parameter followed by a 12 parameter) and non-linearly registered together. All scans can then be resampled with the appropriate transform and averaged to create a population atlas representing the average anatomy of the study sample. The result of the registration is to have all scans deformed into alignment with each other in an unbiased fashion, allowing for the analysis of the deformations needed to take each individual mouse's anatomy into the final atlas space to model how deformation fields relate to genotype (8, 9). The jacobian (a measure based on the deformation of each brain indicating expansion or contraction at that voxel) determinants of the deformation fields are then calculated by warping a pre-existing classified MRI atlas onto the population atlas. These measurements were then examined on a voxel-wise basis in order to localize the differences found within regions or across the brain, in a total of 182 different regions. Multiple comparisons in this study were controlled for using the false discovery rate (10).

Magnetic resonance imaging (MRI) of live mice

MRI was performed using a dedicated 7 Tesla small animal MRI (ClinScan, Bruker, Ettlingen, Germany) with a mouse head 4 element phased array receiver surface coil and a linear polarized rat body transmit coil. The animals were anesthetized with a gas mixture of oxygen and about 1% isoflurane. The oxygen was delivered with a flow rate of 0.5L/min and the isoflurane was applied via a vaporizer (Föhr Medical Instruments, Seeheim-Oberbeerbach, Germany). The animal's respiratory rate (about 100/min) was monitored using a small animal monitoring system (SA Instruments, Stony Brook, NY). Animal body temperature was not monitored, but a pad

with circulating water of 37°C covering the back of the animal helped maintaining the body temperature during MRI. MRI for volumetric analysis was done using a 3D constructive interference steady state (CISS) sequence with echo time = 3.87 ms, repetition time = 7.74 ms, flip angle = 50°, readout bandwidth = 200 Hz/pixel, 4 averages, field of view = 16x16x14.4 mm^3, matrix = 128x128x120, elliptical k-space sampling and 12:25 min scan time. MRI for microstructural analysis was done using diffusion tensor imaging (DTI) with a diffusion weighting b-value of 1000 s/mm^2 and 12 spatially non-concordant diffusion weighting directions, echo time = 43 ms, repetition time = 20000 ms, flip angle = 90°, readout bandwidth = 2790 Hz/pixel, 2 averages, field of view = 19x19 mm^2, matrix = 128x128 with 5/8 partial Fourier acquisition, 30 slices with 0.5 mm slice thickness, no gap between slices and 9:00 min scan time. The DTI parameters apparent diffusion coefficient (ADC) and fractional anisotropy (FA) were calculated using the FMRIB Software Library (FSL) v5.0 software tools *eddy_correct* for correcting eddy currents and movements (11) and *dtifit* for fitting the diffusion tensor model to each voxel (12). Image analysis was done using MRIcron (13). Volumes of interest (VOIs) were manually defined for each group (WT, HET, KO) at each time point (PN8, 4-, 16- and 52-weeks of age) on mean CISS or b=0 DTI data sets comprised from realigned data sets of all animals of each group and time point. (Fig. 1c-f and Supplementary Fig. 1k-o). The mean data sets were computed by realigning all individual data sets to one reference data set within the cohort. The FSL tool *flirt* was used for data realignment for improved optimization for the robust and accurate linear registration and motion correction of brain images (14). The reference data set was manually chosen based on the criteria of good image quality, coverage, position and orientation. The realigned data sets were then averaged using the FSL tool *fslmath* to generate the mean data set. Mean data sets were separately calculated for each group at each time point since brains differ too much between groups and time points and allow no sufficient realignment. The VOIs defined on the mean data sets were transferred to the individual image data using the inverse transformation matrix previously calculated by the *flirt* FSL tool. The volumes of the individual VOIs of the CISS data and the median ADC and FA values of these VOIs in the individual DTI data were extracted using an in house written *Matlab* (The Mathworks, Natick, MA, USA) script. Furthermore, the curvature of the cortex VOIs defined in the CISS data was also extracted using an in house written *Matlab* code (Fig. 1d). Briefly, a surface was fitted to the top points of the cortex VOI using the locally weighted scatter plot smoothing algorithm with the

quadratic regression model (*loess*), then the mean and the Gaussian curvature was calculated and averaged over the midline cortex surface area.

In vivo electrophysiology

Animals. All *in vivo* local field potential (LFP) were performed in P7-10 WT controls or P8-10 TAOK2 KO mice. TAOK2 KO mice had significantly smaller body weight at the time of recording than WT pups at similar age (WT: 4.78 ± 0.19 g, n=11, KO: 4.12 ± 0.12 g, n=12, p=0.019). Hence to avoid that any differences between the groups could result from the lower body-weight of the KO mice, an additional control group was matched for body weight (WT: 4.03 ± 0.82 g, n=9, KO: 4.12 ± 0.12 g, n=12, p=0.57) by including younger P7 mice. However, this resulted in a significantly different age distribution between the groups (WT: 7.78 ± 0.32 days, n=9, KO: 9.25 ± 0.22, n=12, p=0.002).

Surgery. Mouse pups were initially anesthetized with isoflurane (induction 5% in O_2) followed by i.p. administration of urethane (1 g/kg body weight). The head of the pup was fixed into the stereotaxic apparatus using two small metal bars fixed with dental cement on the nasal and occipital bones, respectively. The bone over the regions of interest (prelimbic subdivision (PL) of the PFC, intermediate HC) was carefully removed by drilling holes of less than 0.5 mm in diameter. Removal of the dura mater by drilling was avoided, since leakage of cerebrospinal fluid or blood damps the cortical activity and neuronal firing. The body of the animal was surrounded by cotton and kept at a constant temperature of 37 °C by placing it on a heating blanket. A local anesthetic (0.25% bupivacaine/1% lidocaine) was administrated. After 20-30 min recovery period, multi-site electrodes (NeuroNexus Technologies, Inc., Ann Arbor, MI, USA) were inserted perpendicularly to the skull surface into PL until a depth of 1.7-2.5 mm and at a 20° from the vertical plane into HC at a depth of 1.2-1.7 mm. In each experiment, the electrodes were labeled with DiI (1,1'-dioctadecyl-3,3,3',3'-tetramethyl indocarbocyanine, Invitrogen) to enable post-mortem in histological sections the reconstruction of electrode tracks in PFC and HC (Supplementary Fig. 3a,d). Two silver wires were inserted into cerebellum and served as ground and reference electrodes.

Recording protocols. Simultaneous recordings of LFP and multi-unit activity were performed from the prefrontal subdivision PL (0.5-0.7 mm anterior to bregma and 0.3-0.5 mm from the

midline) and the CA1 area of the intermediate HC (3.5-3.7 mm posterior to bregma, 3.5-3.8 from the midline) using similar protocols as described previously (15). One shank Michigan electrodes with 16 recording sites (0.5-3 MΩ) that were separated by 50 μm (for HC) or 100 μm (for PL) were used. The position of recording sites over the PL and CA1 area was confirmed by post-mortem histological evaluation. Both LFP and MUA were recorded for at least 60 min at a sampling rate of 32 kHz using a multi-channel extracellular amplifier (Digital Lynx 4S with no gain, Neuralynx, Bozeman, MT, USA) and the Cheetah acquisition software (Westlake Village, CA, USA). During recording the signal was band-pass filtered between 0.1 Hz and 5 kHz.

Data analysis. Channels for analysis were selected on the basis of post-mortem histological investigation, i.e. which recording sites of fluorescently marked electrodes were confined to PL and hippocampal CA1, and the presence of specific patterns of activity. In the PL, these patterns were nested theta bursts with superimposed beta-low gamma oscillations, whereas in the HC the reversal of LFP over Str. pyramidale was used for the selection of the channel with sharp-waves of minimum amplitude and consequently, lowest contribution to the spectral content of the signal. Data were imported and analyzed off-line using custom-written tools in Matlab software version R2015a (The Mathworks, Natick, MA, USA). For LFP analysis, the signals were low-pass filtered (<1500 Hz) using a third order Butterworth filter before reducing the sampling rate to 3200 Hz. The detection and classification of discontinuous patterns of activity in the neonatal PL and hippocampal CA1 area were performed using a modified version of the previously developed algorithm for unsupervised analysis of neonatal oscillations (16) and confirmed by visual inspection. Signals were filtered between 4-100 Hz using a zero phase filter. Fragmented detection of oscillations was avoided by considering events with inter-event intervals <100 ms for PL and <300 ms for HC as single events. Only oscillatory events lasting >1 s in PL and >1.5 s in HC and containing at least three cycles were considered for further analysis. The detected events in PL and HC were analyzed in their occurrence (defined as the number of bursts per min), duration, mean amplitude and maximum amplitude (defined as the voltage difference between 0 and the maximal positive peak) and power distribution. Power spectral density estimates of every detected oscillatory event were calculated on the raw signal using the MATLAB function 'FFT' and averaged over all events from one animal. Mean power in different frequency bands (4-12 Hz, 12-30 Hz, 30-100 Hz) were quantified. Time-frequency

plots were calculated by transforming the LFP events using the function 'WT' from the wavelet coherence Matlab toolbox. As spectral measure of correlation between two signals coherence was calculated from the cross-spectral density and normalized by the individual power spectral density of each. The computation was performed using a multi-taper approach implemented in the Chronux toolbox (chronux.org) by the function 'coherencysegc' on 4-100 Hz filtered data. For this a continuous signal for either PL or HC was generated by gluing as many multiples of 2s-long segments that could be fitted of all co-occurring oscillatory bursts in either PL or HC into single vectors. Frequency domains with significant coherence were determined by Monte Carlo simulation. For this, LFP segments of 2 s from one region were shuffled and the coherence was calculated between the shuffled LFP from one region and the original LFP from the other region. After 100 iterations, the 95th percentile of the resulting distribution was used as significance threshold. The mean coherence for all frequency components (4-12 Hz, 12-25 Hz and 25-40 Hz) of oscillatory events that were detected as peaks in the power spectra were calculated.

Statistics. Statistical analyses were performed with IBM SPSS Statistics version 21 (SPSS GmbH). Generally all values were tested for normal distribution by the KOlmogorov-Smirnov test. For normally distributed values unpaired t-test was used. For not normally distributed values the Shapiro-Wilk test was used.

In situ electrophysiology

As described previously (17), coronal brain slices (400 µm) were prepared in cold sucrose-based slicing solution containing (in mM): 160 sucrose, 2.5 KCl, 10 $MgSO_4$, 1.25 NaH_2PO_2, 25 glucose, 30 $NaHCO_3$, 20 hepes, 5 Na-ascorbate, 3 Na-pyruvate, 2 thiourea, 0.5 $CaCl_2$. Slices recovered for 45 minutes at 30°C, followed by 45 min at room temperature. Visually guided whole-cell recordings (BX51WI, Olympus, Tokyo, Japan) were performed using an Axoclamp 700B amplifier (Molecular Devices, Sunnyvale, CA) from patch electrodes (P-97 puller, Sutter Instruments, Novato, CA) containing a cesium based intracellular solution (in mM): 100 CsCl, 100 gluconic acid, 10 hepes, 0.5 EGTA, 10 Na-phosphocreatine, 2 MgATP, 0.5 NaGTP, pH 7.3 with CsOH). Composition of aCSF (in mM): 120 NaCl, 2.5 KCl, 1 MgSO4, 26 NaHCO3, 10 glucose, 2 $CaCl_2$. 1 µM TTX and 100 µM picrotoxin was added to the bathing medium to block Na-dependent action potentials and GABA currents, respectively for mEPSCs. 1 µM TTX and

1mM Kynurenic Acid was added to the bathing medium to block Na-dependent action potentials and mEPSCs, respectively for mIPSCs. Recordings were performed at -70 mV for mEPSCs and +10mV for mIPSCs using Clampex 10.6 (Molecular Devices), corrected for a calculated -10 mV junction potential and analysed using the Template Search function from Clampfit 10.6 (Molecular Devices, Sunnyvale, CA).

Behavior analysis

Animal Husbandry. Taok2 6- to 8-weeks old male WT, Het and KO littermates from heterozygous breeding pairs were transferred from the breeding facility into a *vivarium* with an inverted 12h/12h-light/dark cycle (light off at 8:00 am) and maintained under standard housing conditions (21 ± 1°C, 40-50% humidity, food and water *ad libitum*). Behavioral experiments were performed with 10- to 18-weeks old mice during the dark cycle in a room next to the *vivarium* illuminated with dim red light. Tests started and ended at least two hours after light offset and three hours before light onset, respectively. The experimental material was cleaned with soap, water, and ethanol (70%) before and after each contact with an animal. To avoid a "litter effect", no more than two animals per genotype were used from the same litter. Tracks representing the position of the mice were created and analyzed with the software EthoVision (Noldus, Wageningen, The Netherlands) as described (18). Manual scoring of behavior was performed by a trained experimenter blind to the genotype of the mice using the *The Observer* software (Noldus). The experimenter trained himself until he repeatedly scored at least 90% of consistency between two analyses performed at different times on the same mouse, as calculated with the Reliability Test provided by the software The Observer (having 1s as maximal time discrepancy between two evaluations).

Open field. The open field test was performed in a box (50 x 50 cm and 40 cm high) illuminated with white light (25 lux). Mice were started from one corner of the box and videorecorded for 30 min. Distance moved, mean minimal distance to walls, time spent in the center (an imaginary 25 x 25 cm square in the middle of the arena) were analyzed with the software EthoVision (Noldus), whereas the parameters rearing on wall (the mouse stands on the hind limbs and touches the wall with at least one fore paw) and self-grooming were analyzed with The Observer (Noldus).

Elevated plus maze. The maze had the shape of a plus with four 30 cm long and 5 cm wide arms, connected by a squared center (5 x 5 cm). Two opposing arms were bordered by 15 cm high

walls (closed arms), whereas the other two arms (open arms) were bordered by a 2 mm rim. The maze was elevated 75 cm from the floor and an infrared camera allowed video recording under total darkness. The mouse was placed into the center facing one open arm and left on the maze for 5 min. The following parameters were analyzed with The Observer: entries into the open and closed arms (calculated when all the four paws were on an arm), total transitions (the sum of entries into the open and closed arms), rearing and self-grooming.

Social preference. Motivation to investigate a social stimulus was tested by giving the experimental mouse the choice to investigate a beaker containing an unfamiliar sex-matched mouse or an empty beaker. The apparatus consisted of a squared box (50 cm x 50 cm and 40 cm high) illuminated with white light (10 lux). Two beakers made of transparent plastic (diameter 10 cm, 15 cm high) with several holes (diameter= 1 cm) drilled at the bottom were placed at two opposite corners of the box. An unfamiliar adult male mouse was confined in one of the two beakers. The test started by placing the experimental mouse next to the beaker containing a male mouse and lasted 5 min. Distance moved and time at the beakers were analyzed with EthoVision, whereas rearing, self-grooming and time spent sniffing the two beakers were analyzed with The Observer.

Spontaneous alternation. The spontaneous alternation paradigm was performed in a Y maze to test for working memory performance (19). The maze consisted of three equally sized arms (34 cm x 5 cm x 30 cm) made of transparent Plexiglas connected such as to make a Y and illuminated with 20 lux. Mice were placed in the center of the maze and allowed to freely explore the maze until they performed 27 transitions or after a maximal given time of 20 min. An entry into any arm with the four paws was considered as transition. An entry into a new arm after having visited the two other arms was considered as alternation. Data were analyzed as percentage of alternations over all transitions. Average time to make a transition was calculated by diving the duration of the test by the number of total transitions.

Spatial object recognition. This test was designed to test short-term spatial memory. The paradigm is based on the same assumption as the spontaneous alternation test, namely that rodents have an intrinsic tendency to investigate novel stimuli. In other words, the test is a sort of delayed-no-match-to-place paradigm (19). The spatial object recognition test was performed in the same box used for the open field test divided into two equal compartments by a white PVC wall with a hole in the middle to allow a mouse to move between the two compartments. The

arena was illuminated w6th white light (15 lux). The paradigm consisted of two trials, the exposure (10 min) and the test (5 min) trials with an inter-trial interval of 10 min during which mice were returned to their housing cage. During the exposure trial two identical objects unfamiliar to the mice were placed in one corner of each compartment. During the test trial one of the two objects was displaced to another corner of the compartment. Distance moved, mean velocity and time spent at the objects were analyzed with Ethovision. Sniffing the objects, self-grooming, rearing on wall and rearing on objects were analyzed with The Observer.

Contextual fear conditioning. In the contextual fear conditioning test mice had to learn the association between the unconditioned (electric foot-shock) and conditioned (context) stimuli. Mice were conditioned in the context, a chamber (23.5 cm x 23.5 cm and 19.5 cm high) with Plexiglas walls and ceiling and a stainless grid floor from which an electric shock could be elicited. The chamber was illuminated by white light (20 lux). Mice were placed in the center of the cage and received four electric foot shocks (0.35 mA, 1 s) at 120, 160, and 200 s. At 220 s the recoding ended, and the mouse was immediately returned into its home cage. Mice were tested for long-term memory retention one day after conditioning. The conditioned response was analyzed by quantifying the percentage of time spent freezing (defined as absence of body movements for at least 1 s). Freezing behavior was automatically analyzed using a modified version of the system Mouse-E-Motion (Infra-e-motion, Hamburg, Germany).

Water maze. The water maze consisted of a circular tank (145 cm in diameter) circled by dark curtains. The water was made opaque by the addition of non-toxic white paint such that the white platform (14 cm diameter, 9 cm high, 1 cm below water surface) was not visible. Four landmarks (35 X 35 cm) differing in shape and grey gradient were hung on the wall of the maze. Light was provided by four white spotlights placed on the floor around the swimming pool that provided homogeneous illumination of 60 lux on the water surface. Before the experiment started, mice were familiarized for one day to swim and climb onto a platform (diameter of 10 cm) placed in a small rectangular maze (42.5 x 26.5 cm and 15.5 cm high). During familiarization the position of the platform was unpredictable since its location was randomized and training was performed under darkness. After familiarization mice underwent three learning days during which they had to learn the location of a hidden platform. Starting position and position from which mice were taken out of the maze were randomized. At day 1 and 2 mice underwent four learning trials (maximum duration 90 seconds, inter-trial interval of 10 minutes). After staying on the platform

for 15 s, mice were returned to their home cage and warmed up under red light. Day 3 consisted of a 60 seconds long transfer trial (long-term memory transfer trial) during which the platform was removed.

Hippocampal and Cortical Cultures

Primary cortical (17) and hippocampal cultures (20) were prepared as described previously. When mouse cortices from heterozygous *TAOK2* mice were prepared, the cortices were kept in Hibernate E buffer (Life Technologies) until genotyping of the embryos was performed. Cortices from either WT or KO embryos were pooled. Tissue was digested in 0.25% Trypsin or 31.25µg/ml papain and 10µg/ml DNaseI for 10-20min at 37°C. Trypsination or papain digestion was stopped by adding DMEM + 10%FCS or Neurobasal + 10% FBS and washed three times. Dissociated neurons were either plated directly on poly-D-lysine and laminin pre-coated glass coverslips in Neurobasal/B27 medium (Life Technologies) or underwent Amaxa electroporation (Lonza/Amaxa, Cologne, Germany).

Amaxa Electroporation

Amaxa electroporation (Lonza) was carried out according to the manufacturer's protocol and described elsewhere (21). In detail, $5x10^6$ cells were resuspended in 100µl Nucleofector solution with a total of 3µg DNA or the indicated amount of shRNA added. Rat hippocampal neurons and mouse cortical neurons were transfected using the appropriate Necleofector kit using program G-013 or O-005, respectively. Transfected neurons were plated on glass coverslips in 24-well chambers (50,000 cells/well), pre-coated with 0.1mg/mL Poly-D-Lysine (BD Sciences)/3.3ug/mL Laminin (Sigma) for immunocytochemical analysis or on pre-coated 4-well LabTek chamber slides (300,000 cells/chamber, Sarstedt, Nürnbrecht, Germany) for time-lapse life imaging.

Immunofluorescence

Dissociated cultures: Neurons were maintained in culture for 14 or 15 days *in vitro* (DIV) to analyze spine formation, for 19DIV to perform spine rescue and spine motility assays or for 21DIV to analyze mature spine morphology and then fixed for immunofluorescence analysis. Neurons were fixed with 4% PFA/4% sucrose at RT for 10 min, followed by 3 washes with PBS.

After blocking in donkey serum (Sigma, St. Louis, MI), neurons were incubated with the primary antibodies. *Cortical sections:* Brains were removed and immersion-fixed overnight in 4% PFA/4% sucrose and thereafter transferred to sterile 30% sucrose/PBS (4 °C, overnight). Brains were embedded in OCT compound (Sakura Finetek, Tokyo, Japan), frozen at -80°C and sectioned in a cryostat (Leica, Wetzlar, Germany). 20- to 30-μm cryosections were incubated overnight at 4 °C with the primary antibodies.

Quantitative immunofluorescence of cultured cortical neurons

Neurons were cultured for 14DIV after plating and fixed as described above. Total TAOK2, filamentous actin, tubulin and the marker protein GFP_{palm} or Venus (pCAGIG) were visualized by indirect immunofluorescence. The mean intensity gray value of a line drawn along the soma and the neurites was measured using ImageJ. To measure TAOK2 localization, images were put under the same threshold value for each fluorescence channel; green for GFP and red for TAOK2. After threshold was set, total area encompassed by TAOK2 and GFP inside the dendrites or cell body was measured using ImageJ. Total area covered by TAOK2 was then divided by total GFP area of the dendrites or cell body alone.

Electronmicroscopy

Selected coronal vibratome sections (PFC, layer II/III, Bregma 1.94 to 1.34 mm, Interaural 5.74 to 5.14), primary somatosensory regions S1HL, S1Fl, S1BF and S1Tr (SSC, layer V, Bregma -1.46 to -1.94 mm, Interaural 2.34 to 1.86 mm), intermediate hippocampus (iHC, CA1, Bregma -2.54 to -2.80 mm, Interaural 1.26 to 1.00 mm) (22) were collected and prepared for electronmicroscopy as described before (23). Briefly, sections were rinsed three times in 0.1 M sodium cacodylate buffer (pH 7.2–7.4) and after osmication using 1% osmium tetroxide in cacodylate buffer, sections were dehydrated using ascending ethyl alcohol concentration steps, followed by two rinses in propylene oxide. Infiltration of the embedding medium was performed by immersing the tissue in a 1:1 mixture of propylene oxide and Epon and finally in neat Epon and hardened at 60°C. Semithin sections (0.5µm) were prepared for light microscopy mounted on glass slides and stained for 1 minute with 1% Toluidine blue. Ultrathin sections (60nm) were examined in an EM902 (Zeiss, Munich, Germany). Pictures were taken with a MegaViewIII digital camera (A. Tröndle, Moorenweis, Germany). Discrimination of dendritic versus spine

innervation was done acc. to (23-26). Briefly, we distinguished spine versus shaft synapses through determining the postsynaptic spine in 'spine synapses' as a dendritic spine that contains a clearly recognizable post-synaptic density (PSD), that does not contain mitochondria in close proximity to the PSD and that is filled with electron-dense material. Whereas in a 'shaft synapse', the postsynaptic site, defined by the presence of a PSD, is not as morphologically distinct as the spine head and is filled with rather electron-light material (clear appearance). Importantly, dendritic shaft innervation is often, but not necessarily, characterized by the presence of mitochondria in close proximity to the PSD.

Regional brain lysates

Indicated brain regions from postnatal 21- to 24-days old C57BL/6 mice were dissected and homogenized in 20x vol/weight of either sterile-filtered RIPA buffer (50mM Tris-HCl pH 7.4, 150mM NaCl, 1mM EGTA, 1% NP-40, 0.25% sodium deoxycholate, 10mM Na_3VO_4, 10mM NaF, containing proteinase inhibitors (Roche, Basel, Switzerland) or RhoA GTPase buffer (50mM Tris-HCl pH 7.2, 500mM NaCl, 10mM $MgCl_2$ x 6 H_2O, 1% Triton-100, 0.1% SDS, 10mM Na_3VO_4, 10mM NaF, containing proteinase inhibitors (Roche, Basel, Switzerland). To clear lysates, they were centrifuged at 20.500 x g for 10 min at 4°C. Supernatants were either applied to RhoA GTPase assay immediately or were analyzed by BCA method for concentration determination and afterwards applied to immunoprecipitation or SDS gel separation and western blotting.

Immunoprecipitation

400µg of total lysates for Taok2 immunoprecipitation or 1500µg of total lysates for co-immunoprecipitation of RhoA and Taok2, respectively, were incubated overnight at 4°C with 2µg of pre-conjugated polyclonal TAOK2 antibody (Santa Cruz). Antibodies were conjugated before to 15µl of magnetic Dynabeads™ Protein G (Invitrogen) for 2hrs at RT. The bound immune complexes were collected by a magnet, followed by 5 washes in sterile-filtered RIPA buffer. After the final wash, supernatants were completely removed and beads were eluted in 2x SDS-PAGE sample buffer, boiled for 5 min at 95 °C and separated on 4-20% SDS-polyacrylamide gradient gels (Invitrogen).

RhoA Pull Down

Recombinant GST-Rhotekin-RBD was expressed in freshly transformed DH5α-E. coli, purified through Glutathione-Sepharose 4B beads (Qiagen) as described elsewhere and stored in appropriate aliquots at -80°C (27). SHSY5Y cell lysates, freshly homogenized cortical or hippocampal brain lysates or LCL lysates were obtained in RhoA pull-down buffer and cleared by centrifugation for 10min at 4°C. The RhoA activation assay was performed using a commercial RhoA Pull-down kit (Cytoskeleton, Biochem Kit, #BK036, Denver, CO) or as described (21). Briefly, the supernatant was applied completely to ~50µg of freshly thawed recombinant GST-Rhotekin-RBD beads per pull-down and incubated for 45min at 4°C. A small portion of ~50µl of supernatant was saved before for detection of total RhoA in the lysate. After incubation, GST-Rhotekin-RBD beads were washed thoroughly for three times. After the final wash, supernatants were completely removed and beads were eluted in 2x SDS-PAGE sample buffer, boiled for 5 min at 95°C and separated on 4-20% SDS-polyacrylamide gradient gels (Invitrogen).

Transfection and Western blot analysis of transfected cell lines

HEK-293T and SHSY5Y cells (both Invitrogen, Carlsbad, CA) were grown under standard cell culture conditions. HEK-293T cells were transfected with plasmids using Lipofectamine™ LTX with Plus™ reagent according to the manufacturer's protocol (Invitrogen). For transient transfection, HEK293T cells were co-transfected with equal amounts of TAOK2 variants and JNK1a1 with Venus (GFP) or with TAOK2 variants only and Venus. The total amount of transfected DNA was 4µg per 1-well in a 6-well plate. The cells were allowed to express the constructs for 48 hours before lysis and analysis. SHSY5Y, a fast growing neuronal cell line, was used for testing the commercial RhoA activator (CN01, Cytoskeleton).

Proteins from cell lysates in RhoA GTPase buffer, containing proteinase inhibitors (Roche) or RIPA buffer, containing proteinase inhibitors (Roche) were either applied to RhoA GTPase assay immediately or were analyzed by BCA method (Thermo, Waltham, MA) for concentration determination and afterwards applied to immunoprecipitation or SDS gel separation and western blotting. Proteins were separated on 4-20% or 10% SDS-polyacrylamide gradient gels (Invitrogen) at 30mA/gel and transferred to Immobilon-P PVDF membranes (Millipore) at 35mV overnight at RT. Membranes were blocked in TBS-T (50mM Tris-HCl pH 7.4, 150mM

NaCl, 0.1% Tween-20) with 3% BSA, Fraction V (Roth, Karlsruhe, Germany) or with 3% skim milk powder for 1h at room temperature and then incubated with primary antibody for 2 h to overnight at 4 °C. Membranes were washed for 30 min in TBS-T and incubated for 2hrs at room temperature with horseradish peroxidase-conjugated secondary antibodies (Dianova or GE healthcare) and then washed for 30 min in TBS-T. Immunoreactivity signals were detected by enhanced chemiluminescence (INTAS, ChemoStar, Ahmedabad, India or Amersham ECL, GE Healthcare, Chicago, IL).

Lymphoblastoid cell lines (LCLs) were created using peripheral blood mononuclear cells isolated from the blood of probands and their families that were immortalized using the Epstein-Barr virus (28). Lymphoblastoid cell lines were cultured under standard suspension condition in RPMI 1640 media (with %15 FBS and 1mM sodium pyruvate). LCLs were cultured at 200.000 cells/ml in 5ml and media was doubled every 3-5 days until reaching 20ml. Cells were counted and 5 million cells were pelleted and lysed. Proteins from cell lysates were either applied to RhoA GTPase assay immediately or were analyzed by BSA method (Thermo) for concentration determination and afterwards applied to immunoprecipitation or gel separation and western blotting. Proteins were separated on 12% SDS-polyacrylamide Tris-glycine gels at 100V and transferred to Immobilon-P PVDF membranes (Millipore) for Western Blot analysis.

Quantitative droplet digitial PCR

cDNA was synthesized from RNA extracted from cultured lymphoblastoid cell lines using the Superscript III 1st strand cDNA Synthesis Kit (Thermo) following manufacturer's protocol. Three RNA extractions were performed per sample and cDNA synthesis carried out from each independently. Gene expression estimation of TAOK2 was performed using the QX200 Droplet Digital PCR system (Bio-Rad Laboratories, Hercules, CA) using TBP probe Hs00427621_m1 as an endogenous control in a duplex reaction mode. Two Taqman probes were used to measure TAOK2; Hs01586199, which targeted all isoforms and Hs01026246 which targeted isoform NM_004783.3 only. The 20ml gene expression reaction mix consisted of 10 ml of 2x ddPCR SuperMix for Probes (Cat No. 1863023, Bio-Rad Laboratories), 1 ml of the target assay (labelled with FAM), 1ul of the endogenous control assay (labelled with VIC), 6 ml nuclease free water and 2 ml of cDNA. The gene expressions assays were previously validated by temperature gradient to ensure optimal separation of target and control droplets. Cycling conditions for the

reaction were 95°C for 10 minutes, followed by 45 cycles of 94°C for 30 seconds and 58°C for 1 minute, 98°C for 10 minutes and finally a 10°C hold on a Life Technologies Veriti thermal cycler. Data were analysed using QuantaSoft v1.4 (Bio-Rad Laboratories). No template, no RT and Human Universal RNA controls were tested in parallel with the study samples. The ratio of total TAOK2 or TAOK2β to TBP mRNA per sample was calculated and compared to the average ratio of the parents and siblings in each family.

Golgi-Cox Staining

Brains were harvested whole from 3-weeks old male offspring on a C57BL/6 background and stained using the FD Rapid GolgiStain kit (FD NeuroTechnologies, Columbia, MD) according to the manufacturer's protocol. Briefly, brains were rinsed with double distilled water and then immersed in a 1:1 mixture of FD Solution A:B. Solution A:B was replaced within the first 24 hours and then kept for 2 weeks at room temperature in the dark. Brains were then transferred to FD Solution C and kept in the dark at 4°C for 48 hours. Solution C was replaced after the first 24 hours. Afterwards, in preparation for vibratome sectioning, individual brains were placed in sterile 30% sucrose at 4°C for 24 hours. Coronal sections of 120 µm thickness were cut and collected consecutively in a 24-well plate in PBS. This thickness enabled optimal staining and preservation of spines on secondary and tertiary dendritic segments while the complete basal dendritic arbor was acquired as needed for Sholl analysis. Sections were mounted on Superfrost Ultra Plus microscope slides (Thermo Scientific) and allowed to dry for 5-10 min. Sections were developed exactly as described in the FD Rapid GolgiStain instructions. The development solution should be prepared just before use combining FD Solution D:E:H_2O_{dd} in a 1:1:2 mixture. Bottle and staining jar must be always covered to prevent vaporization. Glass slides were immersed for 3-5 min, rinsed in $H2O_{dd}$ for two times, 4 min each and allowed to dry briefly. Each slide was embedded using 120-150µl Mowiol (Sigma).

In utero Electroporation

Time-pregnant mice were given a pre-operative dose of buprenorphine (0.05-0.01 mg/kg body weight) by subcutaneous injections at least 30 min before surgery. Animals were then anesthetized using 2.5% isoflurane/O_2 inhalation. Oxygen was delivered with a flow rate of 0.65L/min and the isoflurane was applied via a vaporizer (Föhr Medical Instruments, Seeheim-

Oberbeerbach, Germany). The uterine horns were exposed and TAOK2 expression plasmids mixed with Fast Green (Sigma) were microinjected into the lateral ventricles of the embryos. The expression plasmid concentration was three times that of the fluorescence marker Venus. Five current pulses (50ms pulse, 950ms interval; 35mV) were delivered across the heads of the embryos. Post-surgery, 2-3 drops of meloxicam (0.1-0.5 mg/kg body weight) were given orally through soft food for 96 hrs. The offspring was delivered and perfused at three weeks of age (PN21-24) for dendritic and spine analysis. Perfusion was carried out as described elsewhere (29) and brains were post-fixed with 4%PFA/4%sucrose overnight at 4°C. Coronal sections of 120 µm thickness were cut using a vibratome and collected consecutively in a 24-well plate in PBS.

Confocal Imaging

15-20 individual pyramidal neurons derived from two to three independent coronal sections per mouse containing the cingulate cortex, area 1 and 2 (cg1 and cg2) and the prelimbic cortex (PrL) of the prefrontal cortex (PFC, layer II/III, Bregma 1.94 to 1.34 mm, Interaural 5.74 to 5.14), the primary somatosensory regions S1HL, S1Fl, S1BF and S1Tr (SSC, layer V, Bregma -1.46 to -1.94 mm, Interaural 2.34 to 1.86 mm) or the intermediate hippocampus (iHC, CA1, Bregma -2.54 to -2.80 mm, Interaural 1.26 to 1.00 mm) (22) were imaged. Pyramidal neurons were identified by localization and their distinct morphology within the respective regions. Presence of a completely unclipped basal dendritic arbor was a prerequisite for selection and imaging of the cell. Z-stacks of Golgi-stained dendritic trees (60-90 µm total on Z-axis; optical section thickness per Z-section: 1µm) were taken at 60x magnification on a NiKOn Ti-E research microscope (Nikon, Tokyo, Japan) fitted with a CoolSnap HQ2 camera (Photometrics, Tucson, AZ). Z-stacks of GFP-labeled dendritic trees after *in utero* electroporation (IUE) (60-90 µm total on Z-axis; optical section thickness per Z-section: 1µm) were acquired at 40x on a Zeiss LSM 700 confocal laser-scanning microscope (Zeiss, Oberkochen, Germany).

Sholl Analysis

To perform Sholl-Analysis, images of basal dendritic arbors of Golgi-stained pyramidal cells of the cg cortex (PFC), SSC and intermediate CA1 were opened with Fiji (ImageJ) and first submitted to Phansalkars auto local thresholding (30) with the radius adjusted according to the

background of the images (10-15pxl). The thresholded images were then traced semi-automatically via the Simple Neurite Tracer plugin (31) with Hessian-based analysis enabled, resulting in 2D projections of the basal dendritic trees. The images of the basal dendritic projections were then analyzed via the Sholl Analysis plugin with radii set to 5µm intervals (v3.4.5) (32).

For images taken of cells labeled with cytosolic GFP, the images were not thresholded but directly traced via Fiji's (ImageJ) segmented line tool and saved as ROI's. These ROI's were then flattened to create an image of the traced basal dendritic tree and the resulting images were then analyzed via the Sholl Analysis plugin with radii set to 5µm intervals (v3.4.5) (32).

Spine analysis Golgi

Spine analysis was performed on images of selected cells, previously taken for Sholl Analysis, according to standardized parameters described (33). In addition, the whole basal dendritic tree was taken into account. Centroids of width measurements of spines were then utilized to create spine maps after matching the centroids of the somata at the origin and aligning the angular position of apical dendrites (34). To emphasize the spine distribution, alpha blending was set to 0.5. Additional read-out from the spine analysis was created by calculating the distance of each spine to the center of the respective cell aiming to visualize the distribution of spines over the distance to the soma. Furthermore, the total number of spines per cell was matched with the respective total basal dendritic length to obtain an estimate of general spine-density. To quantify dendrite spines morphology, we used a semi-automatized protocol (33) using Fiji's (ImageJ) selection tools combined with the ROI manager. Measurements of individual spines were taken according to the cited paper, in an unbiased fashion, and a semi-automated based algorithm categorized the spines. Briefly, spine morphologies were categorized based on spine-head width and length of spines and as a result of width to length ratio. According to these parameters, spines were classified into mushroom, thin, long-thin, filopodia and stubby spines. Branched spines were marked separately during the analysis. Cumulative distributions were employed to visualize parameters of whole populations. Additionally, the distance of each spine to the center of the respective cell was calculated to visualize the distribution of spines over the distance to the soma.

Spine analysis IUE

Images of the basal dendritic trees were re-taken at 40x (to cover the whole basal dendritic field in one image) for spine-analysis with the pinhole set to 1 airy unit (0.9µm sections) and imaging at 0.5 µm steps. To improve the local contrast, the size of images was adjusted through bicubic interpolation (doubling the number of pixel in each direction (2048px → 4096px). Spine analysis was then performed as described for Golgi-stained cells. Since different objectives and microscopes were used (60x vs. 40x, light-microscope vs. laser-scanning microscope) and the original thresholds for categorizing spines were applied to Golgi-stained cells (33), the distribution of width measurements from control cells derived from IUE versus Golgi-stained cells in SSC and iHC were compared. Through re-scaling, the width distribution could be matched to the distribution of width measurements from spines of Golgi-stained SSC and CA1 leading to a new threshold for mushroom spines of 0.46154 µm. Thresholding of length measurements were not adjusted to keep the benefits of a higher resolution through cytosolic GFP-labeling and confocal microscopy.

Heat-maps and assessment of dendritic coverage

Heat-maps were generated to create a visual representation using Fiji (ImageJ) on the basis of dendritic filaments of individual cells. The dendritic width of these filaments was reduced to one pixel, thus taking each dendritic section equally into account. Neurons were then matched in their orientation and aligned regarding their angular position of apical dendrite and the centroid of the soma. Subsequently, filaments were projected onto each other followed by the application of a mean filter (Radius = 5px). To put an emphasis on areas with a higher occurrence of dendritic material, a LUT (warm metal) was applied.

Spine Motility Assay Analysis

Live cell imaging experiments of dendritic filopodia/spines of DIV14 *Taok2* WT, KO and KO with CN01 (1 U/ml) neurons labeled with Lifeact-GFP were carried out in a defined volume of culture medium (Neurobasal/B27 medium (Life Technologies) at 37°C/5% CO_2 and imaged at 60x magnification with a Nikon Ti-E research microscope (Nikon, Tokyo, Japan) fitted with a CoolSnap HQ2 camera (Photometrics, Tucson, AZ). During the acquisition, dendritic segments were recorded with a 2-second frame rate for 5 minutes (capturing 151 frames in total). Cells

treated with the RhoA activator CN01 were imaged once, then treated with the activator and after activation immediately imaged for the second time to allow an optimal pairwise comparison.
For analysis, unmodified raw images were opened with Fiji's (ImageJ) for the motility analysis. Images were modified regarding pixel density and sharpness and corrected for bleaching with included plugins. To improve the local contrast, the size of images was adjusted: Scale 2 times in x and y coordinate to 2478 x 2080 pxl through bicubic interpolation; mask unsharp with a radius of 10 pixels and a mask weight of 0.6; bleaching correction with histogram matching. Due to lower fluorescence and higher signal background ratios in LifeAct-transfected cells, we reinforced the modification in the mask unsharp with a radius of 40 pixels and added an additional step of contrast enhancement (enhance contrast with between 0.1% and 1% saturated pixels applied on all 151 frames) in the rescue assay (Fig. 6g, h). To correct for rotation or general movement of the cell, we applied the "Template matching" plugin from Qingzong Tseng (https://sites.google.com/site/qingzongtseng/template-matching-ij-plugin) (35). We normalized correlation coefficient; search area: 0; subpixel registration and bicubic interpolation method for subpixel translation. Where applicable, we modified all the cells with similar parameters. We used the "Dendritic Filopodia Motility Analyzer" to perform the motility analysis (36). We selected spines on 3-5 comparably sized dendritic stretches around the soma in a medial distance from 10-70µm to the center of the soma and analyzed 10-15 spines per cell. As an upper and lower threshold, we adjusted the default values to have the spines covered in blue with as little black surrounding as background as possible. In order to reduce that influence of the background, we chose an area for the analysis starting at the basement of the spine surrounding the spine covering area as tightly as possible. We used the starting coordinate as a reference for extension/retraction analyses.

Statistical Analysis for Sholl Analysis

Figure 3b (top): WT vs Het, *p for 95 µm; WT vs KO, *p for 20 µm, **p for 25 µm. Figure 3b (bottom): WT vs Het, *p for 65, 70 µm, **p for 60 µm, ***p for 10, 15, 55 µm, ****p for 20-50 µm; WT vs KO, *p for 75, 80 µm, **p for 65, 70 µm, ***p for 60 µm, ****p for 10-55 µm.
Figure 5b (left): WT+Venus vs Het+Venus, *p for 40, 55, 60, 95 µm, **p for 65, 85, 90 µm, ***p for 70-80 µm; WT+Venus vs Het+TAOK2α/β, *p for 35 µm, **p for 10, 15, 30 µm, ***p for 20, 25 µm.

Figure 5b (right): Het+TAOK2α/β vs Het+Venus, *p for 5, 30, 70 μm, **p for 15, 20, 80 μm, ***p for 75 μm, ****p for 10 μm; Het+TAOK2α/β vs Het+ TAOK2α/β A135P, *p for 40, 45, 65 μm, **p for 50, 60, 70, 75 μm, ***p for 55 μm. Het+TAOK2α/β vs TAOK2β P1022*, *p for 70, 145, 150 μm, **p for 60, 80, 140 μm, ***p for 65 μm, ****p for 85-135 μm.

Supplementary Figure 8b (top): WT vs Het, *p for 25, 60 μm; WT vs KO, *p for 80, 120 μm, **p for 20 μm.

Supplementary Figure 8b (bottom): WT vs KO, *p for 35-50 μm.

Supplementary Figure 9b (top): WT vs Het, *p for 35 μm.

Statistical Analysis for Spine Analysis

Figure 3e: WT vs Het, *p for 45-50 μm, **p for 40 μm, ***p for 35 μm. WT vs KO, *p for 90 μm, ***p for 80-85 μm, ****p for 35-75 μm.

Figure 5d: TAOK2α/β vs Venus, *p for 25, 55 μm, **p for 30 μm; TAOK2α/β vs TAOK2β P1022*, *p for 45-50, 125μm, **p for 25-30, 40 μm, ***p for 35 μm; TAOK2α/β A135P vs Venus, *p for 45,50, 60 μm, **p for 40μm, ***p for 25, 35, 55 μm, ****p for 30 μm; TAOK2β P1022* vs Venus, *p for 20, 60, 70 μm, ****p for 25-55μm.

Supplementary Figure 9e (top): WT vs KO, *p for 65 μm.

1. Kapfhamer D, Taylor S, Zou ME, Lim JP, Kharazia V, Heberlein U. Taok2 controls behavioral response to ethanol in mice. Genes, brain, and behavior. 2013 Feb;12(1):87-97. PubMed PMID: 22883308.
2. Wiegert JS, Oertner TG. Long-term depression triggers the selective elimination of weakly integrated synapses. Proc Natl Acad Sci U S A. 2013 Nov 19;110(47):E4510-9. PubMed PMID: 24191047. Pubmed Central PMCID: 3839749.
3. de Anda FC, Rosario AL, Durak O, Tran T, Graff J, Meletis K, et al. Autism spectrum disorder susceptibility gene TAOK2 affects basal dendrite formation in the neocortex. Nat Neurosci. 2012 Jun 10;15(7):1022-31. PubMed PMID: 22683681. Pubmed Central PMCID: 4017029.
4. Riedl J, Crevenna AH, Kessenbrock K, Yu JH, Neukirchen D, Bista M, et al. Lifeact: a versatile marker to visualize F-actin. Nature methods. 2008 Jul;5(7):605-7. PubMed PMID: 18536722. Pubmed Central PMCID: 2814344.
5. Riedl J, Flynn KC, Raducanu A, Gartner F, Beck G, Bosl M, et al. Lifeact mice for studying F-actin dynamics. Nature methods. 2010 Mar;7(3):168-9. PubMed PMID: 20195247.
6. Noren NK, Pasquale EB. Eph receptor-ephrin bidirectional signals that target Ras and Rho proteins. Cellular signalling. 2004 Jun;16(6):655-66. PubMed PMID: 15093606.

7. Bock M, Umathum R, Zuehlsdorff S, Volz S, Fink C, Hallscheidt P, et al. Interventional magnetic resonance imaging: an alternative to image guidance with ionising radiation. Radiation protection dosimetry. 2005;117(1-3):74-8. PubMed PMID: 16464829.

8. Nieman BJ, Flenniken AM, Adamson SL, Henkelman RM, Sled JG. Anatomical phenotyping in the brain and skull of a mutant mouse by magnetic resonance imaging and computed tomography. Physiological genomics. 2006 Jan 12;24(2):154-62. PubMed PMID: 16410543.

9. Lerch JP, Pruessner J, Zijdenbos AP, Collins DL, Teipel SJ, Hampel H, et al. Automated cortical thickness measurements from MRI can accurately separate Alzheimer's patients from normal elderly controls. Neurobiology of aging. 2008 Jan;29(1):23-30. PubMed PMID: 17097767.

10. Genovese CR, Lazar NA, Nichols T. Thresholding of statistical maps in functional neuroimaging using the false discovery rate. NeuroImage. 2002 Apr;15(4):870-8. PubMed PMID: 11906227.

11. Andersson JL, Sotiropoulos SN. An integrated approach to correction for off-resonance effects and subject movement in diffusion MR imaging. NeuroImage. 2016 Jan 15;125:1063-78. PubMed PMID: 26481672. Pubmed Central PMCID: 4692656.

12. Behrens TE, Woolrich MW, Jenkinson M, Johansen-Berg H, Nunes RG, Clare S, et al. Characterization and propagation of uncertainty in diffusion-weighted MR imaging. Magnetic resonance in medicine. 2003 Nov;50(5):1077-88. PubMed PMID: 14587019.

13. Rorden C, Karnath HO, Bonilha L. Improving lesion-symptom mapping. Journal of cognitive neuroscience. 2007 Jul;19(7):1081-8. PubMed PMID: 17583985.

14. Jenkinson M, Bannister P, Brady M, Smith S. Improved optimization for the robust and accurate linear registration and motion correction of brain images. NeuroImage. 2002 Oct;17(2):825-41. PubMed PMID: 12377157.

15. Hartung H, Cichon N, De Feo V, Riemann S, Schildt S, Lindemann C, et al. From Shortage to Surge: A Developmental Switch in Hippocampal-Prefrontal Coupling in a Gene-Environment Model of Neuropsychiatric Disorders. Cereb Cortex. 2016 Sep 09. PubMed PMID: 27613435. Pubmed Central PMCID: 5066837.

16. Cichon NB, Denker M, Grun S, Hanganu-Opatz IL. Unsupervised classification of neocortical activity patterns in neonatal and pre-juvenile rodents. Frontiers in neural circuits. 2014;8:50. PubMed PMID: 24904296. Pubmed Central PMCID: 4034041.

17. Kwan V, Meka DP, White SH, Hung CL, Holzapfel NT, Walker S, et al. DIXDC1 Phosphorylation and Control of Dendritic Morphology Are Impaired by Rare Genetic Variants. Cell reports. 2016 Nov 08;17(7):1892-904. PubMed PMID: 27829159.

18. Freitag S, Schachner M, Morellini F. Behavioral alterations in mice deficient for the extracellular matrix glycoprotein tenascin-R. Behavioural brain research. 2003 Oct 17;145(1-2):189-207. PubMed PMID: 14529817.

19. Morellini F. Spatial memory tasks in rodents: what do they model? Cell and tissue research. 2013 Oct;354(1):273-86. PubMed PMID: 23793547.

20. Dail M, Richter M, Godement P, Pasquale EB. Eph receptors inactivate R-Ras through different mechanisms to achieve cell repulsion. Journal of cell science. 2006 Apr 01;119(Pt 7):1244-54. PubMed PMID: 16522685.

21. Richter M, Murai KK, Bourgin C, Pak DT, Pasquale EB. The EphA4 receptor regulates neuronal morphology through SPAR-mediated inactivation of Rap GTPases. J Neurosci. 2007 Dec 19;27(51):14205-15. PubMed PMID: 18094260.
22. Franklin KBJ, Paxinos, G. The mouse brain in stereotaxic coordinates. New York: Academic Press. 2001.
23. Koster JD, Leggewie B, Blechner C, Brandt N, Fester L, Rune G, et al. Inositol-1,4,5-trisphosphate-3-kinase-A controls morphology of hippocampal dendritic spines. Cellular signalling. 2016 Jan;28(1):83-90. PubMed PMID: 26519023.
24. Gray EG. Axo-somatic and axo-dendritic synapses of the cerebral cortex: an electron microscope study. Journal of anatomy. 1959 Oct;93:420-33. PubMed PMID: 13829103. Pubmed Central PMCID: 1244535.
25. Gray EG. Electron microscopy of synaptic contacts on dendrite spines of the cerebral cortex. Nature. 1959 Jun 06;183(4675):1592-3. PubMed PMID: 13666826.
26. DeFelipe J. The dendritic spine story: an intriguing process of discovery. Front Neuroanat. 2015;9:14. PubMed PMID: 25798090. Pubmed Central PMCID: 4350409.
27. Pellegrin S, Mellor H. Rho GTPase activation assays. Current protocols in cell biology. 2008 Mar;Chapter 14:Unit 14 8. PubMed PMID: 18360815.
28. Hui-Yuen J, McAllister S, Koganti S, Hill E, Bhaduri-McIntosh S. Establishment of Epstein-Barr virus growth-transformed lymphoblastoid cell lines. Journal of visualized experiments : JoVE. 2011 Nov 08(57). PubMed PMID: 22090023. Pubmed Central PMCID: 3308597.
29. Gage GJ, Kipke DR, Shain W. Whole animal perfusion fixation for rodents. Journal of visualized experiments : JoVE. 2012 Jul 30(65). PubMed PMID: 22871843. Pubmed Central PMCID: 3476408.
30. Phansalskar N, More, S., Sabale, A. . Adaptive local thresholding for detection of nuclei in diversity stained cytology images. International Conference on Communications and Signal Processing (ICCSP). 2011:218-20.
31. Longair MH, Baker DA, Armstrong JD. Simple Neurite Tracer: open source software for reconstruction, visualization and analysis of neuronal processes. Bioinformatics. 2011 Sep 01;27(17):2453-4. PubMed PMID: 21727141.
32. Ferreira TA, Blackman AV, Oyrer J, Jayabal S, Chung AJ, Watt AJ, et al. Neuronal morphometry directly from bitmap images. Nature methods. 2014 Oct;11(10):982-4. PubMed PMID: 25264773. Pubmed Central PMCID: 5271921.
33. Risher WC, Ustunkaya T, Singh Alvarado J, Eroglu C. Rapid Golgi analysis method for efficient and unbiased classification of dendritic spines. PloS one. 2014;9(9):e107591. PubMed PMID: 25208214. Pubmed Central PMCID: 4160288.
34. Wickham H. ggplot2: Elegant Graphics for Data Analysis. Springer-Verlag New York. 2009.
35. Tseng Q, Duchemin-Pelletier E, Deshiere A, Balland M, Guillou H, Filhol O, et al. Spatial organization of the extracellular matrix regulates cell-cell junction positioning. Proc Natl Acad Sci U S A. 2012 Jan 31;109(5):1506-11. PubMed PMID: 22307605. Pubmed Central PMCID: 3277177.
36. Tarnok K, Gulyas M, Bencsik N, Ferenc K, Pfizenmaier K, Hausser A, et al. A new tool for the quantitative analysis of dendritic filopodial motility. Cytometry Part A : the journal of the International Society for Analytical Cytology. 2015 Jan;87(1):89-96. PubMed PMID: 25257846.

Supplementary Figure 1

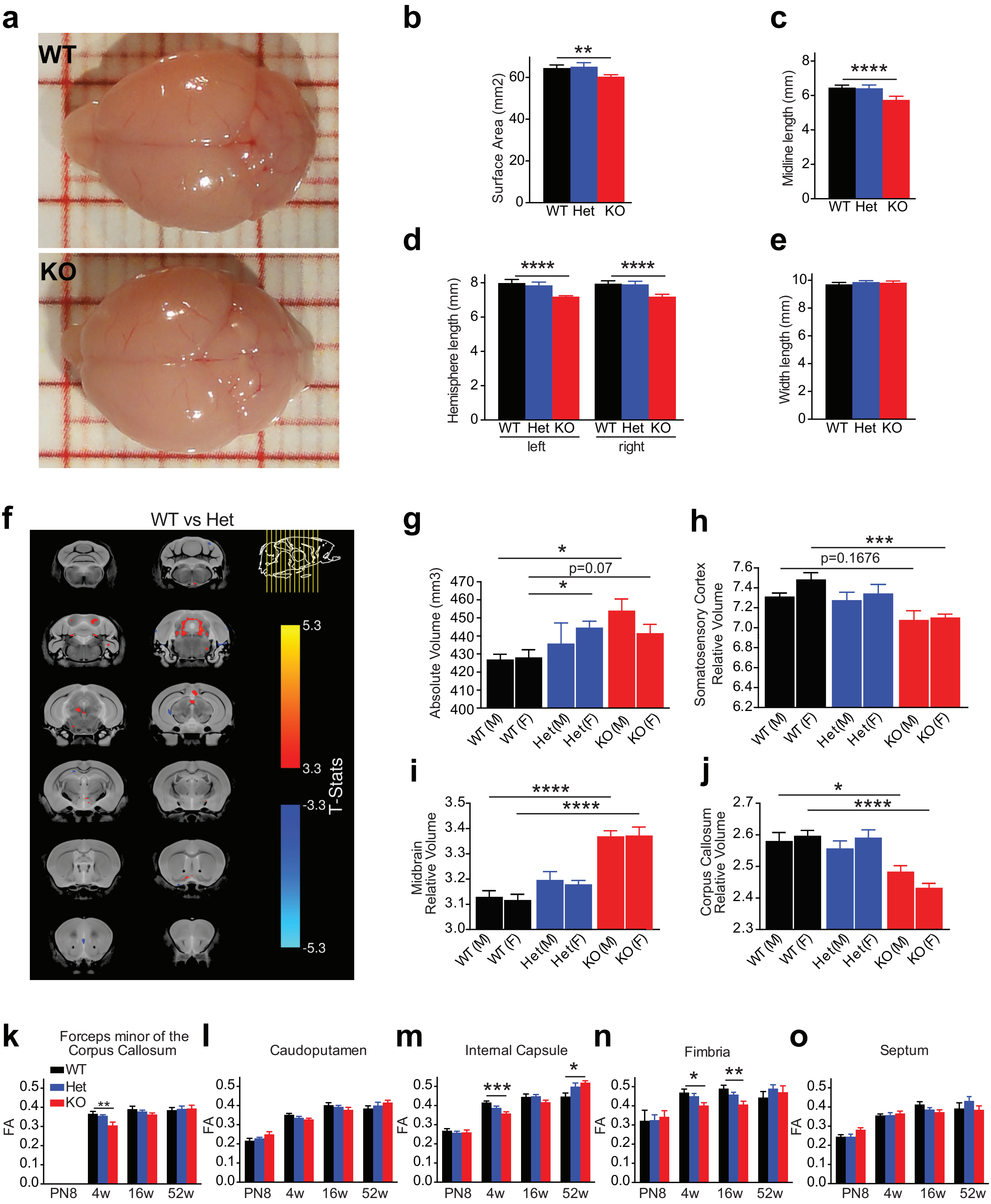

Supplementary Figure 2

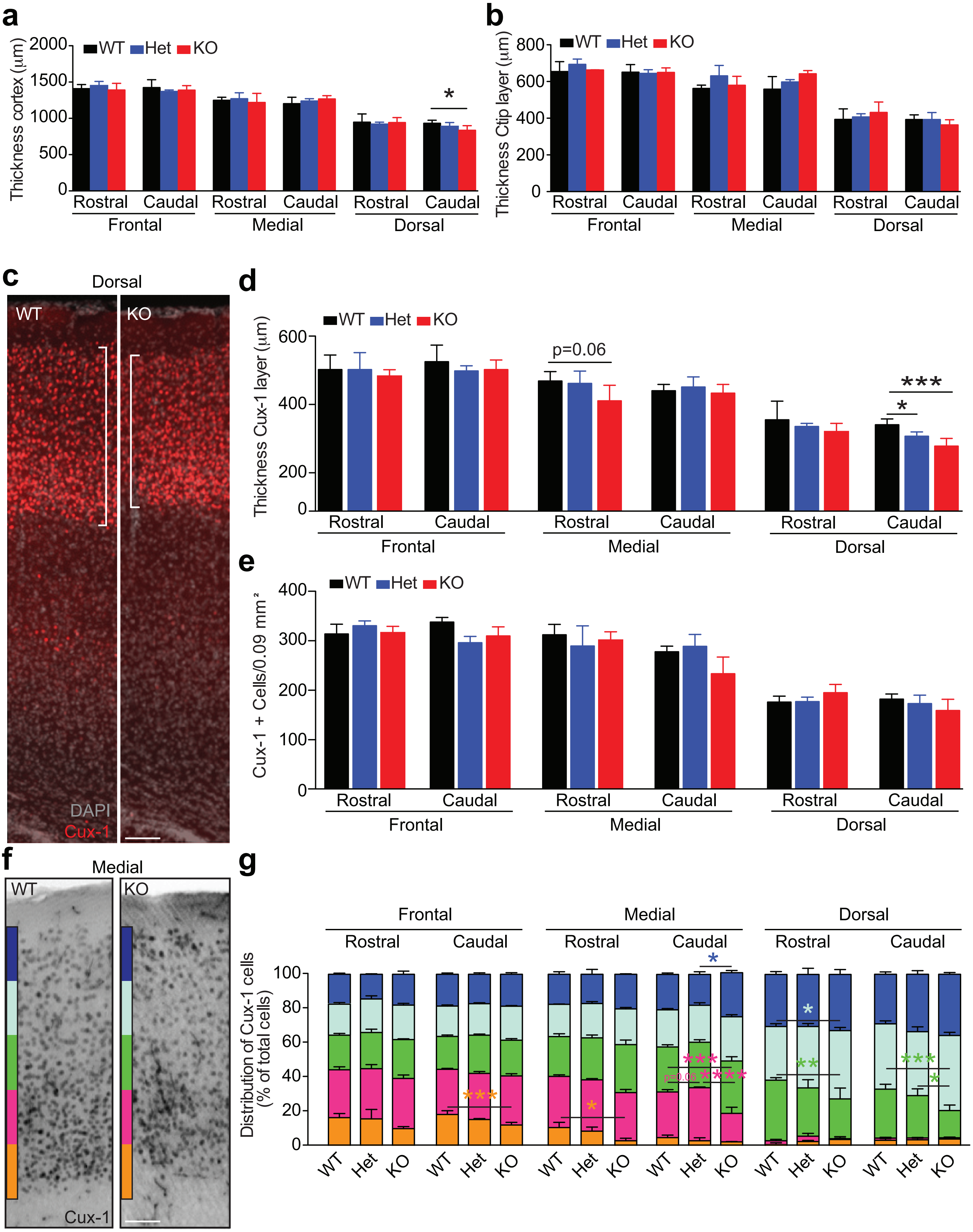

Supplementary Figure 3

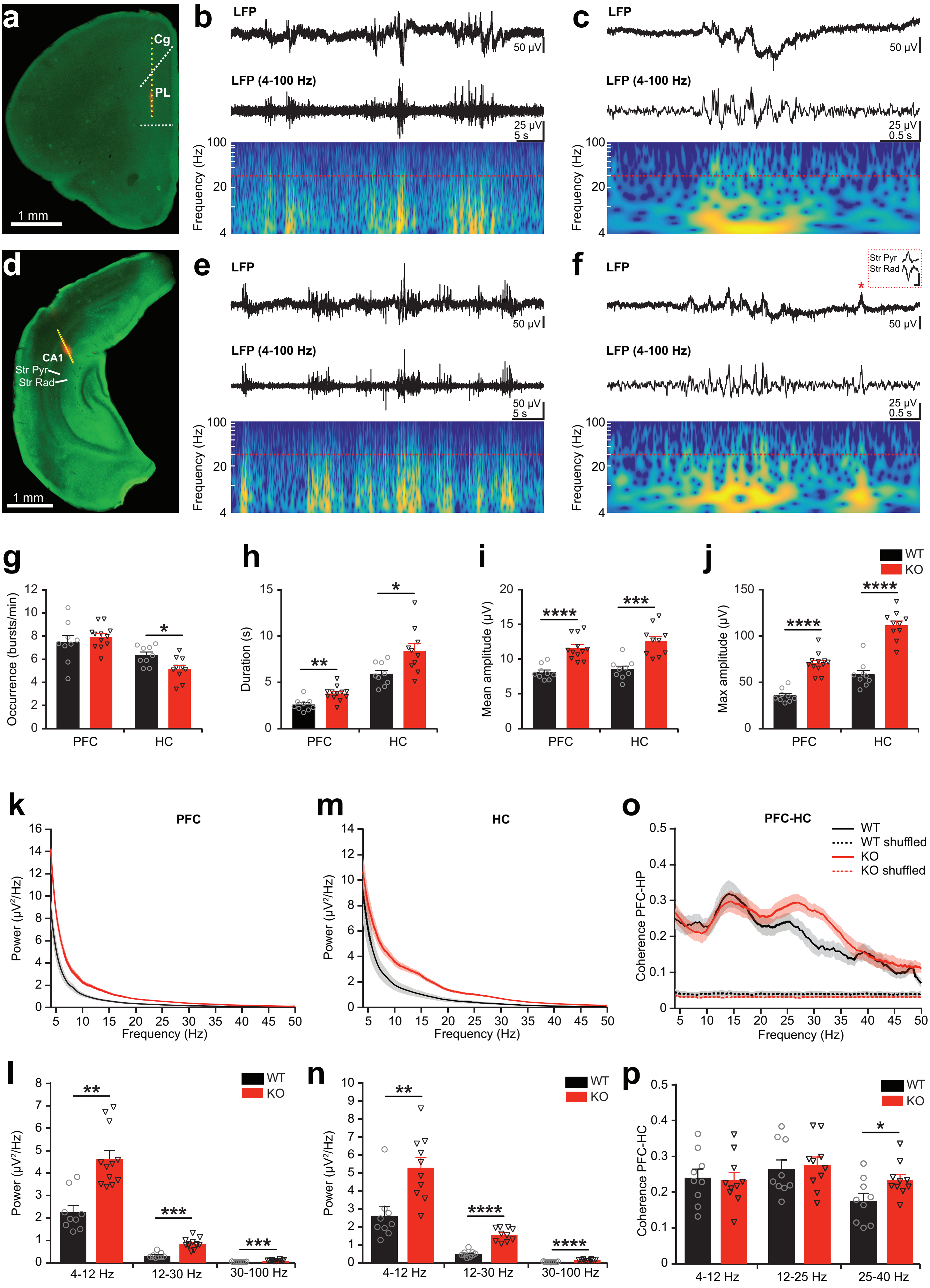

Supplementary Table 2

Mean prefrontal-hippocampal coherence in different frequency bands as well as properties of discontinuous patterns of oscillatory activity in PFC and HC of neonatal control and TAOK2 KO mice. Data are shown as mean ± s.e.m. Significance levels for comparisons between controls and TAOK2 KO mice are listed.

		Controls (n=9)	TAOK2 KO (n=10-12)	p value
PFC-HC	Coherence (4-12 Hz)	0.240 ± 0.025	0.233 ± 0.022	0.838
	Coherence (12-25 Hz)	0.265 ± 0.025	0.276 ± 0.022	0.746
	Coherence (25-40 Hz)	0.176 ± 0.021	0.234 ± 0.015	0.035
PFC	Occurrence (bursts/min)	7.45 ± 0.59	7.93 ± 0.29	0.442
	Duration (s)	2.57 ± 0.22	3.78 ± 0.22	0.001
	Mean amplitude (µV)	8.10 ± 0.34	11.58 ± 0.49	0.00003
	Maximum amplitude (µV)	35.54 ± 2.36	70.46 ± 3.20	$1.07 * 10^{-7}$
	Power 4-12 Hz (μV^2/Hz)	2.26 ± 0.29	4.63 ± 0.37	0.001
	Power 12-30 Hz (μV^2/Hz)	0.33 ± 0.04	0.86 ± 0.07	0.0002
	Power 30-100 Hz (μV^2/Hz)	0.04 ± 0.003	0.10 ± 0.01	0.0001
HC	Occurrence (bursts/min)	6.34 ± 0.28	5.15 ± 0.33	0.014
	Duration (s)	5.88 ± 0.41	8.39 ± 0.78	0.013
	Mean amplitude (µV)	8.52 ± 0.47	12.61 ± 0.68	0.0001
	Maximum amplitude (µV)	58.39 ± 4.61	111.52 ± 4.93	$5.02 * 10^{-7}$
	Power 4-12 Hz (μV^2/Hz)	2.62 ± 0.50	5.30 ± 0.57	0.003
	Power 12-30 Hz (μV^2/Hz)	0.49 ± 0.06	1.57 ± 0.12	$3.99 * 10^{-7}$
	Power 30-100 Hz (μV^2/Hz)	0.05 ± 0.01	0.15 ± 0.01	$3.99 * 10^{-7}$

Supplementary Figure 4

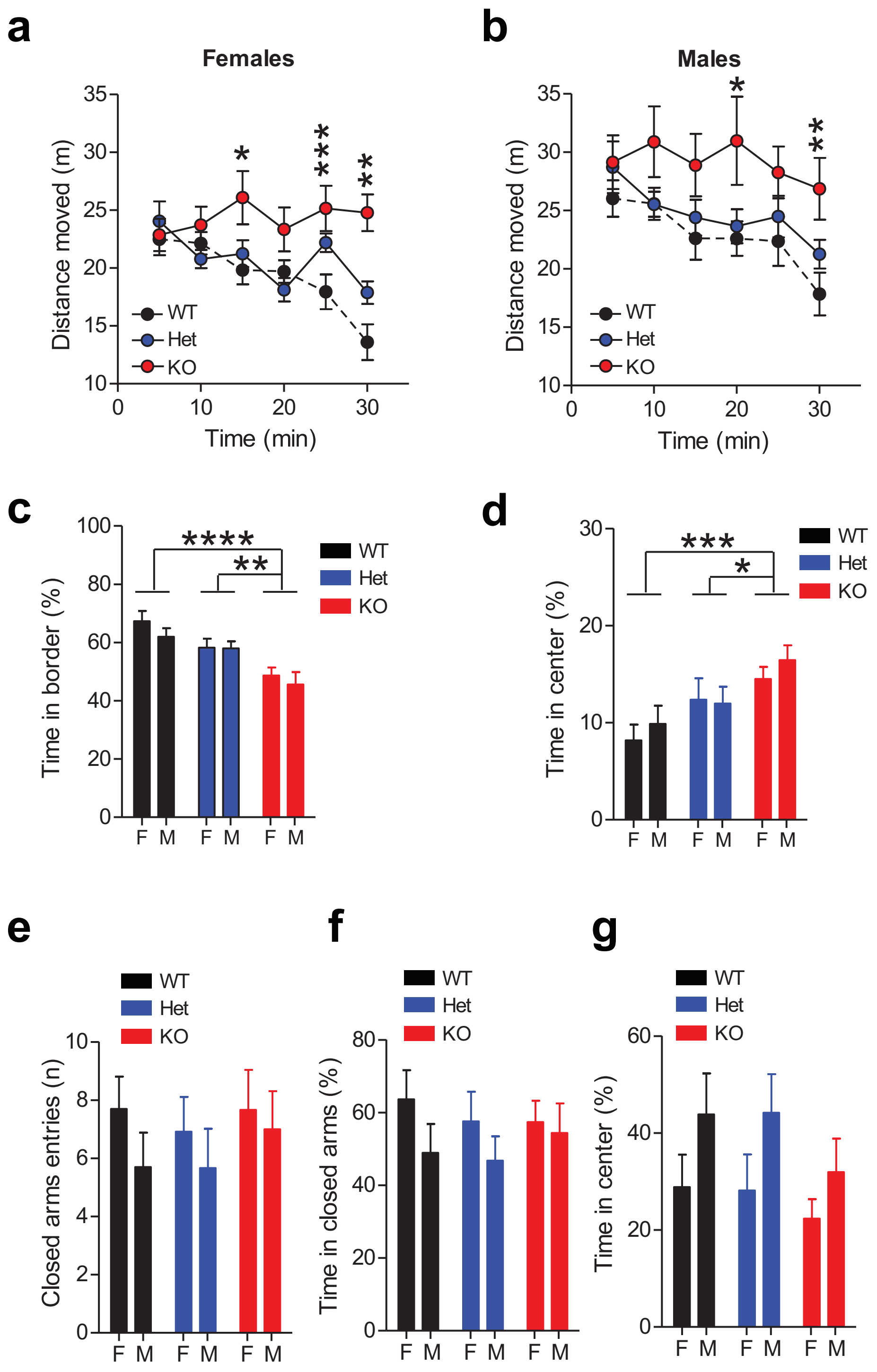

Supplementary Figure 5

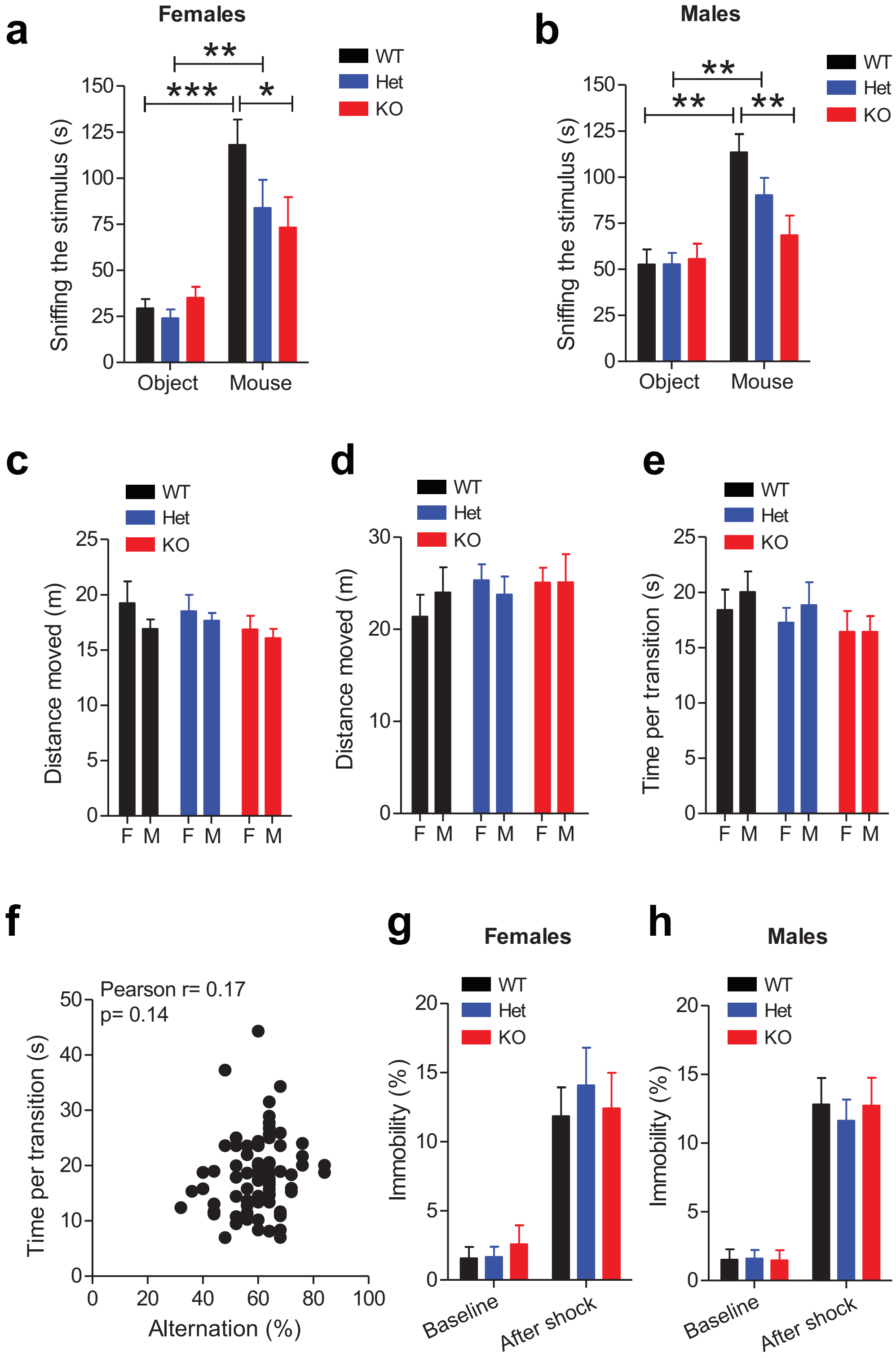

Supplementary Figure 6

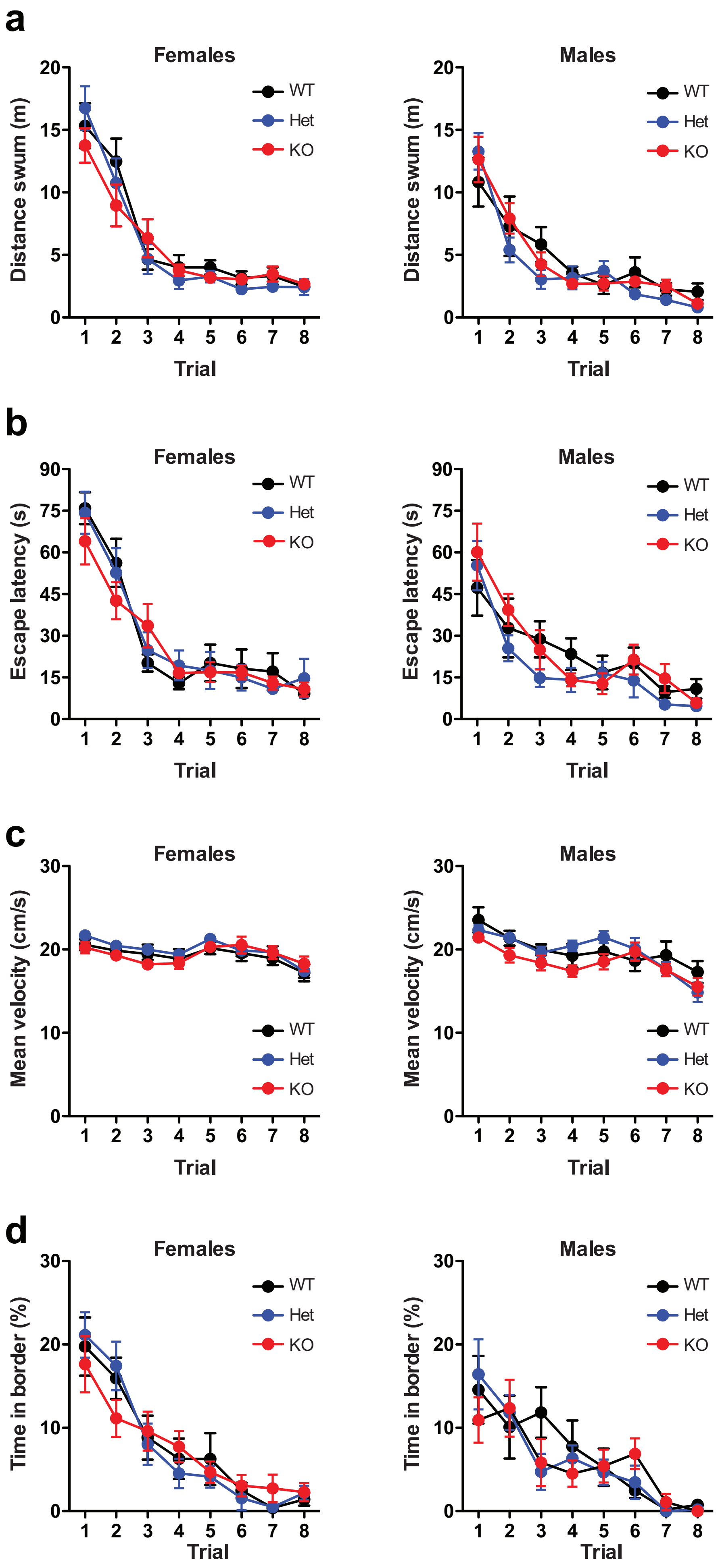

Supplementary Figure 7

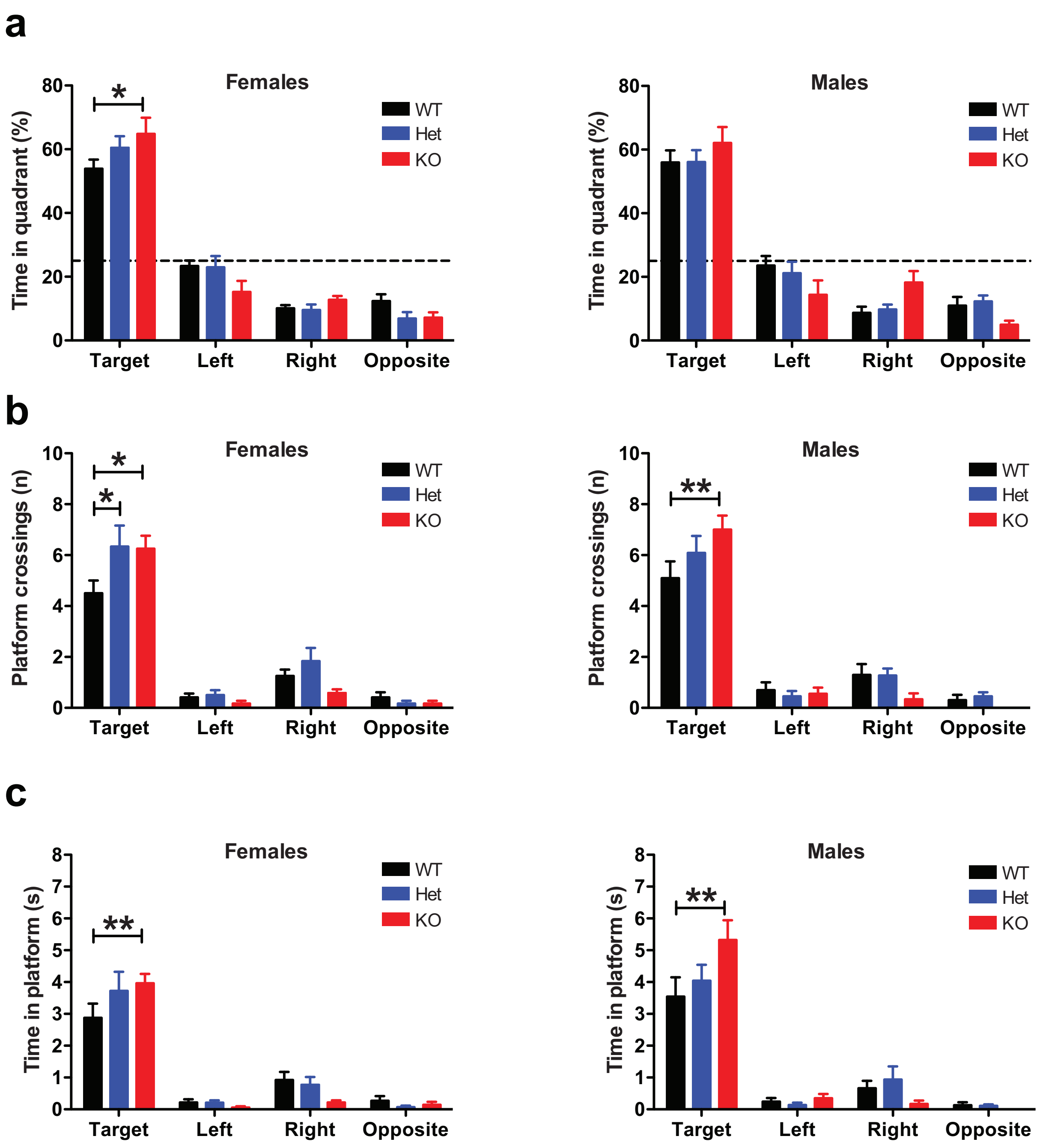

Supplementary Figure 8

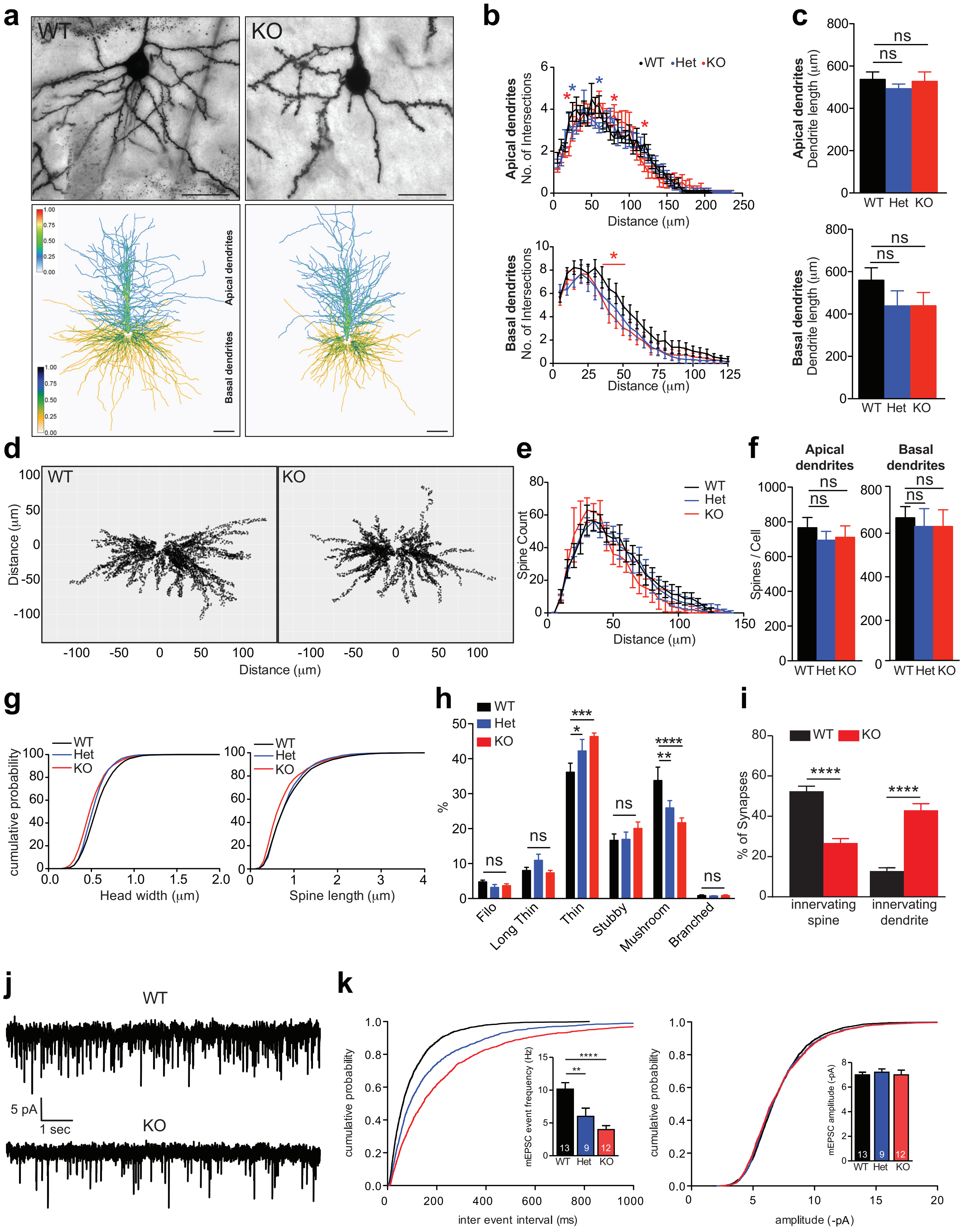

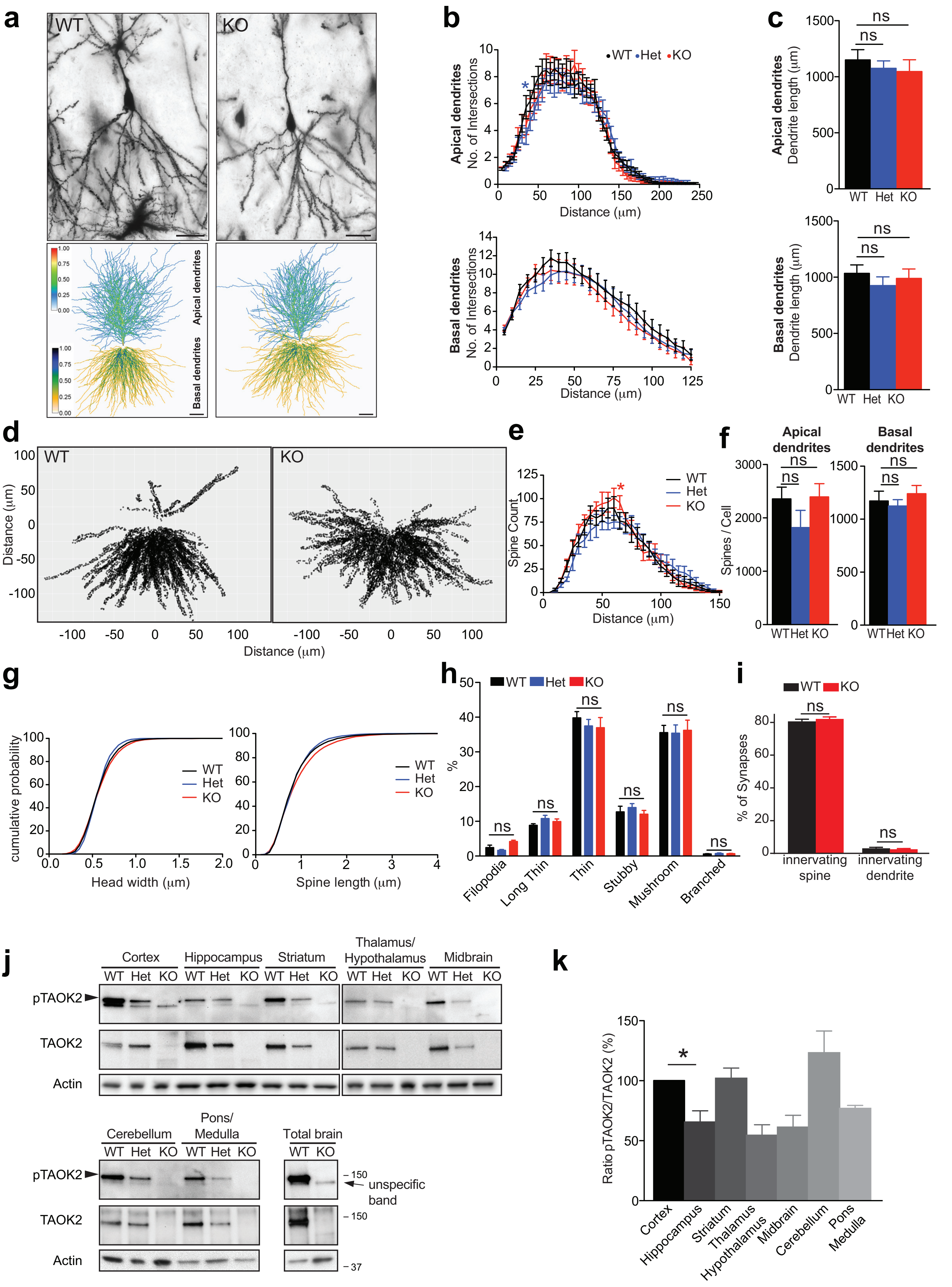
a
WT
KO
Apical dendrites
Basal dendrites
b
Apical dendrites
No. of Intersections
WT Het KO
Distance (µm)
Basal dendrites
No. of Intersections
c
Apical dendrites
Dendrite length (µm)
ns
WT Het KO
Basal dendrites
Dendrite length (µm)
d
WT
KO
Distance (µm)
e
Spine Count
WT
Het
KO
f
Apical dendrites
Basal dendrites
Spines / Cell
g
cumulative probability
Head width (µm)
Spine length (µm)
h
%
Filopodia
Long Thin
Thin
Stubby
Mushroom
Branched
i
% of Synapses
innervating spine
innervating dendrite
j
Cortex
Hippocampus
Striatum
Thalamus/ Hypothalamus
Midbrain
pTAOK2
TAOK2
Actin
Cerebellum
Pons/ Medulla
Total brain
150
unspecific band
37
k
Ratio pTAOK2/TAOK2 (%)
Cortex
Hippocampus
Striatum
Thalamus Hypothalamus
Midbrain
Cerebellum
Pons Medulla

Supplementary Figure 10

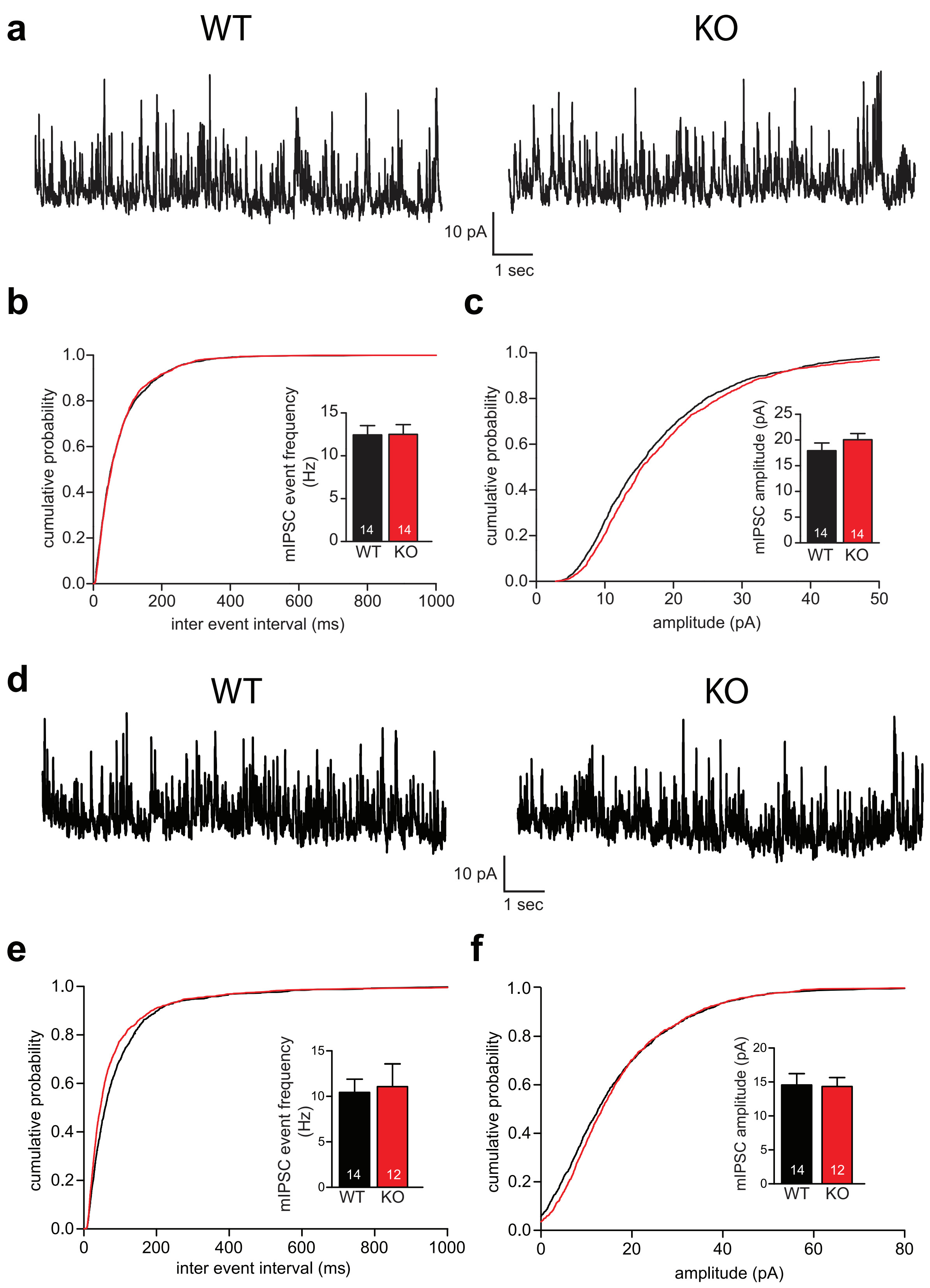

Supplementary Figure 11

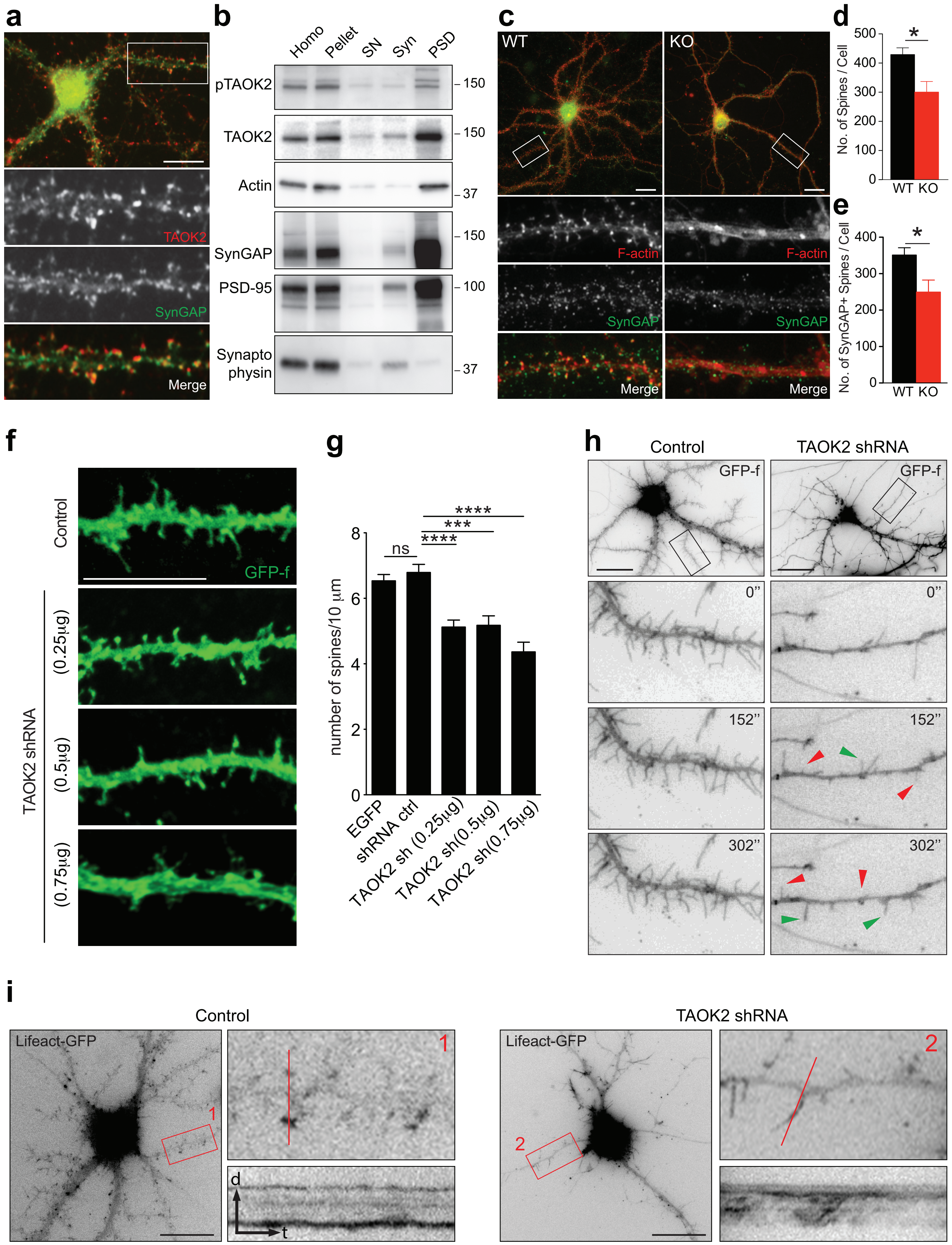

Supplementary Figure 12

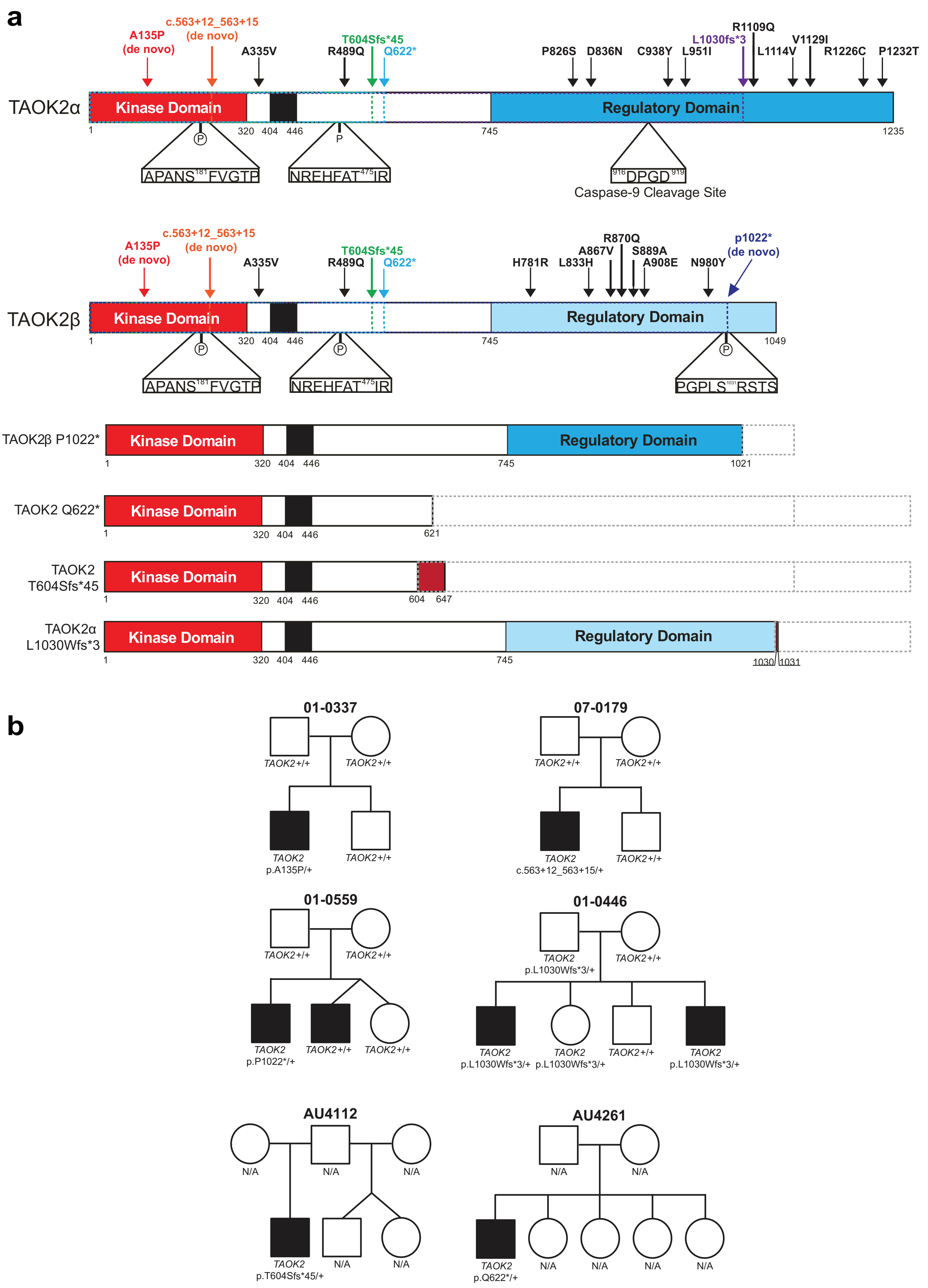

Supplementary Table 3

Position	Reference	Type	Genotype	RefSeq Effect	RefSeq Codon Change	Amino Acid Change	RefSeq Transcript	Affected Isoforms	Inheritance	Minor Allele Frequency (%)	Polyphen Score
29990345	G	HET	G/C	MISSENSE	Gcg/Ccg	A135P	NM_016151	Alpha Beta Gamma	***de novo***	-	**0.97**
30002803	GGGCCCCCCTGC TGCCGCGGTGCCC	HET	GGGCCCCCCTGC TGCCGCGGTGCCC/--	FRAMESHIFT DELETION	-	P1022*	NM_004783	Beta	***de novo***	-	**N/A**
29990629	GTGA	HET	GTGA/--	SPLICE SITE DELETION	N/A	c.563+12_563+15	NC_000016.9	Alpha Beta Gamma	***de novo***	-	**N/A**
29996995	CCAGCACTCCCAAG CGGGAGAAGGCCG AGTGGCTGCTGCGG CAGAAGGAGCAGCT	HET	CCAGCACTCCCAAG CGGGAGAAGGCCG AGTGGCTGCTGCGG CAGAAGGAGCAGCT/--	FRAMESHIFT DELETION	-	T604Sfs*45	NM_016151	Alpha Beta Gamma	Unknown	-	**N/A**
29997054	C	HET	C/T	NONSENSE	Cag/Tag	Q622*	NM_016151	Alpha Beta Gamma	Unknown	-	**N/A**
29998680	C	HET	C/--	FRAMESHIFT DELETION	-	L1030Wfs*3	NM_016151	Alpha Gamma	Paternal<br>Present in unaffected and affected sibling	-	**N/A**
29994397	C	HET	C/T	MISSENSE	gCc/gTc	A335V	NM_016151	Alpha Beta Gamma	Paternal	-	**.008**
29996577	G	HET	G/A	MISSENSE	cGg/cAg	R489Q	NM_016151	Alpha Beta Gamma	Paternal from paternal grandmother<br>Not present in affected sibling	-	**.999**
29998069	C	HET	C/T	MISSENSE	Ccc/Tcc	P826S	NM_016151	Alpha	Paternal	-	**0.002**
29998099	G	HET	G/A	MISSENSE	Gat/Aat	D836N	NM_016151	Alpha	Paternal	-	**0.001**
29998406	G	HET	G/A	MISSENSE	tGa/tAc	C938Y	NM_016151	Alpha Gamma	Paternal	-	**0**
29998444	C	HET	C/A	MISSENSE	Ctc/Atc	L951I	NM_016151	Alpha Gamma	Paternal<br>Not present in unaffected sibling	-	**.996**
29998919	G	HET	G/A	MISSENSE	cGg/cAg	R1109Q	NM_016151	Alpha Gamma	Maternal	-	**0.034**
29998933	C	HET	C/G	MISSENSE	Ctg/Gtg	L1114V	NM_016151	Alpha Gamma	Maternal<br>Present in affected sibling	-	**0.546**
29998978	G	HET	G/A	MISSENSE	Gtc/Atc	V1129I	NM_016151	Alpha Gamma	Maternal<br>Present in affected sibling	-	**0.002**
29999269	C	HET	C/T	MISSENSE	Cgc/Tgc	R1226C	NM_016151	Alpha Gamma	Maternal<br>Present in affected sibling	-	**.988**
29999287	C	HET	C/A	MISSENSE	Ccc/Acc	P1232T	NM_016151	Alpha Gamma	Maternal<br>Present in affected sibling	-	**.996**
30001023	A	HET	A/G	MISSENSE	cAc/cGc	H781R	NM_004783	Beta	Maternal	0.0539%	**.998**
30002157	T	HET	T/A	MISSENSE	cTt/cAt	L833H	NM_004783	Beta	Unknown	-	**1**
30002259	C	HET	C/T	MISSENSE	gCg/gTg	A867V	NM_004783	Beta	Paternal	-	**.07**
30002268	G	HET	G/A	MISSENSE	cGg/cAg	R870Q	NM_004783	Beta	Maternal	0.04%	**.998**
30002404	T	HET	T/G	MISSENSE	Tcc/Gcc	S889A	NM_004783	Beta	Maternal	-	**.311**
30002462	C	HET	C/A	MISSENSE	gCg/gAg	A908E	NM_004783	Beta	Not Maternal<br>No paternal DNA	-	**.271**
30002677	A	HET	A/T	MISSENSE	Aac/Tac	N980Y	NM_004783	Beta	Maternal	-	**.056**

red: *de novo* mutations; dark red: variants resulting in truncations; black: rare-inherited variants found in all isoforms; green: rare-inherited variants affecting alpha isoform only; blue: rare-inherited varaints affecting alpha and gamma isoforms; purple: rare-inherited variants affecting beta isoform only

Supplementary Figure 13

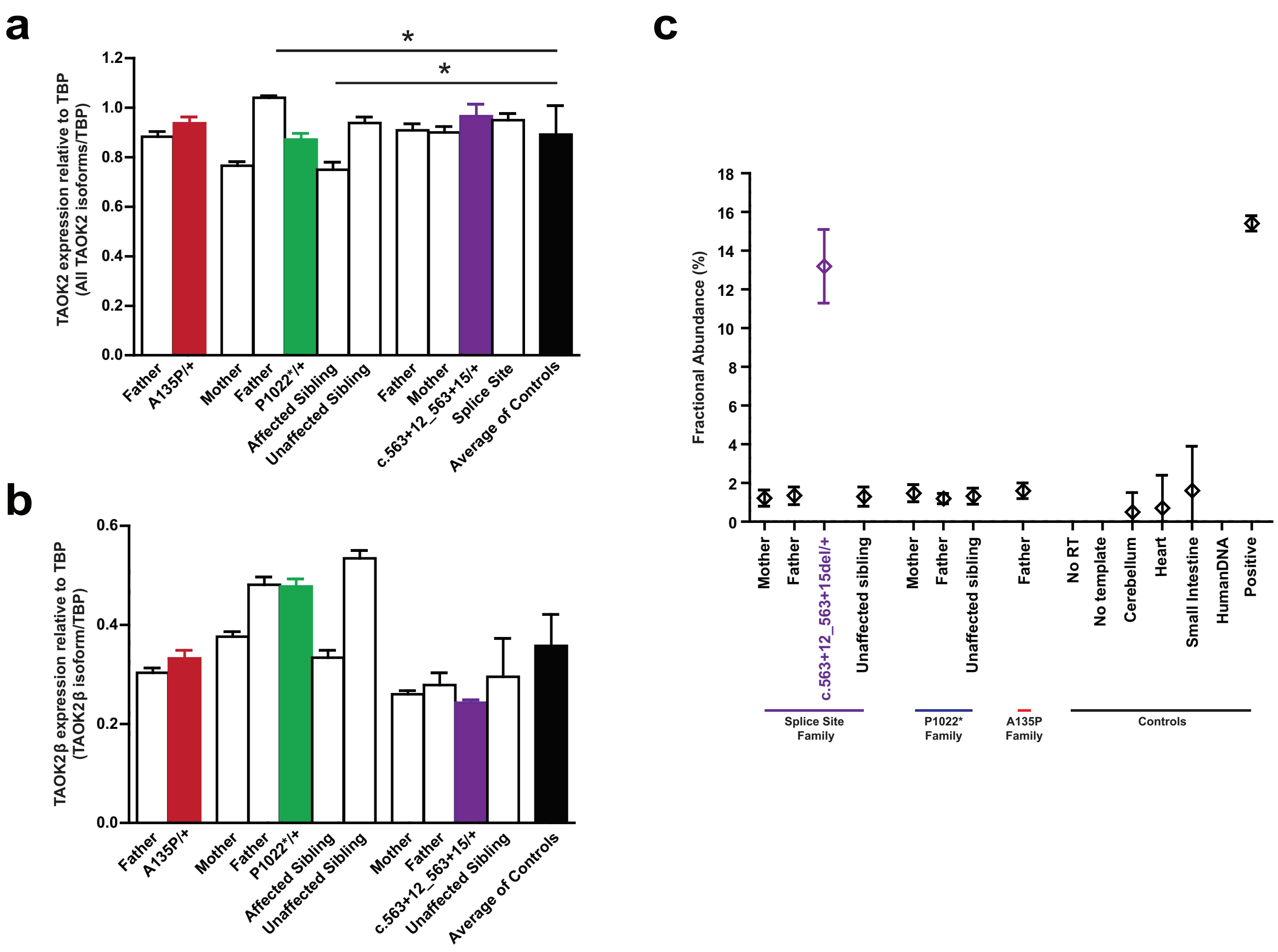

Supplementary Figure 14

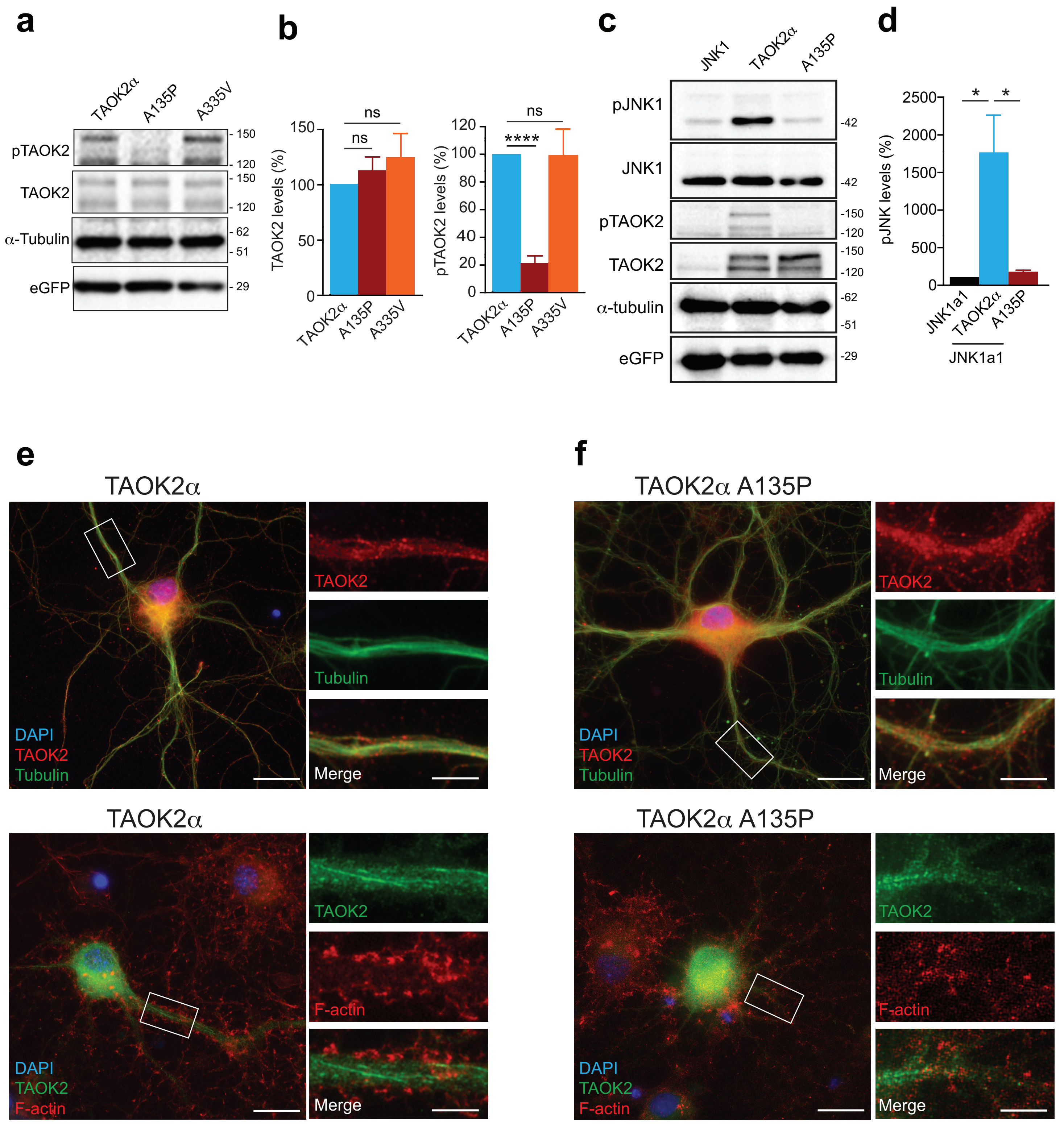

Supplementary Figure 15

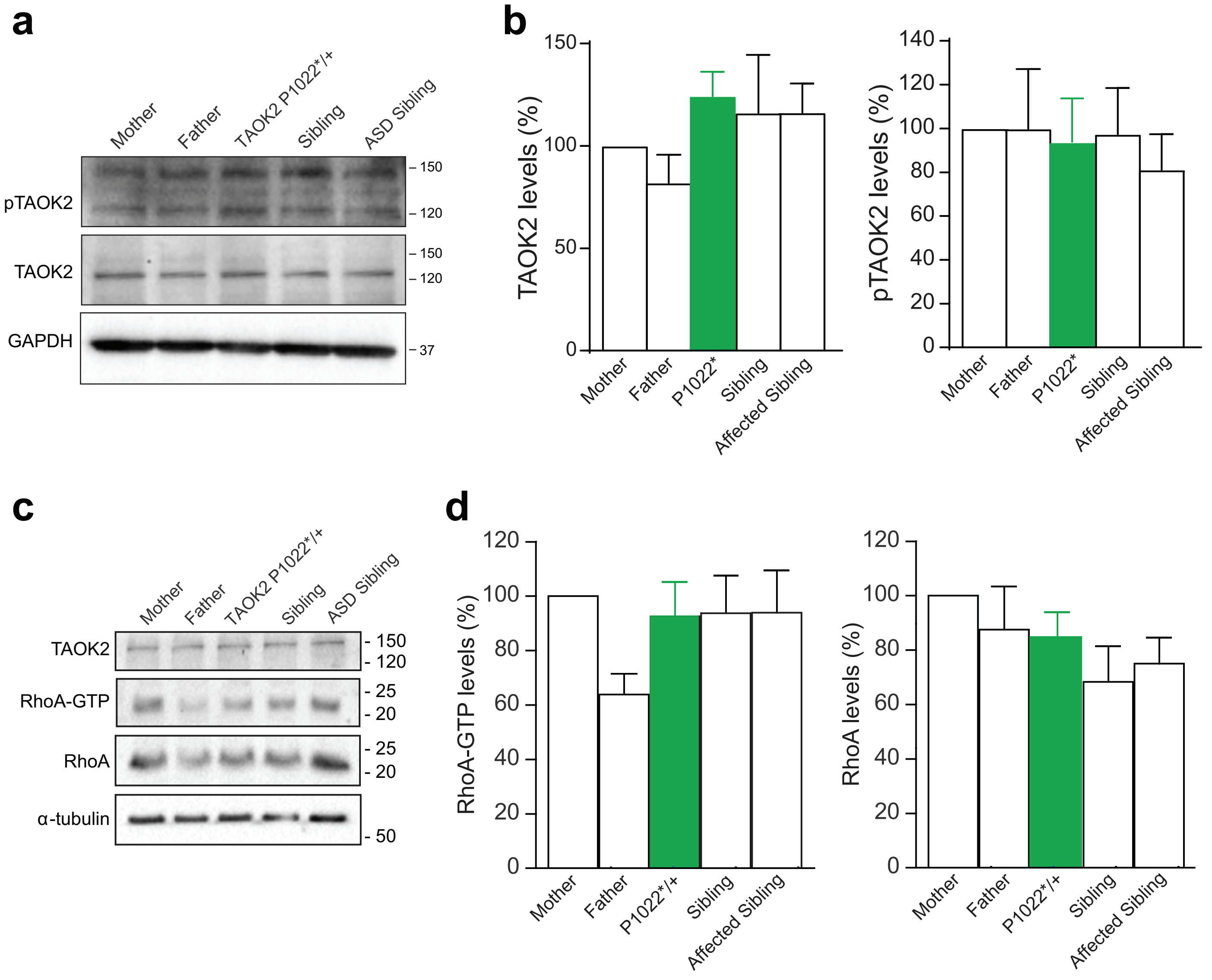

Supplementary Figure 16

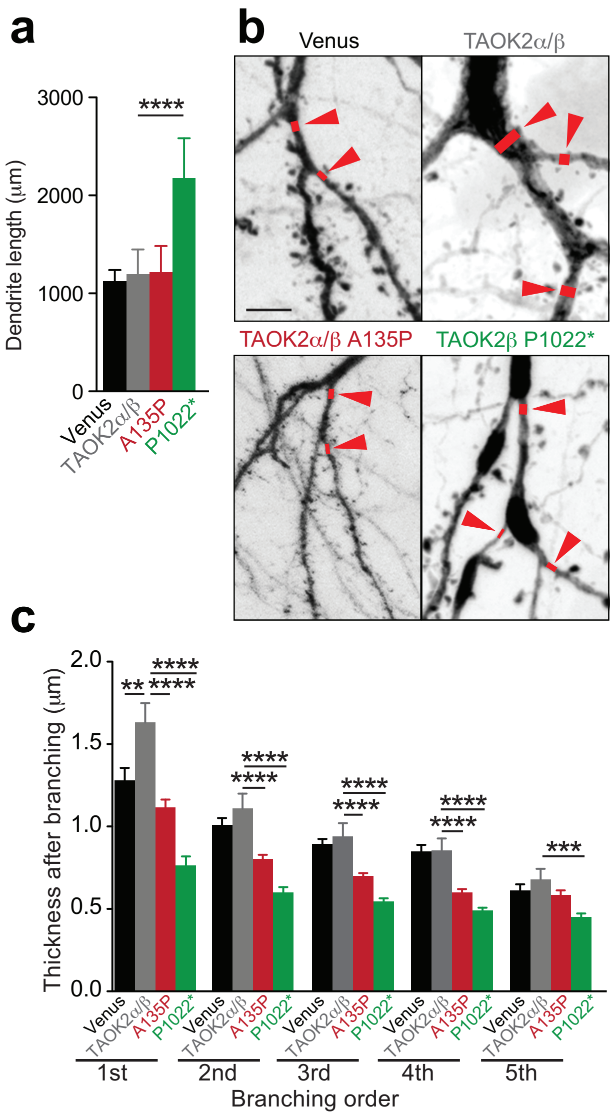

Supplementary Figure 17

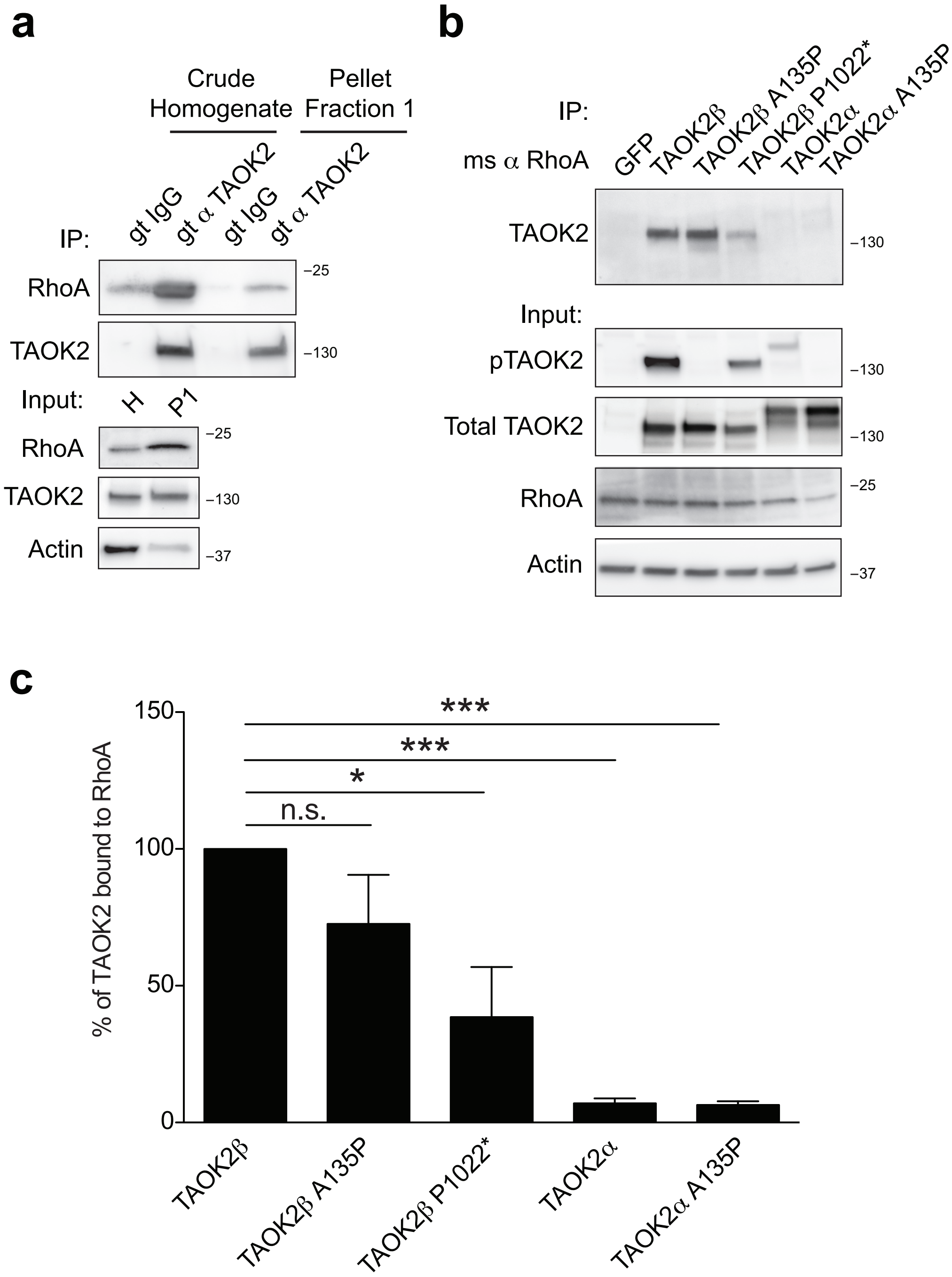

Supplementary Figure 18

a

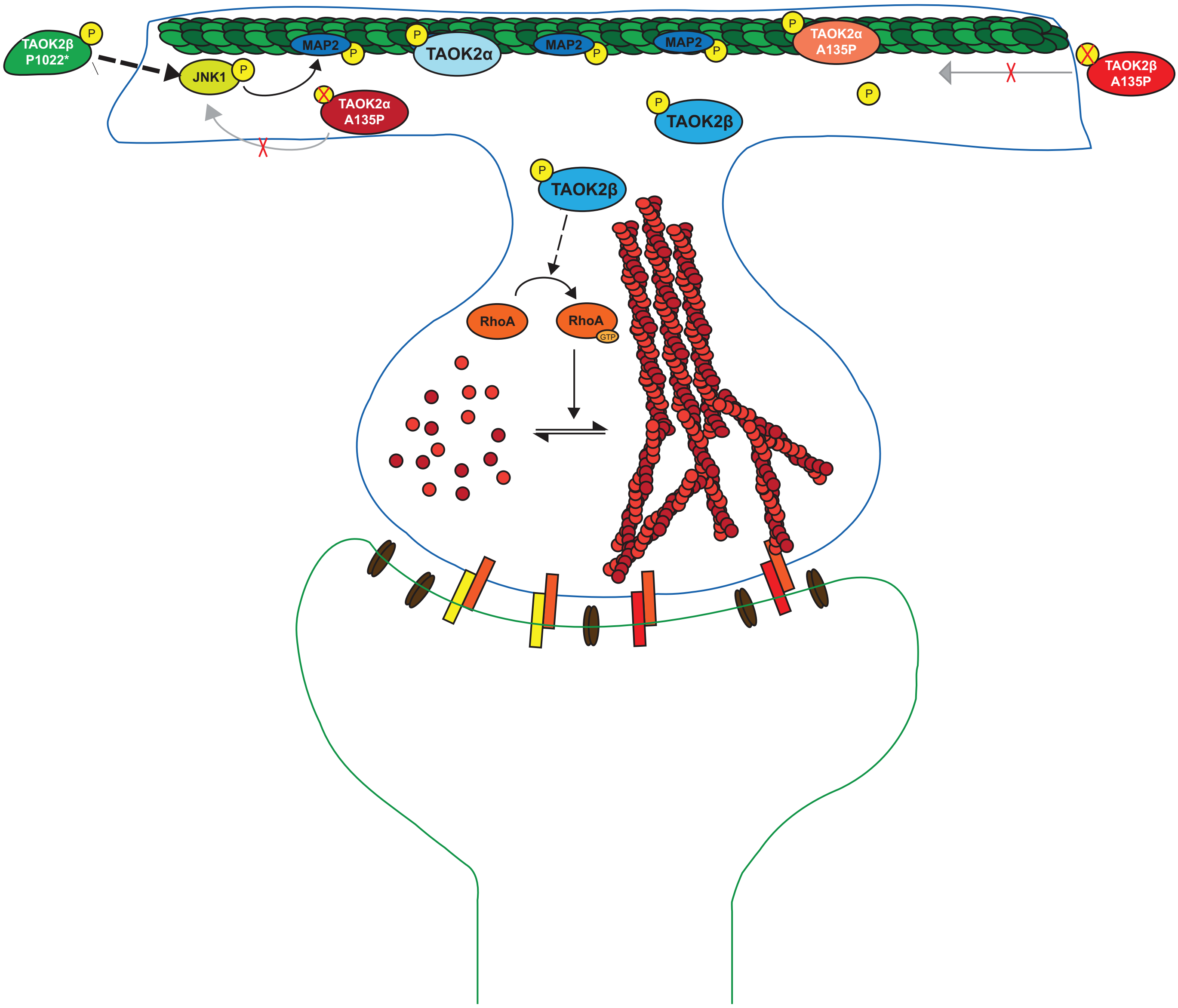

b

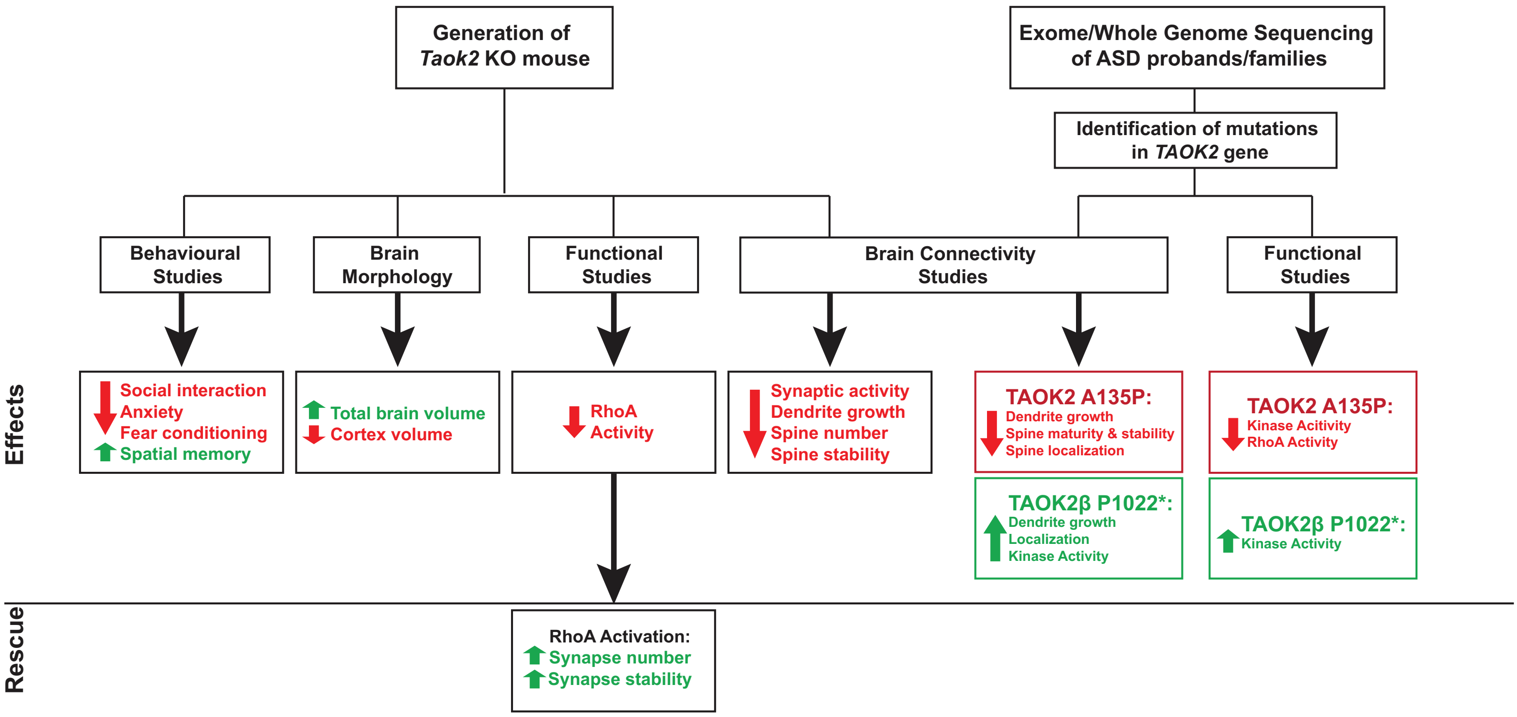

Supplementary Figure 1. ***Taok2*** **KO mice have altered brain morphology.** (a) Top view of WT and *Taok2* KO mouse brains. (b-e) The cortex of *Taok2* KO mice have decreased surface area (mm^2), midline length (mm) and bilateral hemisphere lengths (mm), with no change in width (WT=6, Het=7 and KO=5 brains from 3 different litters; Surface Area: one-way ANOVA, *post hoc* Dunnett's test; F(2,15)=14.49, p=0.0003; WT vs KO p=0.0010; Midline length: one-way ANOVA, *post hoc* Dunnett's test; F(2,15)=23.54, p<0.0001; WT vs KO p<0.0001; Left Hemisphere length: F(2,15)=32.01, p<0.0001; WT vs KO p<0.0001; Right Hemisphere length: F(2,15)=32.01, p<0.0001; WT vs KO p<0.0001; Width length: one-way ANOVA, *post hoc* Dunnett's test F(2,15)=2.942, p=0.0836). (f) Voxel-wise analysis highlighting significant relative volume differences (images show the lowest threshold of 20% false discovery rate for *Taok2* Het mice, FDR) throughout the brain between the WT and *Taok2* Het mice. T-stats of 3.3 to 5.3 indicates decreasing false discovery rate, where 3.3 = 20% FDR and positive or negative T-stat indicates positive or negative change compared to WT brain. (g) *Taok2* KO mice and female *Taok2* Het have increased absolute brain volume compared to WT mice (WT(M)=4, WT(F)=10, Het(M)=5, Het(F)=8, KO(M)=11, KO(F)=12 mice from 3 different cohorts, statistics by linear model corrected for multiple comparisons using FDR; WT(M) vs KO (M) p=0.0127, WT(F) vs KO (F) p=0.0711, WT(M) vs Het(M) p=0.4497, WT(F) vs Het(F) p=0.0167). (h) *Taok2* KO female mice have decreased relative volume of the somatosensory cortex compared to WT mice, with male mice also trending towards a decrease (WT(M)=4, WT(F)=10, Het(M)=5, Het(F)=8, KO(M)=11, KO(F)=12 mice from 3 different cohorts, statistics by linear model corrected for multiple comparisons using FDR; WT(M) vs KO (M) p=0.1678, WT(F) vs KO (F) p=0.0009, WT(M) vs Het(M) p=0.4348, WT(F) vs Het(F) p=0.4293).(i) *Taok2* KO male and female mice show an increase in relative volume of the midbrain compared to WT mice (WT(M)=4, WT(F)=10, Het(M)=5, Het(F)=8, KO(M)=11, KO(F)=12 mice from 3 different cohorts, statistics by linear model corrected for multiple comparisons using FDR; WT(M) vs KO (M) p<0.0001, WT(F) vs KO (F) p<0.0001, WT(M) vs Het(M) p=0.8045, WT(F) vs Het(F) p=0.5753). (j) The relative volume of the corpus callosum is reduced in male and female *Taok2* KO mice (WT(M)=4, WT(F)=10, Het(M)=5, Het(F)=8, KO(M)=11, KO(F)=12 mice from 3 different cohorts, statistics by linear model corrected for multiple comparisons using FDR; WT(M) vs KO (M) p=0.0278, WT(F) vs KO (F) p<0.0001, WT(M) vs Het(M) p=0.9171, WT(F) vs Het(F) p=0.9375). (k-o) Diffusion tensor imaging reveal delayed development of the forceps minor of

the corpus callosum, internal capsule and fimbria in *Taok2* KO mice (WT=5-7, Het=6-15 and KO=6-11 brains from 3 different cohorts; forceps minor of the corpus callosum: one-way ANOVA, *post hoc* Dunnett's test; 4wks: F(2,29)=7.094, p=0.0031; WT vs KO p=0.0041; internal capsule: one-way ANOVA, *post hoc* Dunnett's test; 4wks: F(2,29)=8.225, p=0.0015; WT vs KO p=0.0008; 52wks: F(2,14)=5.377, p=0.0185; WT vs KO p=0.0117; fimbria: one-way ANOVA, *post hoc* Dunnett's test; 4wks: F(2,29)=4.832, p=0.0154; WT vs KO p=0.0131; 16wks: F(2,31)=6.113, p=0.0058; WT vs KO p=0.0044). *p<0.05, **p<0.01, ***p<0.001 and ****p<0.0001. Values are mean +/- s.e.m.

Supplementary Figure 2. Abnormal neuronal and cortical positioning after loss of Taok2 (a) Cortex thickness is decreased in *Taok2* KO compared with WT littermates (WT=5-9 brains, Het=3-6 brains, KO=2-6 brains; dorsal-caudal: one-way ANOVA, *post hoc* Dunnett's test F(2,13)=4.312, p=0.0366; WT vs KO p=0.0219). (b) Thickness of Ctip+ lower cortex is not altered in *Taok2* Het or KO cortices compared with WT littermates (WT=5-8 brains, Het=3-6 brains, KO=2-4 brains; one-way ANOVA, not significant in all regions). (c) Cux-1+ upper cortex is thinner in *Taok2* KO cortices. Scale bars represent 200μm. (d) Quantification of thickness of Cux-1+ upper cortex (WT=5-9 brains, Het=3-6 brains, KO=2-6 brains; medial-rostral: one-way ANOVA, *post hoc* Dunnett's test, F(2, 12)=3.03, p=0.0861; WT vs KO p= 0.0633; p=0.045 by t-test; dorsal-caudal: one-way ANOVA, *post hoc* Dunnett's test, F(2,13)=17.02, p=0.0002; WT vs KO p=0.0001, WT vs Het p=0.0115). (e) Quantification of cell density of Cux-1+ neurons in the upper cortex (WT=5-9 brains, Het=3-5 brains, KO=2-4 brains; one-way ANOVA, not significant in all regions). (f) Cux-1+ cells differentially distribute in *Taok2* KO cortices with more Cux-1+ cells clustered in the medial to upper portion of the upper cortex in the medial-caudal and dorsal cortical region. Scale bars represent 20μm. (g) Quantification of Cux-1+ cells distribution in the binned upper cortex of frontal, medial and dorsal cortical regions (WT=5-8 brains, Het=3-6 brains, KO=2-5 brains; frontal-rostral: two-way ANOVA, *post hoc* Bonferroni's test, F(2,35)=1.197e-14, p>0.9999 between genotypes; frontal-caudal: two-way ANOVA, *post hoc* Bonferroni's test, F(2,80)=4.657e-13, p>0.9999 between genotypes; WT vs KO: orange p=0.0004; medial-rostral: two-way ANOVA, *post hoc* Bonferroni's test, F(2,45)=4.252e-13, p>0.9999 between genotypes; WT vs KO: orange p=0.0156; medial-caudal: two-way ANOVA, *post hoc* Bonferroni's test, F(2,50)=0.01929,

p=0.9809 between genotypes; WT vs KO: magenta p=0.0003, WT vs Het: magenta p=0.0673, Het vs KO: blue p=0.0118, magenta p<0.0001; dorsal-rostral: two-way ANOVA, *post hoc* Bonferroni's test, F(2,40)=4.764e-13, p>0.9999 between genotypes; WT vs KO: cyan p=0.0287, green p=0.0012; dorsal-caudal: two-way ANOVA, *post hoc* Bonferroni's test, F(2,45)=8.032e-13, p>0.9999 between genotypes; WT vs KO: green p=0.0002, Het vs KO: green p=0.0177). *p<0.05, **p<0.01, ***p<0.001, and ****p<0.0001. Values are mean ± s.e.m).

Supplementary Figure 3. Patterns of oscillatory activity in the prelimbic subdivision of PFC and hippocampal CA1 area of neonatal *Taok2* KO mice. (a) Digital photomontage reconstructing the location of the DiI-labelled recording electrode (orange) in PFC. The superimposed yellow dots mark the 16 recording sites covering the prefrontal subdivisions, cingulate cortex (Cg) and PL. (b) Extracellular LFP recording of discontinuous oscillatory activity in PL from a P7 WT mouse displayed unfiltered (top) and after band-pass (4-100 Hz) filtering (middle). Bottom: traces are accompanied by the color-coded wavelet spectra of the LFP at identical time scale. The red dotted line indicates the lower border of the gamma frequency range (30 Hz). (c) Characteristic theta burst unfiltered (top) and after band-pass (4-100 Hz) filtering (middle). Bottom: Color-coded frequency plots show the wavelet spectra at identical time scale. Note the presence of gamma rhythms superimposed on the underlying theta rhythm. (d) Same as (a) for recording electrode in hippocampal CA1 of a P9 WT mouse. The superimposed yellow dots mark the 16 recording sites covering stratum pyramidale (Str Pyr) and radiatum (Str Rad). (e) Same as (b) for hippocampal LFP from a P8 WT mouse. (f) Characteristic theta burst with sharp wave (red asterisk) displayed unfiltered and after band-pass (4-100 Hz) filtering accompanied by the color-coded frequency plot at identical time scale. Inset, sharp wave reversing between Str Pyr and Str Rad and displayed at a larger time scale. Scale bars for inset correspond to 50 µV and 0.05s. Bar diagrams illustrating the occurrence (g), duration (h), mean amplitude (i) and maximum amplitude (j) of oscillatory events in PFC and HC of WT and *Taok2* KO mice (WT=9 and KO=10-12 mice per group, unpaired t-test, PFC: occurrence t(19)=-0.785, P=0.442; duration t(19)=-3.736, P=0.001; mean amplitude t(19)=-5.393, P=0.00003; maximum amplitude t(19)=-8.245, $P=1.07*10^{-7}$, HC: occurrence t(17)=2.725 ,P=0.014; duration t(17)=-2.759, P=0.013; mean amplitude t(17)=-4.829, P=0.0001; maximum amplitude t(17)=-7.813, $P=5.02*10^{-7}$). (k) Power spectra of discontinuous oscillations in

neonatal PFC when averaged for all WT controls. (l) *Taok2* KO mice have increased power in discontinuous oscillations in theta (4-12Hz), beta (12-30Hz) and gamma (30-100Hz) frequency ranges in PFC (WT=9 and KO=12 mice per group; Shapiro-Wilk test; 4-12 Hz Z=-3.269, P=0.001; 12-30 Hz Z=-3.695, P=0.0002; 30-100 Hz Z=-3.838, P=0.0001). (m) Power spectra of discontinuous oscillations in neonatal HC when averaged for all WT controls. (n) *Taok2* KO mice have increased power in discontinuous oscillations in the PFC in theta (4-12Hz), beta (12-30Hz) and gamma (30-100Hz) frequency ranges (WT=9 and KO=10 mice per group, Shapiro-Wilk test 4-12 Hz Z=-2.939, P=0.003; unpaired t-test 12-30 Hz t(17)=-7.948, P=3.99*10^{-7}, 30-100 Hz t(17)=-7.948, P=3.99*10^{-7}). *p<0.05, **p<0.01, ***p<0.001, and ****p<0.0001. Values are mean +/- s.e.m. (o) Coherence spectra for co-occurring discontinuous network oscillations in neonatal PL and HC when averaged for all WT controls. (p) *Taok2* KO mice have increased coherence between the PFC and HC in the beta frequency (25-40Hz) (WT=9 and KO=10 mice per group; unpaired t-test: t(17)=-2.297, P=0.035). *p<0.05, **p<0.01, and ***p<0.001. Values are mean +/- s.e.m.

Supplementary Figure 4. Hyperactivity and locomotor activity control tests in open-field and anxiety-related behavior for *Taok2* Het and KO mice. (a, b) Distance moved during the open field test displayed in time bins of 5 minutes for female (a) and male (b) mice, respectively, as compared to WT mice within the same time bin. (WT(F)=16, WT(M)=12, Het(F)=17, Het(M)=12, KO(F)=17, KO(M)=9 mice from three different cohorts; three-way mixed ANOVA, *post hoc* Bonferroni; effect of the interaction "genotype x time bin" F(10,385)=2.91, p=0.002; Females: WT vs KO: 15min p<0.05, 25min p<0.001, 30min p<0.01; Males: WT vs KO: 20min p<0.05, 30min p<0.01). (c) Time spent in a 5 cm border of the open field. (WT(F)=16, WT(M)=12, Het(F)=17, Het(M)=12, KO(F)=16, KO(M)=10 mice from three different cohorts; two-way ANOVA, *post hoc* Bonferroni's test, effect of genotype F(2,77)=14.43, p<0.0001; WT vs KO p<0.0001, Het vs KO p=0.0027). (d) Time spent in the center (25 cm x 25 cm) of the open field. (WT(F)=16, WT(M)=12, Het(F)=17, Het(M)=11, KO(F)=17, KO(M)=11 mice from three different cohorts; two-way ANOVA, *post hoc* Bonferroni's test, effect of genotype F(2,77)=6.343, p=0.0028; WT vs KO p<0.0001, Het vs KO p=0.0027). (e-g) Number of closed arm entries (e), time spent in the closed arms (f) and time spent in the center (g) in the elevated plus maze test was not affected by genotype. (WT(F)=10, WT(M)=10, Het(F)=12, Het(M)=12,

KO(F)=9, KO(M)=9 mice from three different cohorts; two-way ANOVA, *post hoc* Bonferroni's test, effect of genotype (e) F(2,56)=0.3404, p=0.7130 (f) F(2,56)=0.2884, p=0.7505 (g) F(2,56)=0.9611, p=0.3887 showing no differences in the number of closed arm entries or time spent in the closed arm or time spent in the center). ns >0.05, *p<0.05, **p<0.01, ***p<0.001 and ****p<0.0001. Values are mean ± s.e.m.

Supplementary Figure 5. Gender control in sociability and locomotor activity controls in social, working and spatial memory and fear learning paradigms for *Taok2* Het and KO mice. (a, b) Time spent sniffing the unfamiliar mouse and novel object the social preference test by female (a) and male (b) mice. (WT(F)=11, WT(M)=11, Het(F)=8, Het(M)=15, KO(F)=12, KO(M)=12 mice from three different cohorts; three-way mixed ANOVA, *post hoc* Bonferroni; effect of the interaction "genotype x stimulus" F(2,63)= 4.96, p=0.001; Females: WT vs KO: Mouse p<0.05, WT: Object vs Mouse p<0.001, Het: Object vs Mouse p<0.01; Males: WT vs KO: Mouse p<0.01 , WT: Object vs Mouse p<0.01, Het: Object vs Mouse p<0.01). (c) Distance moved during the social preference test was not affected by genotype. (WT(F)=11, WT(M)=11, Het(F)=11, Het(M)=15, KO(F)=15, KO(M)=10 mice from three different litters; two-way ANOVA, *post hoc* Bonferroni's test, effect of genotype F(2,67)=0.4582, p=0.6344). (d) Distance moved during the recall trial of the spatial object recognition test was not affected by genotype. (WT(F)=11, WT(M)=11, Het(F)=11, Het(M)=15, KO(F)=15, KO(M)=10 mice from three different litters; two-way ANOVA, *post hoc* Bonferroni's test, effect of genotype F(2,67)=0.4396, p=0.6462). (e) No effects of genotype was detected for the time required to do a transition during the spontaneous alternation test in the Y-maze, although average values indicate that KO tended to be faster than WT. (WT(F)=11, WT(M)=11, Het(F)=15, Het(M)=13, KO(F)=16, KO(M)=8 mice from three different cohorts; two-way ANOVA, *post hoc* Bonferroni's test, effect of genotype F(2,68)=0.1177, p=0.8891). (f) No correlation was detected between time required to do a transition and percentage of alternation in the spontaneous alternation test in the Y-maze. (g, h) No difference between genotype was detected in their time spent immobile during baseline and conditioning trial of the contextual fear conditioning test as tested for female (g) and male mice (h). (WT(F)=9, WT(M)=8, Het(F)=9, Het(M)=9, KO(F)=9, KO(M)=8 mice from three different cohorts; three-way mixed ANOVA, *post hoc* Bonferroni,

with no difference in the time spent immobile during the baseline and conditioning trial. ns >0.05, *$p<0.05$, **$p<0.01$ and ***$p<0.001$. Values are mean ± s.e.m.

Supplementary Figure 6. Learning curves during training in the water maze test of WT and *Taok2* Het and KO mice. Mice were trained for eight trials over two days (4 trials per day, inter trial interval of 10 min) to find a hidden platform in a circular water maze. No effect of genotype, nor of the interaction "genotype x sex", "genotype x trial" or "genotype x sex x trial" was detected as measured with genotype and sex as between groups factor and trial as within group factor. (a) Distance swum to reach the platform by female (left panel) and male (right panel) mice. (b) Escape latency of female (left panel) and male (right panel) mice. (c) Mean swimming velocity of female (left panel) and male right panel) mice. (d) Time spent in the border (a 10 cm annulus adjacent to the poll wall) of female (left panel) and male (right panel) mice. (WT(F)=12, WT(M)=10, Het(F)=12, Het(M)=11, KO(F)=12, KO(M)=12 mice from three different cohorts; three-way mixed ANOVA, *post hoc* Bonferroni; effect of the interaction "genotype x trial" showed no difference in the learning curves with no difference in total distance swum, mean velocity, and time spent at the border. ns $p>0.05$. Values are mean ± s.e.m.

Supplementary Figure 7. Taok2 deficient mice show enhanced preference for platform position in Morris water maze. Tests were conducted during the transfer trial performed 24 hours after the last training session. (a) Percentage of time spent in the four quadrants for female (left panel) and male (right panel) mice. Dotted lines indicate chance level (25%). (b) Number of crossings of imaginary platforms equally located in the center of each quadrant for female (left panel) and male (right panel) mice. (c). Time spent in imaginary platforms equally located in the center of each quadrant for female (left panel) and male (right panel) mice. (WT(M)=10, Het(F)=12, Het(M)=11, KO(F)=12, KO(M)=9 mice from three different cohorts; three-way mixed ANOVA, *post hoc* Bonferroni; effect of the interaction "genotype x position" (a) $F(6,180)=3.3$, $p=0.0042$,; Females/Target: WT vs KO $p<0.05$ (b) $F(6,180)=4.9105$, $p=0.0001$; Females/Target: WT vs KO $p<0.05$, WT vs Het $p<0.05$, Males/Target: WT vs KO $p<0.01$ (c) $F(6,180)=4.495$, $p=0.0003$; Females/Target: WT vs KO $p<0.01$, Males/Target: WT vs KO $p<0.01$ showing significant effects of the interaction "genotype x position" with genotype and

sex as between groups factors and quadrant or platform position as within groups factor. ns>0.05, *p<0.05 and **p<0.01. Values are mean ± s.e.m.

Supplementary Figure 8. *Taok2* Het and KO mice have milder reduction in dendrite growth and synaptic connectivity in the somatosensory cortex. (a) Top: Golgi-stained SSC neurons from P21 WT and *Taok2* Het and KO mice. Scale bars represent 20µm. Bottom: Dendritic heat maps of superimposed neuron tracings for each condition. Blue to red (apical) and yellow to blue (basal) indicates increased probability of dendrite presence. . Scale bars represent 20µm. (b) Top: No major difference in apical dendritic complexity in in layer 2 SSC neurons in *Taok2* Het and KO mice (WT=16, Het=17 and KO=15 neurons from 3 different brains; two-way ANOVA, *post hoc* Dunnett's test; $F(2,2115)=0.8267$, $p=0.4376$ between genotypes; *represents ranges of significance; WT vs Het (blue), WT vs KO (red); see supplemental statistics). Bottom: Less significant reduction in basal dendritic complexity in layer 2/3 SSC neurons in *Taok2* Het and KO mice compared to WT mice (WT=19, Het=18, and KO=18 neurons from 3 different brains; two-way ANOVA, *post hoc* Dunnett's test; $F(2,1204)=17.92$, $p<0.0001$ between genotypes; *represents ranges of significance; WT vs KO (red); see supplemental statistics). (c) Top: No significant difference in apical and basal dendrite length between WT and *Taok2* Het and KO SSC neurons (apical: WT=15, Het=17 and KO=15 neurons from 3 different mice brains; one-way ANOVA, *post hoc* Dunnett's; $F(2,44)=0.4811$, $p=0.6213$; basal: WT=19, Het=12 and KO=16 neurons from 3 different mice brains; one-way ANOVA, *post hoc* Dunnett's; $F(2,44)=1.32$, $p=0.2775$). (d) Dendritic spine distribution maps of P21 Golgi-stained WT and *Taok2* KO SSC neurons. (e) No significant change in spine distribution between WT and *Taok2* Het and KO SSC neurons (WT=6, Het=6, and KO=6 neurons from 3 different brains; two-way ANOVA, *post hoc* Dunnett's test; $F(2,615)=1.905$, $p=0.1497$ between genotypes). (f) No significant difference in apical and basal dendritic spines per cell between WT and *Taok2* Het and KO SSC neurons (WT=6, Het=6, and KO=6 neurons from 3 different mice brains; Left: one-way ANOVA, *post hoc* Dunnett's test; $F(2,15)=0.3927$, $p=0.6820$; Right: one-way ANOVA, *post hoc* Dunnett's test; $F(2,15)=0.1042$, $p=0.9017$). (g) Cumulative probability histograms show shift towards reduced dendritic spine lengths (right) *Taok2* KO SSC neurons and reduced head widths (left) in *Taok2* Het and KO SSC neurons. (h) *Taok2* Het and KO SSC neurons have a significant increase in thin spines and reduction in mushroom-like spines when compared to WT

SSC neurons (WT = 4111 spines, Het = 3688, KO = 3729 spines from 6 cells per condition from three different brains; two-way ANOVA, *post hoc* Sidak's test; $F(2,90)=1.964e-13$, $p>0.9999$ between genotypes; WT vs KO F: $p=0.8822$, LT: $p=0.9557$, T: $p=0.0002$, S: $p=0.2832$, M: $p<0.0001$, and B: $p>0.9999$; WT vs Het F: $p=0.7578$, LT: $p=0.3811$, T: $p=0.0291$, S: $p=0.9864$, M: $p=0.0044$, and B: $p=0.9987$). (i) *Taok2* KO SSC neurons imaged by electron microscopy show decreased percentage of synapses on postsynaptic spines (left) and increased synapses on dendrites (right) (WT=95, KO=65 neurons; left: unpaired t-test; $t(162)=7.261$, $p<0.0001$; right: $t(162)=8.839$, $p<0.0001$). (j) Representative traces of mEPSC spikes from WT and *Taok2* KO SSC neurons. Scale: 5pA by 1sec. (k) Left: Longer inter-event intervals in *Taok2* Het and KO SSC neurons shown on a cumulative probability histogram. Inside: Reduced mEPSC event frequency in *Taok2* Het and KO SSC neurons (WT=13, Het=9 and KO=11 neurons from 3 different mice brains; one-way ANOVA, *post hoc* Dunnett's test; $F(2,31)=12.86$, $p<0.0001$; WT vs Het $p=0.0088$, WT vs KO $p<0.0001$). Right: No change in mEPSC amplitude in *Taok2* WT, Het and KO SSC neurons shown on a cumulative probability histogram. Inside: Average mEPSC amplitude of *Taok2* WT, Het and KO neurons (WT=12, Het=9 and KO=13 neurons from 3 different mice brains; kruskal-walis ANOVA, *post hoc* Dunn's test; H-value=0.01588, $p=0.9921$; WT vs Het $p>0.9999$, WT vs KO $p>0.9999$). *$p<0.05$, ***$p<0.001$ and ****$p<0.0001$. Values are mean +/- s.e.m.

Supplementary Figure 9. TAOK2 KO mice have no alterations in dendrite growth and synaptic connectivity in the hippocampus. (a) Top: Golgi-stained CA1 HC neurons from P21 WT and *Taok2* Het and KO mice. Scale bars represent 20µm. Bottom: Dendritic heat maps of superimposed neuron tracings for each condition. Blue to red (apical) and yellow to blue (basal) indicates increased probability of dendrite presence. . Scale bars represent 30µm. (b) Top: No major difference in apical and basal dendritic complexity in in layer 2 HC neurons in *Taok2* Het and KO mice (apical: WT=15, Het=14 and KO=18 neurons from 3 different brains; two-way ANOVA, *post hoc* Dunnett's test; $F(2,2156)=2.156$, $p=0.1160$ between genotypes, WT vs Het $p=0.0202$ at 35µm). Bottom: No significant change in basal dendritic complexity in CA1 HC neurons in *Taok2* Het and KO mice compared to WT mice (WT=18, Het=15, and KO=16 neurons from 3 different brains; two-way ANOVA, *post hoc* Dunnett's test; $F(2,1472)=7.277$, $p=0.0007$ between genotypes). (c) No significant difference in apical and basal dendrite length

between WT and *Taok2* Het and KO HC neurons (Apical: WT=15, Het=14, and KO=18 neurons from 3 different mice brains; one-way ANOVA, *post hoc* Dunnett's test; F(2,44)=0.3342, p=0.7177. Basal: WT=23, Het=16, and KO=22 neurons from 3 different mice brains; one-way ANOVA, *post hoc* Dunnett's test; F(2,58)=0.4335, p=0.6503). (d) Dendritic spine distribution maps of P21 Golgi-stained WT and *Taok2* KO HC neurons. (e) No major change in spine distribution between WT and *Taok2* Het and KO HC neurons (WT=6, Het=6, and KO=6 neurons from 3 different mice brains; Two-way ANOVA, *post hoc* Dunnett's test; F(2, 600)=2.636 between genotypes, p=0.0725; WT vs KO 65µm p=0.0363). (f) No significant difference in apical and basal dendritic spines per cell between WT and *Taok2* Het and KO HC neurons (WT=6, Het=6, and KO=6 neurons from 3 different mice brains; Left: one-way ANOVA, *post hoc* Dunnett's test; F(2,15)=1.445, p=0.1668); Right: one-way ANOVA, *post hoc* Dunnett's test; F(2, 15)=0.5609, p=0.5822|). (g) Cumulative probability histograms show no shift in dendritic spine lengths (right) and head widths (left) in *Taok2* Het and KO HC neurons. (h) No significant difference in spine type between WT and *Taok2* KO HC neurons (WT = 7032, Het = 6747, and KO = 7489 spines from 6 cells per condition from three different brains; two-way ANOVA, *post hoc* Dunnett's test; F(2,90)=1.317e-13, p>0.9999 between genotypes). (i) *Taok2* KO neurons imaged by electron microscopy shows no change in percentage of synapses on postsynaptic spines (left) or dendrites (right) (WT=81, KO=60 neurons; *left*: unpaired t-test; t(139)=0.7799, p=0.4368; *right*: t(139)=0.7245, p=0.4700). (j) Western blot of pTAOK2 and TAOK2 levels in different regions of P21 *Taok2* WT, Het and KO mice brains (Cortex, Hippocampus, Striatum, Thalamus/Hypothalamus, Midbrain, Cerebellum, Pons/Medulla, Total Brain). Arrow head indicates pTAOK2 bands. Arrow indicates unspecific band. (k) The pTaok2/Taok2 ratio in the HC is significantly reduced compared to the cortex, (CTX=5, HC=5, STR=4, T/HT=5, MB=5, CB=4 and P/M=3 brains from 3 different litters; one-way ANOVA, *post hoc* Bonferroni's test; F(6,24)=6.767, p=0.0003; CTX vs HC *p=0.0145). *p<0.05. Values are mean +/- s.e.m.

Supplementary Figure 10. No change in mIPSC frequency and amplitude in PFC and SSC neurons in *Taok2* KO mice. (a) Representative traces of mIPSC spikes from WT and *Taok2* KO PFC neurons. Scale: 10pA by 1sec. (b) No change in inter-event intervals between WT and *Taok2* KO PFC neurons shown on a cumulative probability histogram. Inside: No change in

mean mIPSC event frequency between WT and *Taok2* KO PFC neurons (WT=14 and KO=14 neurons from 3 different mice brains; unpaired t-test; t(26)=0.03925, p=0.9690). (c) No change in mean mIPSC amplitude between WT and *Taok2* KO PFC neurons shown on a cumulative probability histogram. Inside: No change in average mIPSC amplitude between WT and *Taok2* KO PFC neurons (WT=14 and KO=14 neurons from 3 different mice brains; unpaired t-test; t(26)=1.117, p=0.2742). (d) Representative traces of mIPSC spikes from WT and *Taok2* KO SSC neurons. Scale: 10pA by 1sec. (e) No change in inter-event intervals between WT and *Taok2* KO SSC neurons shown on a cumulative probability histogram. Inside: No change in mean mIPSC event frequency between WT and *Taok2* KO SSC neurons (WT=14 and KO=12 neurons from 3 different mice brains; unpaired t-test; t(24)=0.2274, p=0.8221). (f) No change in mIPSC amplitude between WT and *Taok2* KO SSC neurons shown on a cumulative probability histogram. Inside: No change in mean mIPSC event frequency between WT and *Taok2* KO SSC neurons (WT=14 and KO=12 neurons from 3 different mice brains; unpaired t-test; t(24)=0.09472, p=0.9253). Values are mean +/- s.e.m.

Supplementary Figure 11. TAOK2 is enriched in the post synaptic density and loss of Taok2 decreases synaptic density and stability. (a) Co-staining of Taok2 and SynGAP on DIV18-19 cortical neurons. Scale bars represent 10µm. (b) Western blot of synaptosome fractions (Homogenized fraction (Homo), Cell pellet (Pellet), Synaptoneurosome fraction (SN), Synaptic fraction (Syn) and post-synaptic density fraction (PSD)) from 1-month-old WT mice shows that Taok2 is enriched in the PSD fraction. (c) Co-staining of WT and *Taok2* KO DIV18-19 cortical neurons with phalloidin and SynGAP. Scale bars represent 10µm. (d) Cultured KO cortical neurons have decreased number of spines per cell compared to WT neurons (WT=15 and KO=29 neurons from 3 different cultures; unpaired t-test; t(42)=2.427 p=0.0196). (e) Cultured *Taok2* KO cortical neurons have decreased number of SynGAP and phalloidin positive spines per cell compared to WT neurons (WT=15 and KO=29 neurons from 3 different cultures; unpaired t test; t(42)=2.099 p=0.0418). (f) Cortical neurons transfected with GFP and control shRNA or TAOK2 shRNA at various dosages (0.25, 0.50, and 0.75µg) Scale bars represent 10 µm. (g) Cortical neurons transfected with TAOK2 shRNA show reduced dendritic spine density (spines/10µm dendrites) compared to EGFP and control shRNA transfected neurons (EGFP=25, ctrl=22, shTAOK2(0.25µg)=21, shTAOK2(0.50µg)=14, shTAOK2(0.75µg)=16 cells from three

different cultures; one-way ANOVA; *post hoc* Dunnett's test; $F(4,93)=18.22$, $p<0.0001$; EGFP vs shTAOK2(0.25μg) $p<0.0001$, EGFP vs shTAOK2(0.50μg) $p=0.0001$, EGFP vs shTAOK2(0.75μg) $p<0.0001$). (h Images of DIV24 hippocampal neuron cultures transfected with only GFP (control) or TAOK2 shRNA. Boxes are magnified and shown at 0 min, 152min and 302min. Scale bars represent 10 μm. (i) Images of DIV24 hippocampal neuron cultures transfected with Lifeact-GFP or TAOK2 shRNA and Lifeact-GFP. Boxes are magnified and showed to the right. Kymographs show increased movement of dendritic spines on neurons with acute loss of Taok2 (Bottom right). Scale bars represent 10 μm. ns $p>0.05$, *$p<0.05$, ***$p<0.001$ and ****$p<0.0001$. Values are mean +/- s.e.m.

Supplementary Figure 12. Location of all *de novo* and rare-inherited mutations on *TAOK2* and pedigrees of *de novo* and truncation mutation probands. (a) Top: Detailed diagram of TAOK2α and TAOK2β isoforms and location of *de novo,* truncating, and rare-inherited mutations. Different protein domains are represented by colored boxes (kinase domain: red, MEK binding domain: black, regulatory domains: blue and light blue. TAOK2α has two phosphorylation sites (ser181 and thr475) and caspase-9 cleavage site (^{916}DPGD^{919}). TAOK2β has 3 known phosphorylation sites (ser181, thr475, and ser1031). Bottom: Diagrams of truncated TAOK2 due to the P1022*, Q622*, T604Sfs*45 and L1030Wfs*3 mutations. Dark red box represent frame shifts before introduction of premature stop. P1022* only effects the β isoform and L1030Wfs*3 only effects the α isoform. (b) Family pedigrees of 3 *de novo* and 3 truncating mutations. Families include 01-0337 (A135P/+), 07-0179 (c.563+12_563+15/+), 01-0559 (P1022*), 01-0446 (L1030Wfs*3), AU4112 (T604Sfs*3) and AU4261 (Q622*). Black filled squares represent individuals diagnosed with autism spectrum disorder. Genotype details of the AU4122 and AU4261 family members are not available.

Supplementary Figure 13. *TAOK2* mRNA expression analysis of 01-0337, 07-0179, and 01-0559 families. (a) No changes in *TAOK2* expression in LCLs from the A135P, P1022* and c.563+12_563+15 probands compared to average of controls (3 passages and 2 technical replicates; one-way ANOVA, *post hoc* Dunnett's test; $F(11,29)=3.928$, $p=0.0015$; Control vs P1022* father $p=0.0327$, Control vs P1022* affected sibling $p=0.0438$). (b) No changes in *TAOK2β* isoform-specific expression in LCLs from the A135P, P1022* and c.563+12_563+15

probands compared to average of controls (3 passages and 2 technical replicates; one-way ANOVA, *post hoc* Dunnett's test; $F(11,29)=2.841$, $p=0.0120$). RNA expression was measured using ddPCR and normalized to TBP mRNA levels. Parents and siblings of the 3 probands were used as controls for *TAOK2* expression. (c) c.563+12_563+15 proband have 13% fold increase in intron 7 retention. *$p<0.05$. Values are mean +/- s.e.m.

Supplementary Figure 14. The effect of the A135P mutation on TAOK2α function and localization (a) Western blot of HEK 293 cell lysates 48 hours post transfection with TAOK2α, TAOK2α A135P, and TAOK2α A335V. (b) TAOK2α A135P has reduced ser181 phosphorylation (right) compared to TAOK2α, while protein levels (left) remain unchanged. TAOK2α A335V shows no difference compared to TAOK2α. (n=5 western blots; one-sample t-test; TAOK2 levels: A135P: $t(4)=1.0171$, $p=0.3444$, A335V: $t(4)=1.151$, p0.3138; pTAOK2 levels: A135P: $t(4)=15.80$, $p<0.0001$), A335V: $t(4)=0.05114$, $p=0.9617$. (c) Representative western blot of HEK293 cell lysates 48 hours post transfection with JNK1a1 only or with TAOK2α, TAOK2α A135P. (d) TAOK2α phosphorylates JNK to a higher degree (1757% ± 505) than TAOK2β (384% ± 89). TAOK2α A135P significantly decreases phosphorylation of JNK1a1 in HEK 293 cells compared to TAOK2α (n=5 western blots; TAOK2α vs TAOK2α A135P: unpaired t-test; $t(8)=3.138$, $p=0.0138$; Jnk1a1 vs TAOK2α: one-sample t-test, $t(4)=3.282$, $p=0.0304$). (e) Top: Staining of TAOK2 (red) on DIV14 cortical neurons transfected with palmitoylated GFP shows that TAOK2α localizes with tubulin (green). Bottom: TAOK2α (green) does not localize with F-actin rich protrusions (stained with rhodamine-labelled phalloidin; red). Scale bars represent 10µm. White boxes are magnified and shown to the right, with scale bars representing 3µm. (f) Top: Staining of TAOK2 (red) on DIV14 cortical neurons shows that TAOK2α A135P does not affect localization with tubulin (green). Bottom: TAOK2α A135P (green) also does not localize with F-actin rich protrusions (stained with rhodamine-labelled phalloidin; red). Scale bars represent 10µm. White boxes are magnified and shown to the right, with scale bars representing 3µm. All neurons are co-stained with DAPI. *$p<0.05$ and ****$p<0.0001$. Values are mean +/- s.e.m.

Supplementary Figure 15. P1022* *de novo* mutation does not affect TAOK2 auto-phosphorylation and stability and RhoA levels in LCLs compared to family members (a)

Western blot of LCL lysates from the P1022* proband and their family members. (b) No significant difference in TAOK2 (left) and pTAOK2 (right) levels in the P1022* proband compared to the rest of the family, where the mother is set at 100% (n=6 western blots; TAOK2 levels: one-sample t-test; 100% vs P1022*: $t(5)=2.023$, $p=0.0989$; pTAOK2 levels: 100% vs P1022*: $t(5)=0.3183$, $p=0.7631$) (c) Western blot of LCL lysates from the P1022* proband and their family members after RhoA-GTP pull-down. (d) No significant difference in RhoA-GTP (left) and RhoA (right) levels in the P1022* proband compared to the rest of the family, where the mother is set at 100% (n=6 western blots; RhoA-GTP levels: one-sample t-test; RhoA-GTP levels: 100% vs P1022*: $t(5)=0.5872$, $p=0.5826$; RhoA levels (normalized to tubulin): one-sample t-test; 100% vs P1022*: $t(5)=1.683$, $p=0.1532$). Values are mean +/- s.e.m.

Supplementary Figure 16. P1022* elongates basal dendrites and TAOK2 A135P and P1022* reduce dendrite integrity. (a) TAOK2β P1022* increases dendrite length in WT *Taok2* cortical neurons (6 neurons from 3 different brains per condition; one-way ANOVA, *post hoc* Dunnett's test; $F(3,20)=19.02$, $p<0.0001$; TAOK2α/β vs β P1022* $p<0.0001$). (b) Images of dendrite branching in cortical neurons from *in utero* electroporated WT *Taok2* P21 mice. Red arrow heads indicate the measurements on dendrites after branching and red bars highlight the width of dendrites in that region. Scale bars represent 3μm. (c) TAOK2α/β A135P and TAOK2β P1022* reduce dendrite thickness after branching (6 neurons from 3 different brains per condition; one-way ANOVA, *post hoc* Dunnett's test; 1st: $F(3,168)=18.72$, $p<0.0001$; Venus vs TAOK2α/β $p=0.0062$, TAOK2α/β vs α/β A135P $p<0.0001$, TAOK2α/β vs β P1022* $p<0.0001$; 2nd: $F(3,265)=18.6$, $p<0.0001$; TAOK2α/β vs α/β A135P $p<0.0001$, TAOK2α/β vs β P1022* $p<0.0001$; 3rd: $F(3,274)=23.75$, $p<0.0001$; TAOK2α/β vs α/β A135P $p<0.0001$, TAOK2α/β vs β P1022* $p<0.0001$; 4th: $F(3,164)=24.61$, $p<0.0001$; TAOK2α/β vs α/β A135P $p<0.0001$, TAOK2α/β vs β P1022* $p<0.0001$; 5th: $F(3,74)=7.031$, $p=0.0003$; TAOK2α/β vs β P1022* $p=0.0002$).

Supplementary Figure 17. RhoA interacts with Taok2 in the mouse cortex and preferentially with TAOK2β isoform in HEK239 cells. (a) Western blot of Taok2 immunoprecipitation (IPs) from a crude homogenate (H) and a crude membrane fraction (Pellet fraction 1, P1) from WT mouse cortices show direct or indirect interaction of Taok2 and RhoA.

Membranes were immunoblotted against Taok2, RhoA and β-actin (loading control). Unspecific goat IgGs were used as IP controls. (b) Western blot of RhoA immunoprecipitations (IPs) from lysates from HEK293 cells transfected with GFP or TAOK2α and β variants. Lysates were blotted against pTAOK2, TAOK2, RhoA, and β-actin as loading control. (c) Quantification of Taok2 co-immunoprecipitation with RhoA from HEK293 cells (shown in b) reveals that TAOK2β preferentially binds RhoA. % of TAOK2β bound to RhoA was set to 100%. TAOK2β P1022*, TAOK2α, and TAOK2α A135P has significantly reduced binding to RhoA compared to TAOK2β. (N=3 western blots, one-way Anova, post hoc Dunnett's test; $F(4,10)=12.75$, $p=0.0006$; TAOK2β vs TAOK2β A135P $p=0.3245$, TAOK2β vs TAOK2β P1022* $p=0.0118$, TAOK2β vs TAOK2α $p=0.0007$, TAOK2β vs TAOK2α A135P $p=0.0006$).

Supplementary Figure 18. The role of human-derived TAOK2 mutations identified in ASD probands at the synapse. (a) TAOK2α associates with microtubules, while TAOK2β localizes to dendritic spines. It is suggested that TAOK2β increases levels of active RhoA-GTP, which controls stability of the actin cytoskeleton and dendritic spine. The A135P mutation in TAOK2α does not alter localization to microtubules, while TAOK2β A135P cannot localize to dendritic spines resulting in a LOF phenotype. It is suggested that the TAOK2β P1022* has increased kinase activity and thus may increase phosphorylation of JNK leading to elongation of microtubules and dendrites. TAOK2β P1022* also does not localize to dendritic spines. Phosphorylation of JNK1 by TAOK2α A135P is also disrupted. (b) Flow chart describing the characterization of Taok2 KO mice by studying changes in behavior, brain morphology, and functional cellular changes in cortical neurons, cell lines, and human cell samples. This is combined with the identification of *de novo* and rare-inherited mutation in TAOK2 by whole exome/genome sequencing of ASD families. These human-derived mutations were further studied using functional *in vitro* assays using murine and human cell systems, cortical neuron cultures derived from Taok2 KO mice as well as *in vivo* assays in *Taok2* Het mice.

Video 1. Time-lapse imaging of 14DIV neurons expressing control shRNA and TAO2 shRNA together with palmitoylated **GFP plasmid.** Epi-fluorescence imaging was performed on an inverted Nikon microscope (Eclipse, Ti) with a 60x objective (NA 1.4). Duration of time-lapse imaging: 5 min acquiring images every 2 sec.

Video 2. Time-lapse imaging of 14DIV neurons expressing TAO2β, TAOβ A135P, TAOβ P1022 together with palmitoylated **GFP plasmid.** Epi-fluorescence imaging was performed on an inverted Nikon microscope (Eclipse, Ti) with a 60x objective (NA 1.4). Duration of time-lapse imaging: 5 min acquiring images every 2 sec.

Video 3. Time-lapse imaging of 14DIV TAO2 KO neuron expressing Lifeact-GFP plasmid. Cell was imaged before CN01 treatment for 5 min (acquiring images every 2 sec). After CN01 treatment the same cell was imaged for 5 min (acquiring images every 2 sec). Epi-fluorescence imaging was performed on an inverted Nikon microscope (Eclipse, Ti) with a 60x objective (NA 1.4).
